# Design and Synthesis of Dihydroxamic Acids as HDAC6/8/10 Inhibitors

**DOI:** 10.1002/cmdc.202000149

**Published:** 2020-05-13

**Authors:** Michael Morgen, Raphael R. Steimbach, Magalie Géraldy, Lars Hellweg, Peter Sehr, Johannes Ridinger, Olaf Witt, Ina Oehme, Corey J. Herbst‐Gervasoni, Jeremy D. Osko, Nicholas J. Porter, David W. Christianson, Nikolas Gunkel, Aubry K. Miller

**Affiliations:** ^1^ Cancer Drug Development Group German Cancer Research Center (DKFZ) Im Neuenheimer Feld 280 69120 Heidelberg Germany; ^2^ Faculty of Biosciences University of Heidelberg 69120 Heidelberg Germany; ^3^ Chemical Biology Core Facility European Molecular Biology Laboratory (EMBL) 69117 Heidelberg Germany; ^4^ Hopp Children's Cancer Center Heidelberg (KiTZ) 69120 Heidelberg Germany; ^5^ Clinical Cooperation Unit Pediatric Oncology German Cancer Research Center (DKFZ) 69120 Heidelberg Germany; ^6^ Department of Pediatric Oncology Hematology and Immunology University Hospital Heidelberg 69120 Heidelberg Germany; ^7^ German Cancer Consortium (DKTK) 69120 Heidelberg Germany; ^8^ Roy and Diana Vagelos Laboratories Department of Chemistry University of Pennsylvania Philadelphia PA 19104-6323 USA

**Keywords:** HDAC8, HDAC10, inhibitors, polypharmacology, targeted therapy

## Abstract

We report the synthesis and evaluation of a class of selective multitarget agents for the inhibition of HDAC6, HDAC8, and HDAC10. The concept for this study grew out of a structural analysis of the two selective inhibitors Tubastatin A (HDAC6/10) and PCI‐34051 (HDAC8), which we recognized share the same *N*‐benzylindole core. Hybridization of the two inhibitor structures resulted in dihydroxamic acids with benzyl‐indole and ‐indazole core motifs. These substances exhibit potent activity against HDAC6, HDAC8, and HDAC10, while retaining selectivity over HDAC1, HDAC2, and HDAC3. The best substance inhibited the viability of the SK‐N‐BE(2)C neuroblastoma cell line with an IC_50_ value similar to a combination treatment with Tubastatin A and PCI‐34051. This compound class establishes a proof of concept for such hybrid molecules and could serve as a starting point for the further development of enhanced HDAC6/8/10 inhibitors.

## Graphical abstract

**Best of both worlds**: A new HDAC inhibitor class has been developed by hybridizing two structurally related, but functionally distinct, inhibitors: PCI‐34051 (HDAC8 inhibitor) and Tubastatin A (HDAC6/10 inhibitor). The resulting dihydroxamic acids are capable of inhibiting HDACs 6, 8, and 10, and are characterized by a benzyl‐indole/indazole scaffold, which functions as both cap and linker group.

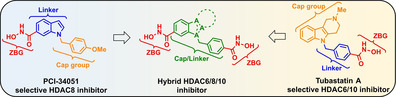

## Introduction

In the past decades, drug discovery efforts have focused intensely on the development of inhibitors with high target selectivity. At the same time it is well recognized that successful drugs typically exhibit polypharmacology, and that the “one‐target‐one‐disease” approach often oversimplifies the complex biology underlying most pathologies.^1^ Combination therapy approaches against multiple targets are clinically successful, but there are advantages to developing a single drug that engages multiple targets, particularly when mono‐targeted drugs are not already clinically available. Such advantages include guaranteed action against both targets in drug‐exposed tissues, as well as simplified pharmacodynamics, manufacture, and regulatory approval.^2^

Histone deacetylases (HDACs) regulate the acetylation state of lysine residues of histones as well as other protein substrates, and therefore play a pivotal role in many cellular processes. Modulation of HDAC activity with inhibitors is known to be effective in treating different pathologies, and four HDAC inhibitor (HDACi) drugs have been approved by the FDA with many more being evaluated in clinical studies.^3^

Many HDACi, including the four approved drugs, inhibit most HDAC isozymes and are known to have severe side effects, particularly due to inhibition of HDACs 1, 2, and 3.^4^ Isozyme‐specific HDACi are expected to alleviate these liabilities, and numerous selective HDACi have been described.^5^ Specifically targeting two or more distinct HDACs can also be beneficial, but this presents a particular challenge when those two HDACs belong to different isozyme classes with different structural requirements for efficient binding (the Zn^2+^‐dependent HDACs are grouped into Class I (HDACs 1,2,3,8), Class IIa (HDACs 4,5,7,9), Class IIb (HDACs 6,10) and Class IV (HDAC11)). Such a situation exists for late‐stage neuroblastoma, where high HDAC8 (Class I) and HDAC10 (Class IIb) expression levels strongly correlate with poor outcomes, and the two enzymes are considered as targets for treatment.^6^ On the one hand, inhibition and knock‐down of HDAC8 favors cell‐cycle arrest and differentiation, retards cell growth, and induces cell death in vitro and in vivo.^7^ On the other hand, inhibition and knock‐down of HDAC10 halts autophagic flux and impairs DNA damage repair mechanisms, leading to an increased sensitivity to chemotherapy.^8^ Furthermore, simultaneous inhibition of HDAC8 and HDAC10 has been shown to be effective in killing neuroblastoma cells alone and in combination with retinoic acid treatment.^6^

HDACi are usually described as containing three structural modules: a zinc‐binding group (ZBG), a “linker” moiety, and a “cap group”. The cap groups in most HDACi are solvent exposed and often tolerate a variety of chemical modifications. Arming of HDACi cap groups with other targeted scaffolds to make chimeras has been particularly successful, producing combination HDAC‐IDO1,^9^ ‐proteasome,^10^ ‐PDE5,^11^ ‐kinase,^12^ ‐IMPDH,^13^ ‐BET,^14^ ‐SERM,^15^ ‐topoisomerase,^16^ and other inhibitors.^17^ HDACi have also been incorporated into PROTACs.^18^ We envisioned developing a new HDACi, with activity against HDAC8 and HDAC10 by combining two isozyme‐specific and highly potent HDAC inhibitors into a chimeric inhibitor.

**Figure 1:**
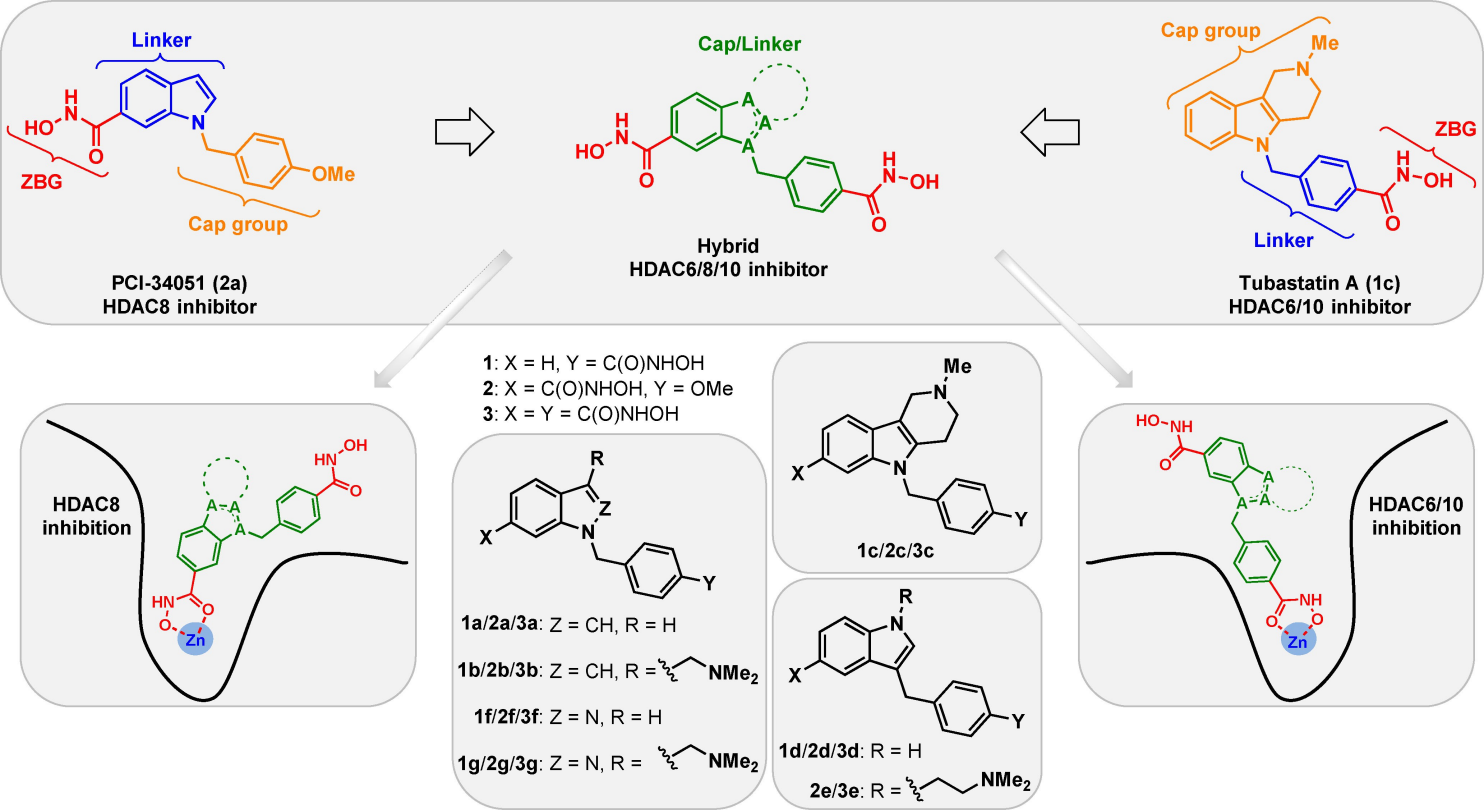
Concept to merge the scaffolds of PCI‐34051 and Tubastatin A. Top: PCI‐34051 (left) and Tubastatin A (right) are depicted with their zinc binding groups (ZBG) in red, linkers in blue, and cap groups in orange. Recognition that both inhibitors share an *N*‐benzylindole scaffold inspired the design of hybrid inhibitors (center) with two ZBGs (red) and a central core that would function as both cap group and linker (green). Lower left and right: Inhibition of HDAC8 and HDAC6/10 would result from engagement of one of the two ZBGs. Lower center: Synthesized mono‐ and hybrid dihydroxamic acids used in this study.

We recently showed that Tubastatin A, which is annotated as a selective HDAC6 inhibitor, is also a highly potent HDAC10 binder.^19^ We additionally recognized that the selective HDAC8 inhibitor PCI‐34051^20^ bears a structural similarity to Tubastatin A: both compounds share an *N*‐benzylindole core. Whereas, in PCI‐34051 (Figure 1, top left) the indole moiety (blue) functions as the linker with a ZBG at C6, it is part of the γ‐carboline cap group (orange) of Tubastatin A (Figure 1, top right). Similarly, the *N*‐benzyl moiety in PCI‐34051 (orange) is the cap group, while functioning as the linker (blue) for Tubastatin A. Interestingly, the two compounds present their ZBGs at opposing positions of this core, which is presumably responsible for their very different selectivity profiles. We postulated that these two known inhibitors could be merged, to make a hybrid HDACi (Figure 1, top middle). Because Tubastatin A inhibits both HDAC6 and HDAC10, these hybrids would likely be HDAC6/8/10 inhibitors.^6^ While HDAC6 expression does not significantly correlate with prognosis in neuroblastoma,7a, 21 HDAC6 inhibitors have been found to be well tolerated in clinical studies^22^ and selective HDAC6 inhibition has been shown to be non‐cytotoxic in cancer settings.^23^ We therefore allowed HDAC6 inhibition as a feature of our compounds.

In our strategy, the hybrid inhibitors would contain two ZBGs, each one responsible for selectively binding to different enzymes. Thus, the hydroxamic acid on the heterocycle would serve as the ZBG with respect to HDAC8 inhibition (Figure 1, lower left), with the phenyl hydroxamate functioning as part of the cap group. The situation would be reversed in the case of HDAC6/10 inhibition (Figure 1, lower right).

We have previously demonstrated that a basic nitrogen in the cap group of Tubastatin A analogs is important for potent HDAC10 binding, probably via hydrogen bonding with gatekeeper residue E272.^19^ Therefore, we expected that a hybrid inhibitor bearing a PCI‐34051‐like indole, with no substitution at C2 or C3 of the indole, would be unlikely to give potent HDAC10 activity. Little information is available in the literature with respect to SAR around the PCI‐34051 linker indole, but we postulated that the bulky γ‐carboline cap group of Tubastatin A might be too large to serve as linker (linkers are usually relatively slender) for a hybrid inhibitor. We therefore synthesized a variety of phenyl‐hydroxamic acids **1a**–**1g** as HDAC6/10 inhibitors and indolyl/indazolyl‐hydroxamic acids **2a**–**2g** as HDAC8 inhibitors to serve as benchmark comparisons to the corresponding dihydroxamic acids **3a**–**3g**, which should inhibit HDAC6/8/10 (Figure 1, bottom middle). In this numbering scheme, PCI‐34051 is labelled as **2a** and Tubastatin A as **1c**.

## Chemistry

**Scheme 1:**
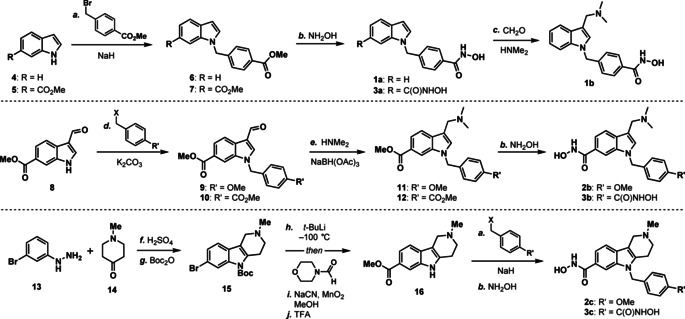
Synthesis of N‐benzylated derivatives **1a**/**1b**, **2b**/**2c** and **3a**/**3b**/**3c**. a) NaH, 4‐carbomethoxybenzyl bromide or PMBCl, DMF, RT, 1–2 h, 43–99 %; b) NH_2_OH (50 wt% in H_2_O), KCN or KOH, 1,4‐dioxane, RT, 24–48 h, 11–72 %; c) HNMe_2_, CH_2_O, HOAc, MeOH/H_2_O (5 : 1), 0 to 70° C, 48 h, 46 %; d) 4‐carbomethoxybenzyl bromide or PMBCl, K_2_CO_3_, DMF, RT, 12–16 h, 85 % (**9**), 74 % (**10**); e) HNMe_2_, NaBH(OAc)_3_, MeOH/H_2_O, 0 °C to RT, 48 h, 50 % (**11**), 39 % (**12**); f) H_2_SO_4_, 1,4‐dioxane, 60 °C, 20 h, 94 %; g) Boc_2_O, DMAP, CH_2_Cl_2_, RT, 15 min, 71 %; h) *t*‐BuLi, *N*‐formylmorpholine, THF, −100 °C, 10 min, 76 %; i) NaCN, MnO_2_, HOAc, MeOH, RT, 2 h, 73 %; j) TFA, CH_2_Cl_2_, 0 °C to RT, 2 h, 96 %; PMBCl, 4‐methoxybenzyl chloride; Boc_2_O, di‐*t*‐butyl dicarbonate; DMAP, 4‐dimethylaminopyridine.

The synthesis of N‐benzylated indole derivatives was performed in two to three steps starting with alkylation of indole building blocks **4**, **5**, or **8** with 4‐methoxybenzyl chloride (PMBCl) or 4‐carbomethoxybenzyl bromide to give **6**, **7**, **9**, and **10** (Scheme 1, top and middle). Indoles **1a** and **3a** were obtained by treatment of methyl esters **6** and **7** with hydroxylamine, respectively. Gramine derivative **1b** 24 was made from **1a** in a Mannich reaction by using formaldehyde and dimethylamine. Gramines **2b** and **3b** were obtained from formylindoles **9** and **10**, respectively, via reductive amination with dimethylamine to give **11** and **12**, followed by hydroxamic acid formation.^25^ The γ‐carboline scaffold of Tubastatin A analogues **2c** and **3c** was prepared by Fischer indole synthesis using hydrazine **13** and 1‐methylpiperidin‐4‐one (**14**), with subsequent BOC protection to give **15** (Scheme 1, bottom). Bromide **15** was converted to ester **16** via carbonylation, Corey−Gilman−Ganem oxidation, and then BOC removal.^26^ Lastly, alkylation of **16** with PMBCl or 4‐carbomethoxybenzyl bromide before hydroxamic acid formation provided **2c** and **3c**, respectively.

**Scheme 2:**
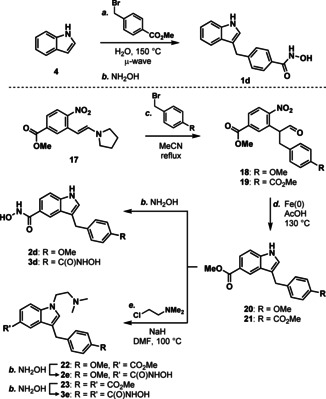
Synthesis of C3‐benzylated derivatives **1d**, **2d**/**2e** and **3d**/**3e**. a) 4‐carbomethoxybenzyl bromide, H_2_O, 150 °C, μ‐wave, 5 min, 45 %; b) NH_2_OH (50 wt% in H_2_O), KCN or KOH, 1,4‐dioxane, RT, 24 h, 15–51 %; c) 4‐methoxybenzyl bromide or 4‐carbomethoxybenzyl bromide, MeCN, 90 °C, 5 h, 78 % (**18**), 91 % (**19**); d) Fe powder, AcOH/PhMe (3 : 4), 130 °C, 4 h, 54 % (**20**), 44 % (**21**); e) 2‐chloroethyl‐*N*,*N*‐dimethylamine ⋅ HCl, NaH, DMF, 100 °C, 65 % (**22**), 17 % (**23**).

We used different approaches to access the C3‐benzylated indole derivatives. Selective C3‐alkylation of indole **4** under microwave irradiation in H_2_O,^27^ followed by hydroxamic acid formation gave **1d** (Scheme 2, top). Alternatively, the C3‐benzyl indole scaffold was constructed starting from enamine **17** (Scheme 2, bottom). Alkylation of **17** with 4‐methoxybenzyl bromide or 4‐carbomethoxybenzyl bromide afforded nitroaldehydes **18** and **19**, respectively, which, after Fe‐mediated reduction, directly cyclized to the C3‐substituted indoles **20** and **21**.^28^ These substances were either directly converted to hydroxamic acids **2d** and **3d**, or equipped with a dimethylaminoethyl group prior to installation of the hydroxamic acid ZBG to yield **2e** and **3e**.^29^, ^30^

**Scheme 3:**
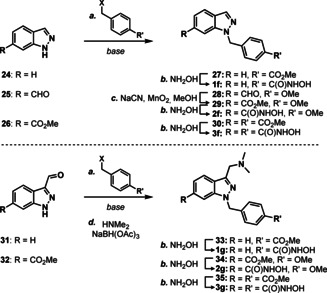
Synthesis of indazole derivatives **1f**/**1g**, **2f**/**2g** and **3f**/**3g**. a) *t*‐BuOK or K_2_CO_3_ or NaH, 4‐carbomethoxybenzyl bromide or 4‐methoxybenzyl chloride, DMF, RT, 1–18 h, 25–79 %; b) NH_2_OH (50 wt% in H_2_O), KCN or KOH, 1,4‐dioxane, RT, 24–48 h, 14–93 %; c) NaCN, MnO_2_, AcOH, MeOH, RT, 2 h, 95 %; d) HNMe_2_, NaBH(OAc)_3_, MeOH/H_2_O, 0 °C to RT, 48 h, 25–73 %.

We synthesized indazole derivatives **1f**, **2f** and **3f** in a similar fashion to the N‐benzylated indole derivatives by nucleophilic substitution of indazoles **24**–**26** with benzyl halides to give **27**, **28**, and **30** (Scheme 3, top). Ester **29** was obtained from **28** via Corey−Gilman−Ganem oxidation, and **27**, **29**, and **30** were converted to hydroxamic acids **1f**, **2f**, **3f**.

In analogy to the gramine derivatives **2b**/**3b**, an additional step for the synthesis of the hydroxamic acids **1g**, **2g** and **3g** was performed (Scheme 3, bottom). Starting from indazoles **31** and **32**,^31^ benzylation followed by reductive amination gave **33**–**35**, which were converted to the corresponding hydroxamic acids **1g**, **2g**, and **3g** as before.

## Results and Discussion

As our primary interest was to target HDAC8 and HDAC10, the substances were first tested against the two proteins in an enzymatic (HDAC‐Glo^TM^ I/II) and a ligand displacement FRET assay,^19^ respectively (Table 1). Starting with the set of compounds based on the simple PCI‐34051 scaffold (**1a**/**2a**/**3a**), we were pleased to see confirmation of our design concept: Monohydroxamic acid **1a** showed good activity against HDAC10 (pIC_50_=7.18) and moderate activity against HDAC8 (pIC_50_=6.42). As expected, PCI‐34051 (**2a**) was found to be highly selective for HDAC8 (pIC_50_=7.29) over HDAC10 (pIC_50_=4.38). Critically, dihydroxamic acid **3a** had good activity against HDAC8 (pIC_50_=7.17) and excellent HDAC10 (pIC_50_=8.59) activity. The other two non‐basic C‐3 benzyl (**1d**/**2d**/**3d**) and indazole (**1f**/**2f**/**3f**) scaffolds also showed internally consistent profiles.

**Table 1:** HDAC8 and HDAC10 inhibitory data.

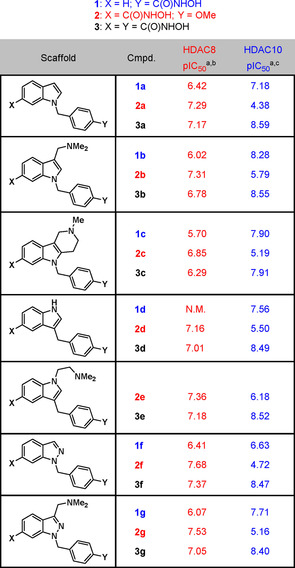

[a] See Table S1 in the Supporting Information for error values associated with these calculations. [b] pIC_50_ values determined with an enzymatic HDAC‐Glo^TM^ I/II assay. [c] pIC_50_ values determined with a ligand‐displacement FRET assay. N.M.=not measured.

Within these three scaffolds, inhibition of HDAC8 was slightly diminished by the introduction of a second hydroxamic acid moiety when compared to its parent monohydroxamic acid inhibitor (e. g. **3a** compared to **2a**). We were intrigued to find that HDAC10 activity was strongly increased by the addition of a second hydroxamic acid group in all three cases, with **3f** showing the largest increase in potency by a factor of ∼70 over **1f**. The high potency of these substances, despite their lack of a basic amine group, was surprising in light of our previous findings with Tubastatin A derivatives.^19^

We also examined the γ‐carboline series **1c**/**2c**/**3c** and found the HDAC10 activity of **3c** is similar to **1c** (Tubastatin A). The bulky γ‐carboline as a linker group (**2c**) produced the weakest HDAC8 inhibitor from the six monohydroxamic acids **2a**–**2g**. Furthermore, the corresponding dihydroxamic acid derivative **3c** showed even further diminished inhibition values toward HDAC8 (pIC_50_=6.29). This was consistent with our hypothesis that γ‐carboline derivatives would be too bulky.

We have previously shown that “ring‐opened” Tubastatin A derivative **1b** is a potent HDAC10 binder, with >100‐fold selectivity over HDACs 1, 2, and 3.^19^ Consequently, the gramine‐type derivatives **1b**/**2b**/**3b** and **1g**/**2g**/**3g** were investigated. Furthermore, the conceptually similar **3e** and its monohydroxamic acid analog **2e** were prepared.^29^ All three hybrids (**3b**/**3e**/**3g**) gave high HDAC10 pIC_50_ values of 8.55, 8.52, and 8.40, respectively. As with the other scaffolds, HDAC8 inhibition was detrimentally affected by the second hydroxamic acid, but compounds **3e** and **3g** showed acceptable profiles with HDAC8 pIC_50_ values of 7.18 and 7.05, respectively.

Having shown that our design plan succeeded with a variety of scaffolds, we tested the dihydroxamic acids (**3a**–**3g**) against all the remaining Class I and Class IIb HDACs to establish selectivity profiles (Table 2). HDACs 1, 2, 3, and 6 were assayed with the HDAC‐Glo^TM^ I/II system. As expected, all of the dihydroxamic acids are excellent HDAC6 inhibitors, with pIC_50_ values of 7.12–7.82. They also show some increased activity against HDAC1–3, when compared to PCI‐34051 and Tubastatin A. Compounds **3b** and **3c** have relatively weak HDAC8 and moderate HDAC1 activity, disqualifying them for further biological investigation along with **3a** and **3f**, which have the highest HDAC1–3 activities of all the inhibitors. Substances **3d**, **3e**, and **3g** have the best selectivity profiles overall with low HDAC2/HDAC3 and moderate HDAC1 activity, and were selected for further biological testing (vide infra).

**Table 2:** Data for hybrid inhibitors against Class I and Class IIb HDACs.

Cmpd	pIC_50_^a^					
	HDAC1^b^	HDAC2^b^	HDAC3^b^	HDAC6^b^	HDAC8^b,c^	HDAC10^c,d^
PCI‐34051 (**2a**)	4.33	<4.0	4.24	5.15	7.29	4.38
Tubastatin A (**1c**)	5.91	4.89	5.78	7.70	5.70	7.90
**3a**	6.58	5.65	5.26	7.74	7.17	8.59
**3b**	6.21	5.27	5.16	7.12	6.78	8.55
**3c**	6.30	5.38	4.87	7.32	6.29	7.91
**3d**	6.28	5.33	5.19	7.65	7.01	8.49
**3e**	6.09	5.15	5.18	7.35	7.18	8.52
**3f**	7.06	6.14	5.88	7.82	7.37	8.47
**3g**	6.18	5.02	5.18	7.52	7.05	8.40

[a] See Table S1 for error values associated with these calculations. [b] Enzymatic HDAC‐Glo^TM^ I/II assay. [c] Values taken from Table 1. [d] Ligand displacement FRET assay.

Parallel to our biochemical evaluation, we attempted to co‐crystallize the dual inhibitors with *Danio rerio* (zebrafish) HDAC10,^32^ where we had introduced A24E and D94A substitutions to more closely resemble the human HDAC10 active site. The crystal structure of the “humanized” zebrafish HDAC10‐**3a** complex was determined at 2.05 Å resolution (PDB 6VNQ), whereas other inhibitors either gave no crystals, or their resulting crystals diffracted poorly. The overall protein structure is quite similar to that of wild‐type zebrafish HDAC10 in its complex with a slender trifluoroketone inhibitor,^33^ with a root‐mean‐square deviation (rmsd) of 0.24 Å for 514 Cα atoms (Table S2). However, due to the rigidity and bulk of **3a** there are significant local structural changes in the active site. Specifically, the 3_10_ helix containing the P^23^(E,A)CE motif that protrudes into the active site shifts, on average, by 1.4 Å (maximum shift=1.9 Å). In other HDAC10 structures, the P^23^(E,A)CE motif sterically constricts the active site, presumably to favor the binding of long slender polyamine substrates. However, the current structure reveals that this motif can shift to accommodate the binding of certain bulky inhibitors.

**Figure 2:**
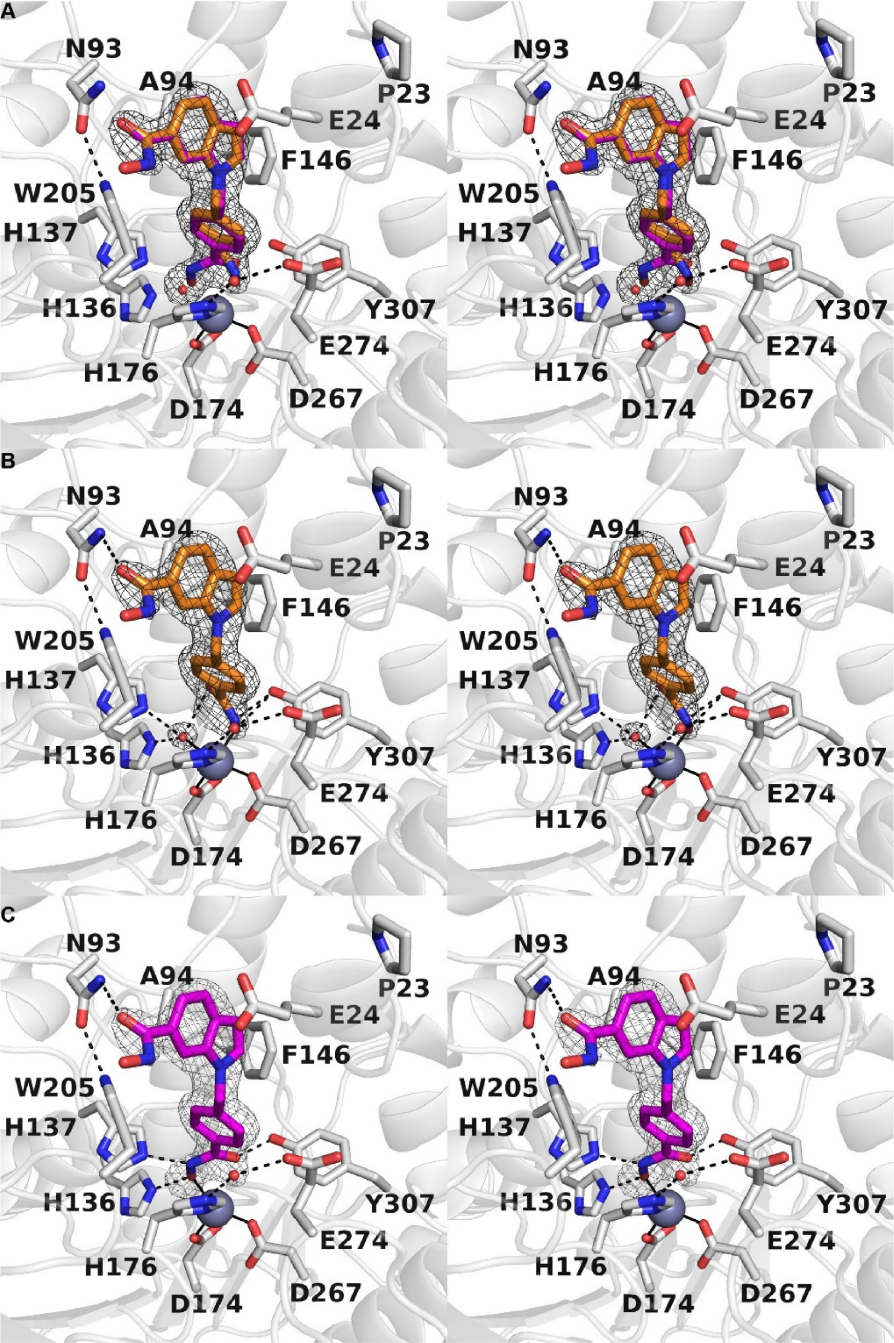
Stereoviews of Polder omit maps of **3a** in complex with HDAC10 (contoured at 6.0 *σ*, PDB 6VNQ). Atoms are color‐coded as follows: C=light gray (HDAC10), orange (major conformer of **3a**), or maroon (minor conformer of **3a**), N=blue, O=red, Zn^2+^=gray sphere, and solvent=small red spheres. Metal coordination interactions are shown as solid black lines, and hydrogen bonds are shown as dashed black lines. A) Both conformers in complex with HDAC10. B) Major conformer in complex with HDAC10. C) Minor conformer in complex with HDAC10.

Zinc coordination by the ionized hydroxamate group of **3a** is achieved by a mixture of two different monodentate binding modes (Figure 2A). The hydroxamate of the major conformer (67 % occupancy) coordinates to zinc through the N−O^−^ group (O−Zn^2+^ separation=2.1 Å; Figure 2B). The phenolic hydroxyl group of Y307 is within hydrogen bonding distance to both the hydroxamate NH and N−O^−^ groups (O−N and O−O separations=2.6 and 2.7 Å, respectively). A Zn^2+^‐bound water molecule is also observed (O−Zn^2+^ separation=2.2 Å), which donates a hydrogen bond to the hydroxamate C=O group (O−O separation=3.1 Å) and forms hydrogen bonds with H136 and H137 (O−N separations=2.3 and 2.7 Å, respectively).

At first glance, the hydroxamate group of the minor conformer of **3a** (33 % occupancy) appears to coordinate to Zn^2+^ in a manner similar to that observed for bidentate hydroxamate‐zinc interactions as observed in other HDAC10‐inhibitor complexes (Figure 2C).^33^ However, the C=O−Zn^2+^ separation of 2.7 Å is not consistent with inner sphere metal coordination. Thus, inner sphere Zn^2+^ coordination is achieved solely by the hydroxamate N−O^−^group (O−Zn^2+^ separation=2.4 Å). The Zn^2+^‐bound N−O^−^ group accepts a hydrogen bond from H136 (O−N separation=2.6 Å) and the hydroxamate NH group donates a hydrogen bond to H137 (N−N separation=2.5 Å). Additionally, Y307 forms an anomalously short hydrogen bond (2.2 Å) with the hydroxamate C=O group. The phenyl ring of **3a** makes favorable offset π stacking interactions in an aromatic crevice defined by F146 and W205, as well as the Zn^2+^ binding residue H176. Interestingly, the side chain of E24 packs against the indole ring of the inhibitor. The A24E substitution was made to “humanize” the active site of zebrafish HDAC10, so the packing interaction between E24 and the indole ring of **3a** is presumably important for binding to human HDAC10. The indole hydroxamate is not fully defined by electron density, potentially due to disorder, but it clearly forms a hydrogen bond with N93, which in turn forms a hydrogen bond with W205. Of note, N93 of zebrafish HDAC10 aligns with D91 of human HDAC10, so these hydrogen bond interactions may be somewhat altered upon inhibitor binding to human HDAC10.

**Figure 3:**
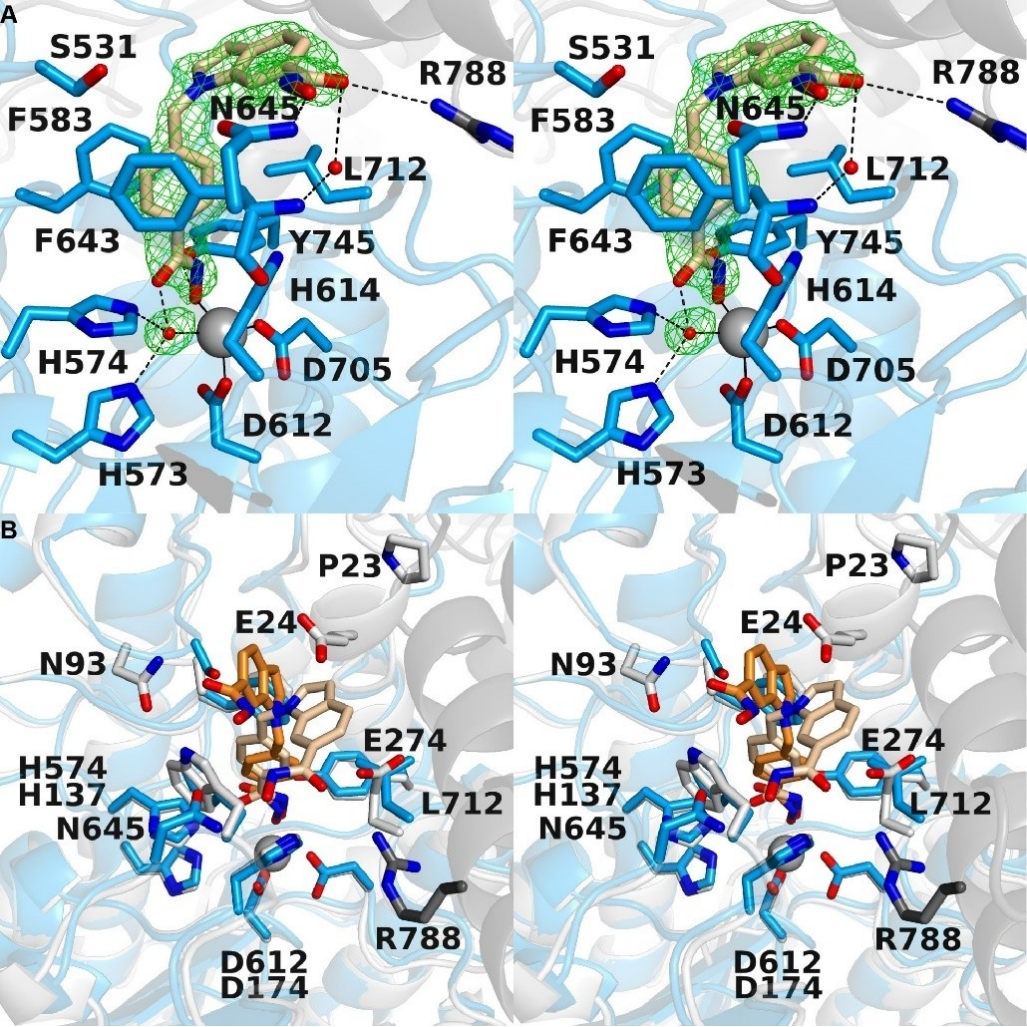
A) Stereoview of the Polder omit maps of the HDAC6‐**3a** complex (monomer A, contoured at 3.5 *σ*, PDB 6VNR). Atoms are color‐coded as follows: C=light blue (HDAC6), dark gray (symmetry mate), or wheat (inhibitor), N=blue, O=red, Zn^2+^=gray sphere, and solvent=small red spheres. Metal coordination and hydrogen bond interactions are indicated by solid and dashed black lines, respectively. B) Stereoview of a superposition of the complexes of **3a** with HDAC6 and HDAC10. Atoms are color‐coded as follows: C=light blue (HDAC6) or white (HDAC10), dark gray (symmetry mate of HDAC6), wheat (**3a** bound to HDAC6), orange (**3a** bound to HDAC10), N=blue, O=red, and Zn^2+^=gray sphere.

Although no dihydroxamic acid in this study provided crystals of sufficient quality with HDAC8, the crystal structure of **3a** complexed with *D. rerio* HDAC6 was determined at 1.94 Å resolution (Table S3, PDB 6VNR). The structure of this complex reveals that **3a** binds to HDAC6 as a single conformer (Figure 3A). The catalytic Zn^2+^ ion is coordinated in monodentate fashion by the hydroxamate N−O^−^ group of **3a** (average O−Zn^2+^ separation=2.1 Å), and the hydroxamate C=O group accepts a hydrogen bond from the Zn^2+^‐bound water molecule (average O−O separation=2.4 Å). The Zn^2+^ coordination geometry is similar to that observed for the major conformer of **3a** bound to HDAC10 (Figure 2B). Also similar to the HDAC10‐**3a** complex, the aromatic ring of the phenylhydroxamate nestles in an aromatic crevice, here defined by F583 and F643.

Interestingly, superposition of the two enzyme‐inhibitor complexes reveals that the capping group conformation of **3a** differs between the HDAC6 and HDAC10 complexes (Figure 3B). As observed in the HDAC10‐**3a** complex, the capping group is clamped down by E24 of the P^23^(E,A)CE motif; however, the capping group of **3a** in the HDAC6 complex is oriented toward solution; the indole hydroxamate makes hydrogen bond interactions with N645 as well as R788 of a symmetry mate in the crystal lattice. Since the inhibitor capping group has a specific binding site in a pocket in the active site of HDAC10 that is not conserved in the active site of HDAC6, this feature must contribute to selectivity for binding to HDAC10.

**Figure 4:**
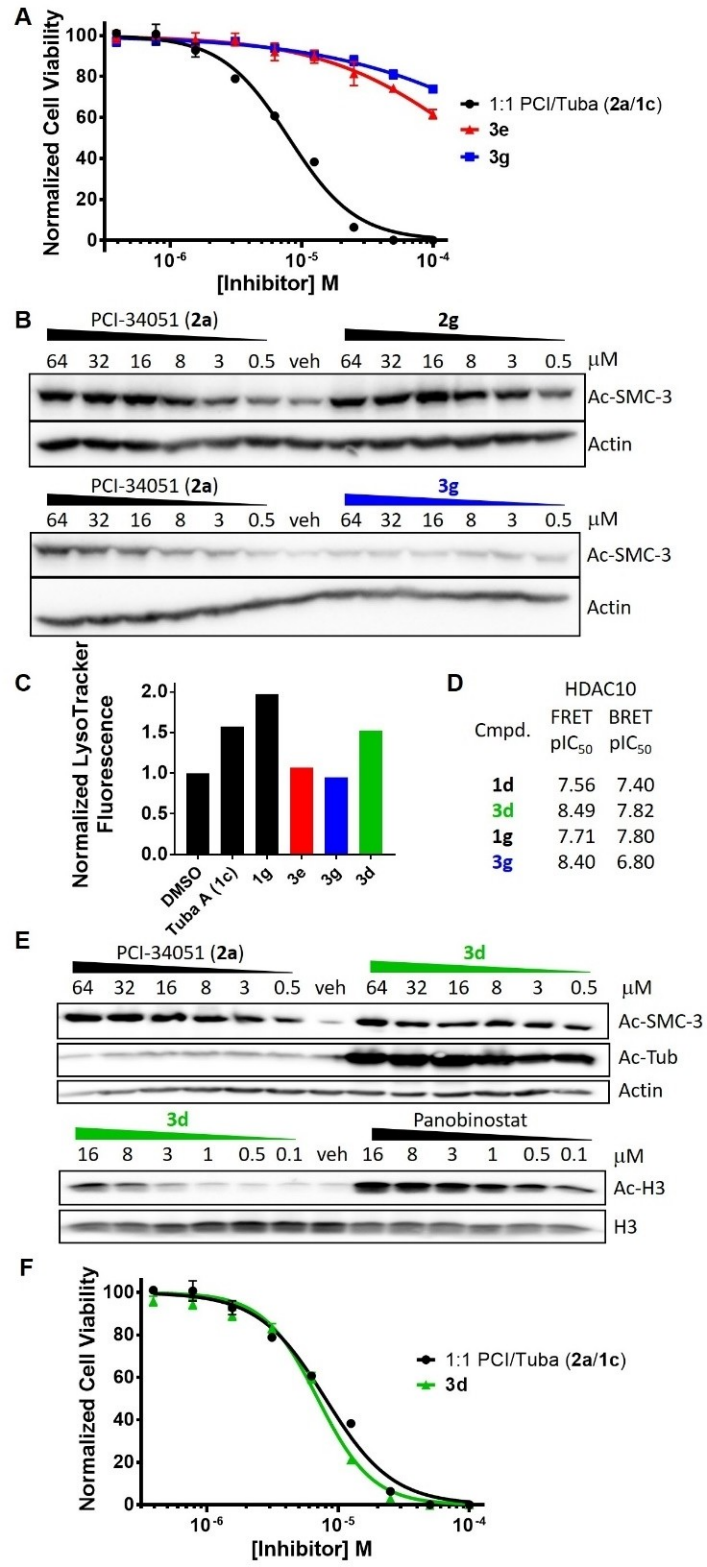
A) Dose‐response data for hybrid inhibitors and a 1 : 1 molar ratio of PCI‐34051 (**2a**) and Tubastatin A (**1c**) in SK‐N‐BE(2)C cells. B) Western blots of Ac‐SMC‐3 with actin as a loading control (SK‐N‐BE(2)C). C) Fluorescence readout from SK‐N‐BE(2)C cells treated with 10 μM of each inhibitor, normalized to cells treated with DMSO. D) Comparison of FRET and BRET HDAC10 binding data. FRET values are taken from Table 1. BRET values are determined from an experiment run in triplicate. E) Western blots of Ac‐SMC‐3, Ac‐tubulin, and Ac‐histone 3. The pan‐HDACi panobinostat was used as a positive control for histone 3 acetylation. F) Dose‐response data for **3d** and a 1 : 1 molar ratio of PCI‐34051 (**2a**) and Tubastatin A (**1c**). The cell proliferation assays were run in triplicate. Error bars represent S.D. Data are normalized to a vehicle control, which was also performed in triplicate.

We next measured viability of the HDAC8/10 sensitive SK‐N‐BE(2)C neuroblastoma cell line^6^ after treatment with our hybrid inhibitors. On the basis of our biochemical profiling and previous experience with Tubastatin A derivatives,^19^ we began with the two amine‐containing inhibitors **3e** and **3g**. We were surprised to find that both hybrid molecules showed little effect up to 100 μM, although a 1 : 1 molar ratio of PCI‐34051 (**2a**) and Tubastatin A (**1c**) gave an IC_50_ value of 7.3 μM (6.5–8.1 μM 95 % C.I.; Figures 4A and S1). In order to explain this discrepancy, we measured cellular markers/phenotypes which are indicative of target engagement. Whereas PCI‐34051 (**2a**) and HDAC8 inhibitor **2g** increased acetylation of the HDAC8 substrate SMC3 in a dose‐dependent manner,^6^ hybrid inhibitor **3g** showed no effect relative to solvent control (Figure 4B). Furthermore, dihydroxamates **3e** and **3g** failed to produce an HDAC10 knockdown phenotype, i. e. increased lysosomal staining with the acidotropic LysoTracker DND‐99 dye (Figure 4C).8b Tubastatin A (**1c**) and monohydroxamate **1g** were effective as positive controls.

These data pointed toward poor cell permeability of the highly polar dihydroxamic acids **3e** and **3g** (Table S4). We examined this further by testing target engagement of selected inhibitors in a cellular BRET HDAC10 target engagement assay.^34^ Substances **1d** and **1g** had very similar FRET and BRET pIC_50_ values, indicating good cell permeability for monohydroxamic acids with or without a basic amine side chain (Figure 4D). Dihydroxamic acid **3g**, on the other hand, had a pIC_50_ value in the BRET assay which is more than 60 fold weaker than in the FRET assay, consistent with poor cell permeability. Compound **3d**, which lacks a basic amino side chain and is slightly less polar, showed only a moderate ∼5‐fold loss of potency between the FRET and BRET assays, pointing toward improved cell permeability.

This was supported by the fact that **3d** induces lysosomal acidification similar to Tubastatin A (**1c**; Figure 4C). In light of this finding, we tested **3d** in cells and found it increases acetylation of SMC3 (HDAC8 substrate) and tubulin (HDAC6 substrate), while having significantly weaker effects on histone H3 (HDAC1 substrate; Figure 4E). Pleasingly, **3d** alone, was as effective (IC_50_=7.0 μM; 6.5–7.5 μM 95 % C.I.) as combination treatment with Tubastatin A (**1c**) and PCI‐34051 (**2a**) in a SK‐N‐BE(2)C cell viability assay (Figure 4F).

As discussed above, previous data from our group showed that only those Tubastatin A derivatives which contain a basic nitrogen in the cap group are potent (pIC_50_≥∼8.0) HDAC10 binders.^19^ At the outset of this study, we assumed that our hybrid molecules would also require a basic nitrogen in their cap/linker group. Even though all the compounds in this study which contain such a nitrogen are indeed potent HDAC10 binders (i. e. **3b**, **3c**, **3e**, and **3g**), we were surprised to find that this functionality was not required (i. e. **3a**, **3d**, and **3f**). In the latter case, the addition of a hydroxamic acid at C6 of the indole ring was sufficient to produce highly potent HDAC10 binders (e. g. **3f** versus **1f**). The hydrogen bond found between the cap group hydroxamic acid in **3a** with N93 in zebrafish HDAC10 may also be formed with D91 in human HDAC10, potentially explaining the tight HDAC10 binding of the dihydroxamic acids. Hydrogen bonding to D91 may offer another opportunity, in addition to binding with the gatekeeper E272 residue, for the development of potent and selective HDAC10 binding.

## Conclusion

In summary, the similar central scaffolds of PCI‐34051 (**2a**) and Tubastatin A (**1c**) served as the basis to develop proof of concept hybrid molecules which target HDAC8 and HDAC10 (and HDAC6). We synthesized potent and selective inhibitors and gained structural insight into the binding of “bulky” inhibitors to HDAC10, but not all substances showed cellular activity. We attribute this discrepancy to poor cell permeability, potentially due to the high polarity of the substances. Dihydroxamic acid **3d** is sufficiently cell permeable to inhibit the growth of SK‐N‐BE(2)C cells, and showed activity similar to a combination treatment of **2a** and **1c**. Future work could aim to improve efficiency of such hybrids using a pro‐drug strategy that masked one or both of the hydroxamic acid groups, or via the replacement of at least one of the hydroxamic acids with a different ZBG.

## Experimental Section

**Expression and purification of TwinStrepII‐GST‐HDAC10**: A synthetic gene encoding TwinStrepII‐GST‐HDAC10 (human) was ordered from GeneArt (Thermo Fischer Scientific) and subcloned into the pFastBac1 vector. The resulting construct was used for transposition in *E. coli* DH10EMBacY cells. The isolated bacmid DNA was then used to generate the recombinant baculovirus. For protein expression, 10 mL of baculovirus was added to 1 L of Sf21 cells at a density of 1×10^6^ cells/mL. The infected Sf21 cells were grown for 72 h in Sf‐900 III SFM medium (Thermo Fischer Scientific) at 27 °C. Cells were harvested by centrifugation and re‐suspended in running buffer (100 mM Tris pH 8.0, 150 mM NaCl, 1 mM EDTA and 1 mM DTT) supplemented with 10 mM MgCl_2_, benzonase and cOmplete protease inhibitors (Merck). The cells were lysed using a Dounce homogenizer and the resulting lysate was centrifuged for 30 min at 4 °C at 125 000 *g* in an ultracentrifuge. The clarified lysate was then loaded onto a 5 mL Strep‐Tactin Superflow high capacity column (IBA) pre‐equilibrated in running buffer. After sample loading and washing, the TwinStrepII‐GST‐HDAC10 protein was eluted in running buffer supplemented with 5 mM desthiobiotin (IBA). The elution fractions containing TwinStrepII‐GST‐HDAC10 were pooled and concentrated before being injected onto a HiLoad 16/600 Superdex 200 pg size exclusion chromatography column (GE Healthcare) pre‐equilibrated with 25 mM HEPES/NaCl pH 7.5, 150 mM NaCl, 0.5 mM EDTA, 1 mM DTT and 10 % glycerol. Samples were eluted from the size exclusion chromatography column in the same buffer, flash‐frozen in liquid N_2_ and stored at −80 °C.

**Note on the TR‐FRET assay**: We have made slight modifications to the TR‐FRET assay since the original publication where we described it.^19^ Control experiments indicate that pIC_50_ values for a given inhibitor tested in both assay formats are not statistically different. Therefore, data from the two assay formats can be reliably compared. The TR‐FRET measurements of the monohydroxamic acids in this manuscript were measured in the original assay format, which is described directly below this paragraph. The TR‐FRET measurements of the dihydroxamic acids in this manuscript were measured in the modified format, which is described two paragraphs below this one.

**TR‐FRET assay (used with the monohydroxamic acids)**: All TR‐FRET experiments were performed in white 384‐well ProxiPlates (PerkinElmer) using 50 mM HEPES pH 8.0, 150 mM NaCl, 10 mM MgCl_2_, 1 mM EGTA and 0.01 % Brij‐35 (+2 % DMSO) as buffer. The concentrations of reagents in 10 μL final assay volume were 3 nM GST‐HDAC10, 30 nM “Tubastatin‐Alexa647‐Tracer” and 0.5 nM Eu‐anti‐GST. GST‐HDAC10 and LanthaScreen™ Eu‐anti‐GST Antibody were purchased from Life Technologies, and the Tubastatin‐Alexa647‐Tracer was synthesized in‐house as previously described.^19^ An 11‐fold 1 : 3‐serial dilution of compounds starting at 2 mM was prepared in 384 well pp‐plates (Greiner) from 10 mM stocks in DMSO and 1 μL was transferred to assay plates. Nine μL of the reagent‐mix was added and the plate was incubated for 1 h at RT before TR‐FRET was measured in an EnVision™ plate reader equipped with a TR‐FRET Laser module. Sample wells were exited with 3 flashes of the TRF‐Europium Laser, and emission was measured at 620 nm and 665 nm to get the 665 nm/620 nm ratio. Percent inhibition was calculated for each well from negative control wells containing 2 % DMSO and positive control wells containing 20 μM SAHA. The resulting dose‐response curves were fitted in ActivityBase (IDBS) using a four‐parameter logistic model and pIC_50_‐values were calculated.

**TR‐FRET assay (used with dihydroxamic acids)**: TR‐FRET assays were performed in white 384‐well plates (4512, Corning) using 50 mM HEPES pH 8.0, 150 mM NaCl, 10 mM MgCl_2_, 1 mM EGTA and 0.01 % Brij‐35 as buffer. The concentrations of reagent in 15 μL final assay volume were 5 nM TwinStrep‐GST‐HDAC10 (preparation described above), 25 nM “Tubastatin‐AF647‐Tracer” (**S15**, synthesis in Supporting Information) and 0.1 nM DTBTA‐Eu^3+^‐labeled Streptactin (synthesis in the Supporting Information). Inhibitors were tested at eight serial dilutions in triplicates ranging from 50 μM–86.7 pM and dosed from 10 mM and 0.1 mM DMSO stock solutions with a D300e Digital Dispenser (Tecan). After drug dosing to the premixed assay reagents in buffer, plates were shaken (800 rpm orbital shaker, 30 s), centrifuged (300 *g*, 1 min) and incubated at room temperature in the dark for 60 min. TR‐FRET was measured with a CLARIOstar (BMG Labtech) plate reader, equipped with TR‐FRET filters. Sample wells were excited with 100 flashes and fluorescence emission detected at 665 nm and 620 nm. FRET ratios were calculated from 665 nm/620 nm ratio and normalized for each plate using 50 μM SAHA treated negative controls and uninhibited positive controls. pIC_50_‐values were calculated using nonlinear regression log(inhibitor) four parameters least squares fit in GraphPad Prism version 7.04 for Windows (GraphPad Software, La Jolla, CA, USA, www.graphpad.com).

**Production of mono‐clones stably expressing HDAC‐nanoBRET proteins**: Plasmids expressing a fusion of HDAC10 with nanoluciferase were obtained from Promega (N2170). HeLa cells (0.75×10^6^) were seeded in a 6 cm dish and after 24 h were transfected with a mix of 10 μg plasmid and 3 μL Fugene in 200 μL OptiMEM. In detail, cells were washed with pre‐warmed OptiMEM and subsequently overlaid with 2.3 mL of OptiMEM. After addition of 200 μL transfection mix, cells were incubated for 24 h at 37 °C. Cells were than trypsinized and 0.2×10^5^ cells were seeded into both 10 cm and 15 cm dishes. Transformants were selected with 1 mg/mL G‐418 for 6 days with a media change after 3 days. Clones which formed colonies were picked by rinsing plates with 3 mL Trypsin/EDTA (Sigma T3924) followed by a 2 min incubation with 300 μL Trypsin/EDTA at 37 °C. Colonies were then loosened and aspirated with a 10 μL filter tip and transferred to 24‐well plates containing selection medium. Clones exhibiting a range of nanoluciferase activities were expanded and selected according to the highest BRET ratio.

**Culture of stable BRET cell lines**: Stably transfected HeLa cells were cultivated under sterile conditions in polystyrene cell culture flasks (658170, Greiner) at 37 °C and 5 % CO_2_ in a humidified atmosphere. DMEM growth medium (D6049, Sigma) was supplemented with 10 % FCS (FBS‐12A, Capricorn Scientific), 1 % penicillin‐streptomycin (P4333, Sigma) and 1 mg/mL Geneticin (2039.3, Roth). At confluency, cells were passaged by removing old medium, DPBS (14190‐094, gibco) wash, trypsination (T4049, Sigma) and seeding in fresh growth medium.

**BRET assay**: The intracellular target engagement assay on HDAC10 was performed as described by the kit manufacturer in a 96‐well plate (3600, Corning) format with 1.9×10^4^ cells per well and a tracer concentration of 0.3 μM. Inhibitors were tested at ten 1 : 4 serial dilutions in triplicates ranging from 129 pM to 40 μM. Drug dosing was performed from 10 mM and 1 mM DMSO stock solutions with a D300e Digital Dispenser (Tecan), DMSO concentrations were normalized to 0.5 % for all wells. Assay plates were incubated at 37 °C for 2 h followed by measurement of 450 nm and 650 nm luminescence (80 nm bandwidth) at room temperature with a CLARIOstar (BMG Labtech) plate reader 2 min after NanoLuc substrate addition.

BRET ratios were calculated from 650 nm/450 nm luminescence and normalized for each plate using 50 μM SAHA treated negative controls and uninhibited positive controls. pIC_50_‐values were calculated from normalized BRET ratios using nonlinear regression log(inhibitor) four parameters least squares fit in GraphPad Prism version 7.04 for Windows.

**HDAC‐Glo assay for HDAC 1, 2, 3, 6 and 8**: HDAC6 and class I inhibition was tested using the HDAC‐Glo™ I/II assay and Screening System (G6421, Promega) with recombinant human HDACs (BPS Bioscience; HDAC1 cat. #50051; HDAC2 cat. #50002; HDAC3/NcoR2 complex cat. #50003; HDAC6 cat. #50006; HDAC8 cat. #50008). The assay was carried out in a 384‐well plate (4512, Corning) format according to the manufacturer's description. Inhibitors were tested at eight serial dilutions in triplicate ranging from 50 μM–86,7 pM (HDAC6) or 100 μM–8,67 nM (HDAC1,2,3,8). Drug dosing was performed from 10 mM and 0.1 mM DMSO stock solutions with a D300e Digital Dispenser (Tecan). HDACs (7 ng/mL for HDAC1, 10 ng/mL for HDAC2, 200 ng/mL for HDAC3/Ncor2 complex, 100 ng/mL for HDAC6, 200 ng/mL for HDAC8) and inhibitors were incubated together for 30 min at room temperature. After addition of the HDAC‐Glo™ I/II reagent, plates were shaken (800 rpm orbital shaker, 30 s), centrifuged (300 *g*, 1 min) and incubated at room temperature for 30 min. Luminescence was detected with a CLARIOstar (BMG Labtech) plate reader. Luminescence signal was normalized with 100 μM SAHA treated negative controls and uninhibited positive controls. pIC_50_‐values were calculated as described in the BRET assay.

**Western blot analysis of SMC3, tubulin and histone H3 acetylation**: For the analysis of protein acetylation, 1.5–2×10^6^ SK‐N‐BE(2)‐C cells were seeded per 10 cm dish and allowed to adhere overnight. Cells were treated for 18 h (Figure 4B) or 6 h (Figure 4E) with inhibitors at concentrations indicated in the respective figure. Cells were lysed in SDS lysis buffer (2 % *w*/*v* SDS, 10 % *v*/*v* glycerol, 1 mM DTT, 62.5 mM TRIS, pH 6.8) and proteins were denatured at 95 °C for 10 min. Cell debris was removed by centrifugation (15 000 *g* for 10 min at 12 °C). Western blot analysis was performed as described before.7a The following antibodies were used: anti‐acetylated tubulin (6‐11B‐1; Sigma) anti‐acetylated SMC3 (kindly provided by Katsuhiko Shirahige, Institute for Molecular and Cellular Biosciences, University of Tokyo, Japan),^35^ anti acetylated histone H3 (#06‐911, Millipore), anti histone H3 (#9715, Cell Signaling Technology) and anti β‐actin (#5441, Sigma‐Aldrich).

**LysoTracker assay**: SK‐N‐BE(2)‐C cells were seeded into 6‐well dishes at a density of 1.5×10^5^ cells per well. Cells were treated with inhibitor over night at concentrations indicated in the figure and stained the following day for 1 h with 50 nM LysoTracker® Red DND‐99 in medium under standard cell culture conditions. Cells were washed with ice‐cold RPMI without phenol‐red and trypsinized for 3 min at 37 °C. Detached cells were centrifuged for 3 min at 8600 *g* and re‐suspended in ice‐cold RPMI without phenol red. Mean LysoTracker fluorescence was quantified on a BD FACSCanto II platform using the PE filter setting. Data were normalized to mean LysoTracker fluorescence of solvent (DMSO) treated cells.

**Cell viability assay**: SK‐N‐BE(2)C cells were cultured in Dulbecco's modified Eagle medium (DMEM, Lonza, Basel, Switzerland) supplemented with 10 % fetal calf serum (FCS, Sigma‐Aldrich) and 1 % non‐essential amino acids (NEAA, Lonza). SK‐N‐BE(2)C cells were seeded from a confluent flask into 96‐well plates in 100 μL full growth medium at a density of 5×10^3^ cells per well the day before treatment. Cells were treated in triplicates with drug concentrations as indicated in the respective figure. Drugs were dosed from 50 mM and 200 mM stock solutions with a D300e Digital Dispenser (Tecan) and DMSO concentrations were normalized to 0.2 % for all wells. Plates were shaken (1000 rpm orbital shaker, 30 s) and incubated under standard culture conditions for 72 h. To each well 20 μL of CellTiter‐Blue® reagent (G8081, Promega) was added, plates were shaken (600 rpm orbital shaker, 20 s) and incubated at 37 °C overnight. Fluorescence was measured (extinction/emission: 570 nm/590 nm) on a FluoStar Optima (BMG Labtech) plate reader. Cell viability was normalized with untreated positive controls and cell‐free negative controls. IC_50_‐values were calculated as described in the BRET assay.

**Expression, purification, and crystallization of HDAC10**. A “humanized” version of HDAC10 was designed by making the A24E and D94A substitutions in *D. rerio* HDAC10 so as to more closely resemble the active site of human HDAC10. The preparation of this HDAC10 construct using standard PCR mutagenesis techniques will be described separately; purification was achieved as described for the wild‐type enzyme.^32^, ^33^ For crystallization, the protein solution [10 mg/mL HDAC10, 50 mM HEPES pH 7.5, 300 mM KCl, 5 % glycerol (*v*/*v*), and 1 mM tris‐(2‐carboxyethyl)phosphine (TCEP) was augmented with 2 mM **3a** and incubated for 1 h on ice. Trypsin was added (1 : 1000 trypsin:HDAC10) and the mixture was allowed to digest at ambient temperature for 1 h and then filtered using a 0.22 μm centrifuge filter. Utilizing a Mosquito crystallization robot (TTP Labtech), a 100 nL drop of protein solution was added to a 100 nL drop of precipitant solution [0.168 M KH_2_PO_4_, 0.032 M K_2_HPO_4_, and 20 % PEG 3350] and microseeded with crystals of the HDAC10‐Tubastatin A complex. The 200 nL sitting drop was equilibrated against 80 μL of precipitant buffer in the well reservoir at 4 °C. Crystals appeared within one day.

**Crystal structure determination of the HDAC10‐3a complex**: X‐ray diffraction data for the HDAC10‐**3a** complex were collected on NE‐CAT beamline 24‐ID‐C at the Advanced Photon Source (APS), Argonne National Laboratory. Data were indexed by using iMosflm^36^ and scaled with Aimless^37^ in the CCP4 program suite.^38^ The initial electron density map was phased by molecular replacement using Phaser;^39^ the structure of Y307F HDAC10 (PDB 5TD7)^32^ with solvent and ligand atoms removed was used as a search model. An iterative process of model building using Coot^40^ and crystallographic refinement with Phenix^41^ yielded the final model of the HDAC10‐**3a** complex. The inhibitor was built into the electron density map during the final stages of refinement. MolProbity^42^ was used to validate the final refined structure, which was deposited in the Protein Data Bank (PDB 6VNQ). All data reduction and refinement statistics are recorded in Table S2.

**Expression, purification, and crystallization of HDAC6**. HDAC6 catalytic domain 2 (CD2) from *D. rerio* was used for the X‐ray crystal structure determination of the complex with **3a**. The active sites of human and zebrafish HDAC6 CD2 are essentially identical, with the exception of N530 and N645 at the mouth of the active site of zebrafish HDAC6 CD2, which appear as aspartate and methionine, respectively, in the human enzyme.^43^ The expression and purification of zebrafish HDAC6 CD2 (henceforth simply “HDAC6”) was achieved by modification of the originally reported preparation as recently described.^44^ For co‐crystallization of the HDAC6‐**3a** complex by the sitting drop method at 4 °C using a Mosquito crystallization robot (TTP Labtech), a 500 nL drop of protein solution [10 mg/mL HDAC6, 50 mM HEPES (pH 7.5), 100 mM KCl, 5 % glycerol (*v*/*v*), 1 mM TCEP, and 2 mM **3a**] was added to a 500 nL drop of precipitant solution [0.02 M citrate/0.08 M Bis‐Tris propane (pH 8.8) and 24 % *w*/*v* PEG 3350] and equilibrated against 80 μL of precipitant solution in the well reservoir. Crystals appeared within 2 days.

**Crystal structure determination of the HDAC6‐3a complex**. X‐ray diffraction data were collected on NE‐CAT beamline 24‐ID‐E at APS. Data indexing was achieved with iMosflm^36^ and Aimless^37^ was used for data scaling, as implemented in the program suite CCP4.^38^ The initial electron density map of the enzyme‐inhibitor complex was phased by molecular replacement using Phaser^39^ with the atomic coordinates of unliganded HDAC6 CD2 (PDB ID: 5EEM)^43^ used as a search model. The interactive graphics program Coot^40^ was used to build and manipulate the atomic model, and Phenix^41^ was used for crystallographic refinement. The inhibitor was built into the electron density map during the final stages of refinement. MolProbity^42^ was used to validate the final structure prior to deposition in the Protein Data Bank (PDB 6VNR). All data reduction and refinement statistics are recorded in Table S3.

## Author Contributions

M.M., M.G., and A.K.M. designed and synthesized compounds. R.S., P.S., and L.H. performed the HDAC enzymatic and ligand displacement assays. L.H. performed the cell viability assays. J.R. performed the Western Blot and Lysotracker assays. I.O, O.W., and N.G. oversaw biological experiments. C.J.H.‐G., J.D.O., N.J.P., and D.W.C. performed the crystallography experiments. M.M., D.W.C., and A.K.M. wrote the manuscript. A.K.M. designed and coordinated the project. All authors have given approval to the final version of the manuscript.

## Conflict of interest

The authors declare no competing financial interest.

## Supporting information

As a service to our authors and readers, this journal provides supporting information supplied by the authors. Such materials are peer reviewed and may be re‐organized for online delivery, but are not copy‐edited or typeset. Technical support issues arising from supporting information (other than missing files) should be addressed to the authors.

Supplementary
