# Supplementary material for: Design and Synthesis of Dihydroxamic Acids as HDAC6/8/10 Inhibitors
Source: ChemMedChem. 2020 May 13;15(13):1163–74. doi: 10.1002/cmdc.202000149 (PMC7335359; doi:10.1002/cmdc.202000149)
Supplement: Supplementary file 1 — Supplementary [file CMDC-15-1163-s001.pdf]

ChemMedChem

Supporting Information

Design and Synthesis of Dihydroxamic Acids as HDAC6/8/10 Inhibitors

Michael Morgen, Raphael R. Steimbach, Magalie Géraldy, Lars Hellweg, Peter Sehr, Johannes Ridinger, Olaf Witt, Ina Oehme, Corey J. Herbst-Gervasoni, Jeremy D. Osko, Nicholas J. Porter, David W. Christianson, Nikolas Gunkel, and Aubry K. Miller*© 2020 The Authors. Published by Wiley-VCH Verlag GmbH & Co. KGaA.
This is an open access article under the terms of the Creative Commons Attribution License, which permits use, distribution and reproduction in any medium, provided the original work is properly cited.

Table of Contents

Chemical Synthesis 2

Figure S1. Dose-response curves of Tubastatin A, PCI-34051, and their combination 31

Table S1. Enzyme inhibition values with error calculations 32

Table S2. Crystallographic data collection and refinement statistics for the HDAC10-3a complex 33

Table S3. Crystallographic data collection and refinement statistics for the HDAC6-3a complex 34

Table S4. Prediction of log P values of synthesized inhibitors 35

References 36

NMR Spectra 37

Chemical Synthesis

General: Starting materials, reagents, and solvents were purchased from commercial sources (Sigma-Aldrich Chemie GmbH, Taufkirchen, Germany; ABCR GmbH & Co. KG, Karlsruhe, Germany and Merck KGaA, Darmstadt, Germany) at the highest level of purity and used without purification, or prepared according to published procedures, which are specifically referenced. The reference compounds **1c** (Tubastatin A) and **2a** (PCI-34051) were obtained from Sigma-Aldrich. Anhydrous dichloromethane, toluene, acetonitrile and tetrahydrofuran were prepared with an MBraun SPS800 Solvent Purification System. Thin layer chromatography (TLC) was carried out on Merck glass silica plates. TLC visualization was accomplished using 254 or 366 nm UV light, iodine saturated silica gel (I_2), or charring solutions of potassium permanganate ($KMnO_4$), cerric ammonium molybdate (CAM), or *p*-anisaldehyde (PAA). Purification was performed by flash column chromatography using SiliCycle SiliaFlash® P60 (40–63 µm, 60 Å particle size) or via RP-HPLC (Agilent 1260 Infinity and an ES quadrupole Agilent 6120, column: Kinetex® 5µm C18 100 Å, AXIA Packed LC Column 250 x 21.2 mm; Temperature = 40 °C; Solvent A = H_2O, 0.05% TFA; Solvent B = MeCN, 0.05% TFA; Flow Rate = 15.0 mL/min; method: Gradient: 5% B to 30% B [over 4 min], then 30% B to 60% B [over 8 min], then 0% B to 95% B [over 4 min]). Basic column: Gemini® 5µm C18 110 Å, AXIA Packed LC Column 250 x 21.2 mm; Temperature =room temperature; Solvent A = H_2O, 0.1% NH_3; Solvent B = MeCN; Flow Rate = 15.0 mL/min.

Analytical LC/MS was performed on an Agilent 1260 Infinity system using reverse phase. Column: Kinetex® 2.6 µm C18 100 Å, LC Column 50 x 2.1 mm; Temperature = 40 °C; Solvent A = H_2O, 0.01% HCO_2H; Solvent B = MeCN, 0.01% HCO_2H; Flow Rate = 0.60 mL/min; method: Gradient: 1% B to 90% B [over 6 min] then 90% B to 99% B [over 2 min]). High resolution mass spectrometry was recorded on a Bruker ApexQe FT-ICR instrument, (Department of Organic Chemistry, University of Heidelberg). NMR spectra were recorded on Bruker 400 MHz or 600 MHz instruments at 298.1 K. Melting points were determined on a semi-automated apparatus (MPM-H2, Coesfeld Materialtest). All final compounds were found to have ≥ 95% purity, controlled by analytical (HPLC/UV/ELSD/MS) with the above described method, and confirmed by ^{1}H NMR.

Compound **6**

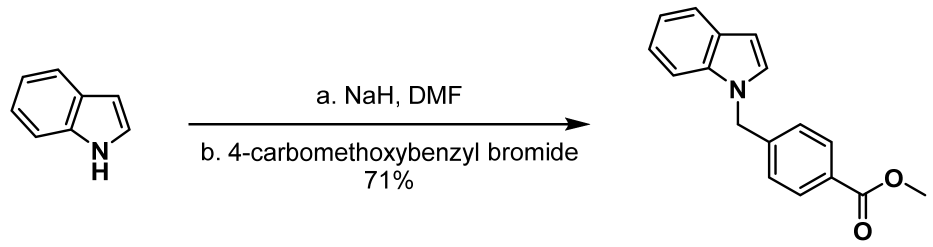

4 **6**

methyl 4-((1H-indol-1-yl)methyl)benzoate (**6**): To a suspension of sodium hydride (60% in oil, 1.7993 g, 74.98 mmol) in dimethylformamide (20 mL) was added 1H-indole (**4**) (3.06 g, 26.13 mmol). This suspension was stirred at room temperature for 2 h. 4-carbomethoxybenzyl bromide (8.93 g, 38.99 mmol) was added and the reaction mixture was stirred for 20 h at room temperature. The excess of base was consumed through the addition of a solution of saturated ammonium chloride (100 mL). The reaction mixture was extracted with ethyl acetate (3 x 50 mL). The organic phase was washed with brine, dried over magnesium sulfate, filtered and concentrated *in vacuo*. Purification by flash chromatography (*n*-hexane/EtOAc 90/10) afforded the desired ester **6** (4.90 g, 18.4 mmol, 71%) as a light yellow solid.

R$_F$ 0.52 (*n*-hexane/EtOAc 80/20).
^{1}**H NMR** (400 MHz, $CDCl_3$) δ 8.00 - 7.98 (m, 2H), 7.73 - 7.70 (m, 1H), 7.26 - 7.21 (m, 2H), 7.19 - 7.14 (m, 4H), 6.63 (dd, *J* = 3.2, 0.8 Hz, 1H), 5.37 (s, 2H), 3.92 (s, 3H) ppm.
^{13}**C NMR** (101 MHz, $CDCl_3$) δ 166.8, 142.8, 136.3, 130.1, 129.6, 128.8, 128.3, 126.6, 122.0, 121.2, 119.8, 109.6, 102.2, 52.2, 49.9 ppm.
LC-MS *m/z*: $[M + H]^+$ 266.

Compound **1a**

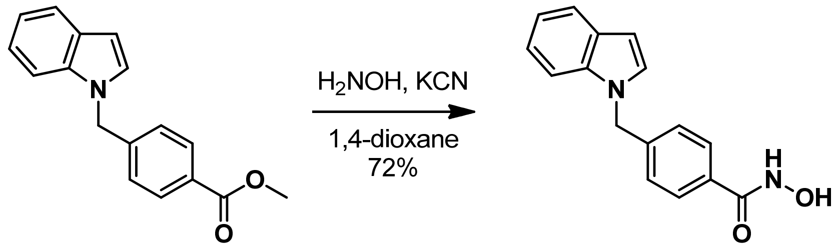

6 **1a**

4-((1H-indol-1-yl)methyl)-*N*-hydroxybenzamide (**1a**): To a solution of **6** (4.90 g, 18.48 mmol) in 1,4-dioxane (20 mL) were added an aqueous hydroxylamine solution (50%, 15 mL) and potassium cyanide (508 mg, 7.800 mmol). The reaction mixture was stirred for 48 h at room temperature. The reaction was stopped with the addition of a solution of saturated sodium hydrogen carbonate (50 mL) and the mixture was extracted with ethyl acetate (2 x 25 mL). The organic phase was washed with brine, dried over magnesium sulfate, filtered and concentrated *in vacuo*. The desired hydroxamic acid **1a** (3.57 g, 13.40 mmol, 72%) was obtained as an off-white solid without further purification.

R$_F$ 0.71 (CH_2Cl_2:MeOH, 90:10).
^{1}**H NMR** (400 MHz, DMSO-d_6) δ 11.09 (brs, 1H), 9.14 (brs, 1H), 7.70 - 7.68 (m, 2H), 7.57 (d, *J* = 7.8 Hz, 1H), 7.51 (d, *J* = 3.1 Hz, 1H), 7.42 (dd, *J* = 8.2, 0.6 Hz, 1H), 7.24 - 7.22 (m, 2H), 7.11 - 7.07

(ddd, J = 8.1, 7.0, 1.1 Hz, 1H), 7.02 (ddd, J = 7.8, 7.0, 1.0 Hz, 1H), 6.51 (dd, J = 3.1, 0.7 Hz, 1H), 5.47 (s, 2H) ppm.
^{13}C NMR (100 MHz, DMSO-d_6) δ 163.9, 141.4, 135.7, 131.9, 129.2, 128.4, 127.1, 126.9, 121.3, 120.6, 119.2, 110.1, 101.2, 66.4, 48.8 ppm.
HRMS-ESI *m/z*: $[M+Na]^+$ calcd for $C_{16}H_{14}N_2O_2Na$: 289.0947; found: 289.0952.
mp 148.5 °C (decomposition).

Compound **1b**

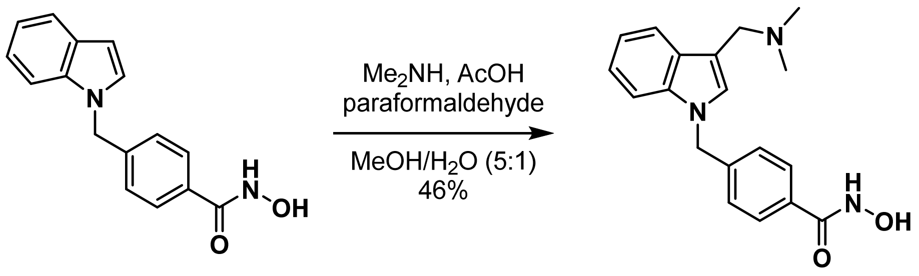

1a 1b

4-((3-((dimethylamino)methyl)-1H-indol-1-yl)methyl)-N-hydroxybenzamide (**1b**): To a solution of dimethylamine (66.0 µL, 34.60 g, 130 µmol) and acetic acid (30 µL, 33.0 mg, 549 µmol) in methanol (5 mL) and water (1 mL) was added paraformaldehyde (12.9 mg, 0.41 mmol) and **1a** (34.6 mg, 0.13 mmol) at 0 °C. The resulting reaction mixture was heated up to 70 °C for 48 h. The reaction mixture was cooled to room temperature and concentrated *in vacuo*. Purification by HPLC afforded the trifluoroacetate salt of the desired hydroxamic acid **1b** (26.2 mg, 0.060 mmol, 46%) as a light pink oil.

R_F 0.31 (CH_2Cl_2/MeOH 80/20).
^{1}H NMR (400 MHz, DMSO-d_6) δ 11.17 (s, 1H), 9.65 (s, 1H), 7.81 (d, J = 7.4 Hz, 1H), 7.74 (s, 1H), 7.69-7.67 (m, 2H), 7.50 (d, J = 7.7 Hz, 1H), 7.27-7.25 (m, 2H), 7.20-7.12 (m, 2H), 5.53 (s, 2H), 4.47 (d, J = 4.5 Hz, 2H), 2.76 (d, J = 4.8 Hz, 6H) ppm.
^{13}C NMR (101 MHz, $CDCl_3$) δ 163.8, 140.8, 135.9, 132.1, 132.0, 127.9, 127.2, 127.0, 122.2, 120.1, 119.1, 110.7, 103.2, 51.0, 48.9, 41.3 ppm.
HRMS-ESI *m/z*: $[M–H]^-$ calcd for $C_{19}H_{20}N_3O_2$: 322.1561; found: 322.1560.

Compound **7**

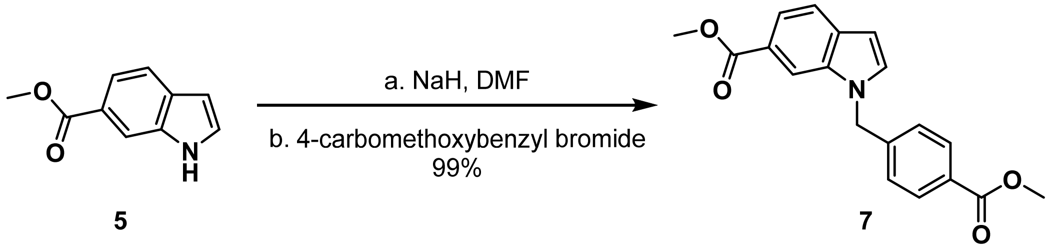

5 7

methyl 1-(4-(methoxycarbonyl)benzyl)-1H-indole-6-carboxylate (**7**): To a suspension of sodium hydride (60% in oil, 53.4 mg, 1.34 mmol) in dimethylformamide (6 mL) was added methyl 1H-indole-6-carboxylate (**5**) (175.7 mg, 1.00 mmol). This suspension was stirred at room temperature for 1 hour. 4-carbomethoxybenzyl bromide (260.0 mg, 1.14 mmol) was added and the mixture was stirred for 15 minutes at room temperature. The excess of base was consumed through the addition of a solution of

saturated ammonium chloride (10 mL). The reaction mixture was extracted with ethyl acetate (3 x 5 mL). The organic phase was washed with brine, dried over magnesium sulfate, filtered and concentrated *in vacuo*. Purification by flash chromatography (*n*-hexane/EtOAc 80/20 to 70/30) afforded the desired ester **7** (320.0 mg, 0.99 mmol, 99%) as a yellow oil.

R$_F$ 0.27 (*n*-hexane/EtOAc 80/20).
^{1}H NMR (400 MHz, $CDCl_3$) δ 8.03 (brs, 1H), 7.98 - 7.96 (m, 2H), 7.82 (dd, *J* = 8.4, 1.4 Hz, 1H), 7.67 (dd, *J* = 8.4, 0.6 Hz, 1H), 7.28 (d, *J* = 3.1 Hz, 1H), 7.15 - 7.13 (m, 2H), 6.32 (dd, *J* = 3.2, 0.9 Hz, 1H), 5.45 (s, 2H), 3.90 (s, 3H), 3.89 (s, 3H) ppm.
LC-MS *m/z*: $[M+Na]^+$ 346.

Compound **3a**

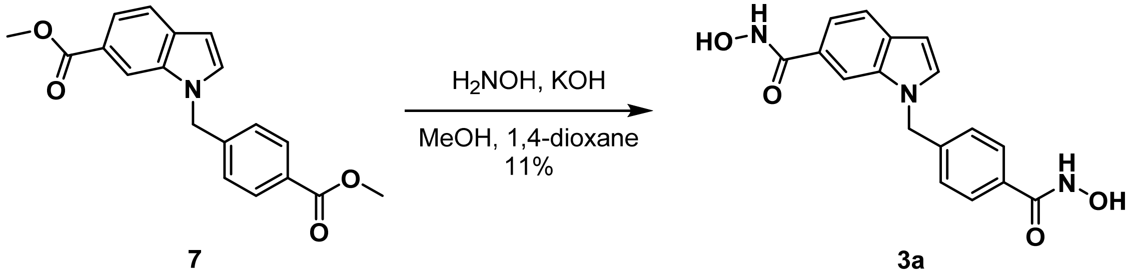

7

3a

***N*-hydroxy-1-(4-(hydroxycarbamoyl)benzyl)-1H-indole-6-carboxamide** (**3a**): To a solution of **7** (100.0 mg, 0.31 mmol) in methanol (4 mL) and 1,4-dioxane (2 mL) were added an aqueous solution hydroxylamine (50 wt%, 4 mL) and potassium hydroxide (70.0 mg, 1.25 mmol). The reaction mixture was stirred for 48 h at room temperature. The reaction was stopped with the addition of a solution of saturated ammonium chloride (10 mL) and the mixture was extracted with ethyl acetate (5 x 5 mL). The organic phase was washed with brine, dried over magnesium sulfate, filtered and concentrated *in vacuo*. The resulting solid was triturated with cold acetonitrile, filtered, washed with acetonitrile:diethyl ether (50:50), filtered and dried. The desired hydroxamic acid **3a** (11 mg, 0.034 mmol, 11%) was obtained as a white solid.

^{1}H NMR (400 MHz, DMSO-d_6) δ 11.13 (br. s, 1H),11.08 (br. s, 1H), 8.99 (brs, 1H), 8.88 (brs, 1H), 7.90 (s, 1H), 7.68 - 7.67 (m, 3H), 7.59 (d, *J* = 8.4 Hz, 1H), 7.45 (d, *J* = 8.1 Hz, 1H), 7.24 - 7.22 (m, 2H), 6.56 (s, 1H), 5.52 (s, 2H) ppm.
LC-MS *m/z*: $[M+H]^+$ 326.

Compound **9**

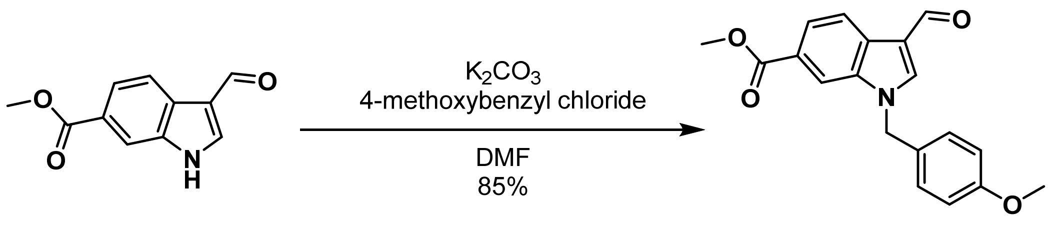

8 **9**

methyl 3-formyl-1-(4-methoxybenzyl)-1H-indole-6-carboxylate (**9**)**:** To a solution of methyl 3-formyl-1H-indole-6-carboxylate (**8**) (2.32 g, 11.42 mmol) and 4-methoxybenzyl chloride (1.95 g, 1.55 mL, 11.42 mmol) in dimethylformamide (20 mL) was added potassium carbonate (4.74 g, 34.26 mmol). The suspension was stirred for 16 h at room temperature. The mixture was diluted with water (100 mL) and extracted with ethyl acetate (3 x 100 mL). The combined organic phases were washed with water (2 x 100 mL) and brine (100 mL), dried over $MgSO_4$, filtered and concentrated. The desired ester **9** (3.14 g, 9.7 mmol, 85%) was obtained as a white solid.

^{1}H NMR (400 MHz, $CDCl_3$) δ 10.00 (s, 1H), 8.34 (dd, *J* = 8.4, 0.7 Hz, 1H), 8.17 (dd, *J* = 1.4, 0.7 Hz, 1H), 8.00 (dd, *J* = 8.4, 1.4 Hz, 1H), 7.77 (s, 1H), 7.21 – 7.14 (m, 2H), 6.94 – 6.87 (m, 2H), 5.34 (s, 2H), 3.95 (s, 3H), 3.81 (s, 3H) ppm.

Compound **10**

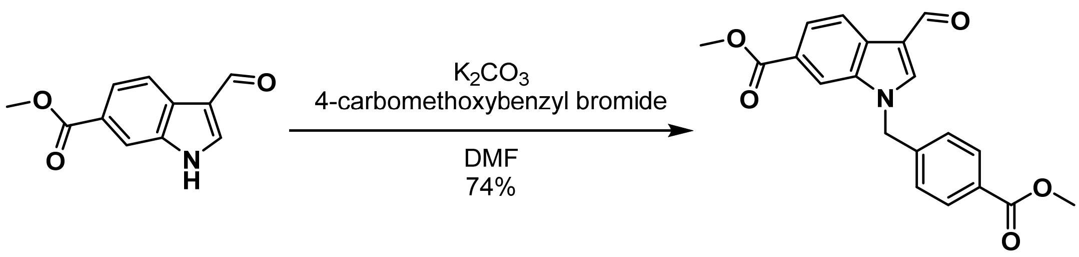

8 **10**

methyl 3-formyl-1-(4-(methoxycarbonyl)benzyl)-1H-indole-6-carboxylate (**10**): To a solution of methyl 3-formyl-1H-indole-6-carboxylate (**8**) (2.5017 g, 12.31 mmol) and 4-carbomethoxybenzyl bromide (3.4180 g, 14.92 mmol) in dimethyl formamide (20 mL) was added potassium carbonate (3.0736 g, 22.24 mmol). The suspension was stirred for 16 h at room temperature. The mixture was diluted with water (50 mL). The solid which precipitated was filtered out and was recrystallized in ethyl acetate. The crystals were filtered and washed with ethyl acetate. The desired ester **10** (3.22 g, 9.16 mmol, 74%) was collected as a light yellow solid.

R_F 0.50 (*n*-hexane/EtOAc 50/50).
^{1}H NMR (400 MHz, DMSO-d_6) δ 10.01 (s, 1H), 8.67 (s, 1H), 8.23 (d, *J* = 8.3 Hz, 1H), 8.14 (s, 1H), 7.94-7.92 (m, 2H), 7.87 (dd, *J* = 8.3, 1.4 Hz, 1H), 7.37 - 7.35 (m, 2H), 5.78 (s, 2H), 3.84 (s, 3H), 3.81 (s, 3H) ppm.
LC-MS *m/z*: $[M+H]^+$ 352.

Compound **11**

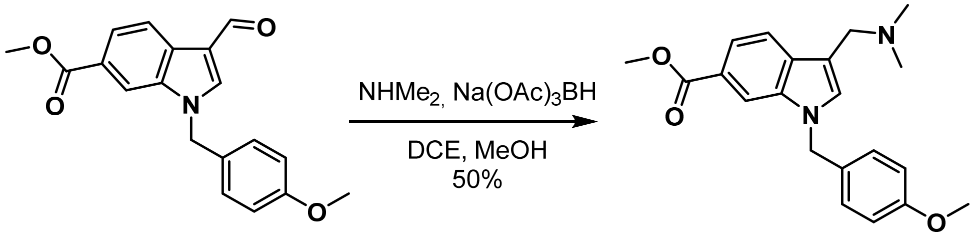

9 **11**

methyl 3-((dimethylamino)methyl)-1-(4-methoxybenzyl)-1H-indole-6-carboxylate (**11**)[1]: To a solution of **9** (470.0 mg, 1.45 mmol) in 20.0 mL of dichloroethane (DCE) and dimethylamine (2.0 M in MeOH, 2.18 mL, 4.35 mmol) was added sodium triacetoxyborohydride (614.6 mg, 2.90 mmol) at 0 °C. Upon complete addition the cooling bath was removed and the reaction mixture was stirred for 48 h at room temperature. The reaction was stopped with the addition of dichloromethane (10 mL) and a solution of saturated sodium hydrogen carbonate (20 mL). The aqueous and organic layers were separated and the organic phase was washed with brine, dried over magnesium sulfate, filtered and concentrated *in vacuo*. Purification by flash chromatography (CH_2Cl_2/MeOH 90/10) afforded the desired product **11** (254.5 mg, 0.72 mmol, 50%) as a white solid.

^{1}H NMR (400 MHz, DMSO-*d_6*) δ 8.08 – 8.05 (m, 1H), 7.71 (dd, *J* = 8.4, 0.7 Hz, 1H), 7.63 (dd, *J* = 8.4, 1.4 Hz, 1H), 7.60 (s, 1H), 7.16 – 7.09 (m, 2H), 6.90 – 6.85 (m, 2H), 5.41 (s, 2H), 3.83 (s, 3H), 3.69 (s, 3H), 3.56 (s, 2H), 2.16 (s, 6H) ppm.

Compound **12**

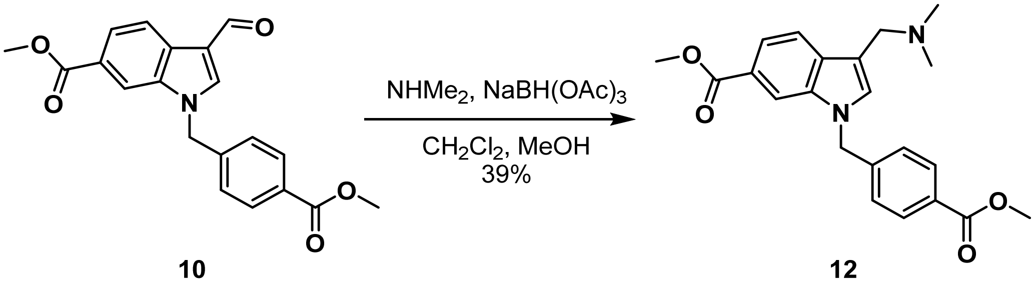

methyl 3-((dimethylamino)methyl)-1-(4-(methoxycarbonyl)benzyl)-1H-indole-6-carboxylate (**12**)[1]: To a solution of **10** (481.3 mg, 1.37 mmol) in CH_2Cl_2 (20 mL) and dimethylamine (2.0 M in MeOH, 1.30 mL, 2.6 mmol) was added $NaBH(OAc)_3$ (790.2 mg, 3.73 mmol) at 0 °C. Upon complete addition the cooling bath was removed and the reaction mixture was stirred for 48 h at room temperature. The reaction was stopped with the addition of CH_2Cl_2 (10 mL) and a solution of saturated $NaHCO_3$ (20 mL). The aqueous and organic layers were separated and the organic phase was washed with brine, dried over $MgSO_4$, filtered and concentrated *in vacuo*. Purification by flash chromatography (CH_2Cl_2/MeOH 90/10) afforded **12** (205.7 mg, 0.64 mmol, 39%) as a light yellow solid.

R_F 0.16 (CH_2Cl_2/MeOH 90/10).
^{1}H NMR (400 MHz, DMSO-*d_6*) δ 8.02 (m, 1H), 7.91 - 7.89 (m, 2H), 7.77 (dd, *J* = 8.4, 0.5 Hz, 1H), 7.69 (s, 1H), 7.66 (dd, *J* = 8.4, 1.4 Hz, 1H), 7.25 - 7.23 (m, 2H), 5.63 (s, 2H), 3.81 (d, *J* = 0.8 Hz, 6H), 3.70 (s, 2H), 2.24 (s, 6H) ppm.
LC-MS *m/z*: $[M - (NMe_2)]^+$ 336.

Compound **2b**

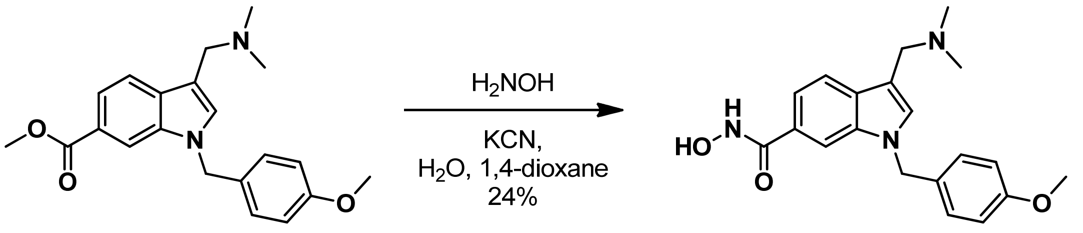

11 **2b**

3-((dimethylamino)methyl)-*N*-hydroxy-1-(4-methoxybenzyl)-1H-indole-6-carboxamide (**2b**): To a solution of **11** (103.8 mg, 0.29 mmol) in a mixture of 1,4-dioxane (3.0 mL)/methanol (0.5 mL) were added an aqueous solution of hydroxylamine (50 wt%, 2.0 mL) and potassium cyanide (15.0 mg, 0.23 mmol). The reaction mixture was stirred at room temperature for 18 h and then concentrated on a rotary evaporator. The crude material was subjected to column chromatography (CH_2Cl_2/MeOH 90/10) to afford the pure product **2b** as a pale-red solid (25.7 mg, 0.07 mmol, 24%).

R$_F$ 0.1 (CH_2Cl_2/MeOH 90/10).
^{1}H NMR (400 MHz, DMSO-d_6) δ 11.08 (s, 1H), 8.90 (s, 1H), 7.93 – 7.89 (m, 1H), 7.64 (d, J = 8.4 Hz, 1H), 7.51 (s, 1H), 7.44 (dd, J = 8.3, 1.4 Hz, 1H), 7.20 – 7.14 (m, 2H), 6.90 – 6.85 (m, 2H), 5.34 (s, 2H), 3.69 (s, 3H), 3.57 (s, 2H), 2.16 (s, 6H) ppm.
^{13}C NMR (101 MHz, DMSO-d_6) δ 165.3, 158.6, 135.4, 130.7, 130.2, 129.9, 128.4, 125.7, 119.0, 117.5, 114.0, 111.4, 109.3, 55.0, 53.8, 48.5, 44.7 ppm.
HRMS-ESI *m/z*: $[M - (Me_2N)]^+$ calcd for $C_{18}H_{17}N_2O_3$: 309.1234; found: 309.1235.

Compound **3b**

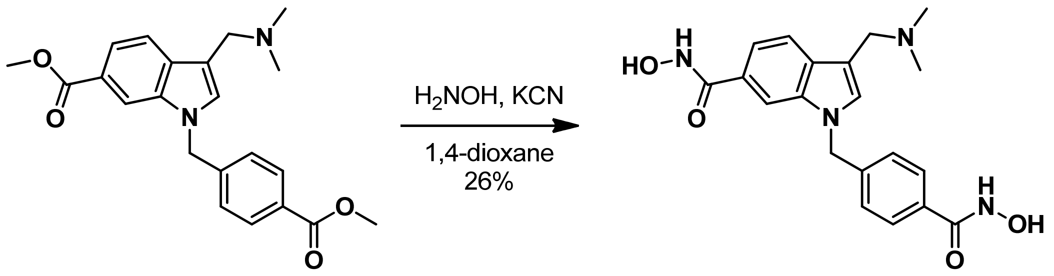

12 **3b**

3-((dimethylamino)methyl)-*N*-hydroxy-1-(4-(hydroxycarbamoyl)benzyl)-1H-indole-6-carboxamide (**3b**): To a solution of **12** (194.7 mg, 511.8 μmol) in 1,4-dioxane (10 mL) were added an aqueous solution of hydroxylamine (50 wt%, 6.0 mL) and potassium cyanide (43.0 mg, 0.66 mmol). The reaction mixture was stirred for 48 h at room temperature. The reaction was stopped with the removal of the excess of reagent and solvent in *vacuo*. Purification by HPLC afforded the desired hydroxamic acid **3a** as its trifluoroacetic acid salt (65.0 mg, 0.13 mmol, 26%) as a white solid.

R$_F$ 0.07 (CH_2Cl_2/MeOH 70/30).
^{1}H NMR (400 MHz, DMSO-d_6) δ 11.17 (s, 2H), 9.82 (br. s, 1H), 9.17 – 8.75 (br. s, 2H), 7.97 (d, J = 1.5 Hz, 1H), 7.89 – 7.84 (m, 2H), 7.73 – 7.66 (m, 2H), 7.57 (dd, J = 8.4, 1.4 Hz, 1H), 7.29 – 7.24 (m, 2H), 5.58 (s, 2H), 4.48 (s, 2H), 2.76 (s, 6H) ppm.

^{13}**C NMR** (101 MHz, DMSO-*d*$_6$) δ 164.7, 163.8, 158.0 (q, *J* = 30.9 Hz), 140.6, 135.4, 134.4, 132.1, 129.9, 127.3, 126.9, 126.7, 118.9, 118.8, 118.7, 112.8 (q, *J* = 595.9 Hz), 112.8, 103.5, 50.7, 49.0, 41.3 ppm.
HRMS-ESI *m/z*: $[M+H]^+$ calcd for $C_{20}H_{23}N_4O_4$: 383.1714; found: 383.1720.
mp 57.6 °C.

Compound **S1**

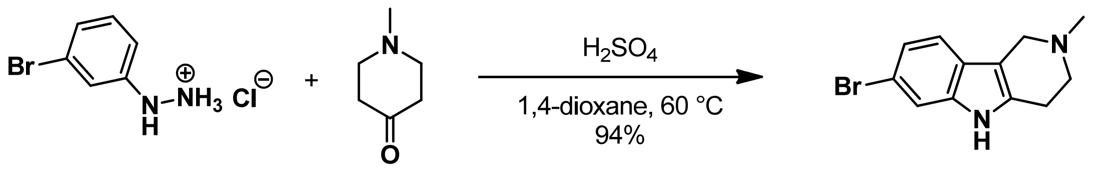

13 **14** **S1**

7-bromo-2-methyl-2,3,4,5-tetrahydro-1H-pyrido[4,3-b]indole (**S1**): To a suspension of 3-bromophenylhydrazine hydrochloride (**13**) (5.04 g, 22.55 mmol) in 1,4-dioxane (60 mL) was added 1-methylpiperidin-4-one (**14**) (2.77 mL, 2.55 g, 22.55 mmol) at 60 °C. Sulfuric acid (10.0 mL, 18.4 g, 180.12 mmol) was added dropwise and stirring was continued at 60 °C for 20 h. Afterwards, the reaction mixture was poured in an ice cold aqueous solution of sodium hydroxide (2 M, 50 mL), diluted with water (100 mL), and the mixture was extracted with ethyl acetate (2 x 50 mL). The organic phase was washed with brine (150 mL), dried over magnesium sulfate, filtered and concentrated *in vacuo*. The desired product **S1** (5.64 g, 21.25 mmol, 94%) was obtained as a white solid without further purification.

$\mathbf{R_F}$ 0.27 (CH_2Cl_2/MeOH 80/20).
^{1}**H NMR** (400 MHz, DMSO-*d*$_6$) δ 10.96 (s, 1H), 7.43 (dd, *J* = 0.4, 1.8 Hz, 1H), 7.26 (d, *J* = 8.4 Hz, 1H), 7.05 (dd, *J* = 1.8, 8.3 Hz, 1H), 3.48 (s, 2H), 2.80 – 2.75 (m, 2H), 2.70 - 2.67 (m, 2H), 2.41 (s, 3H) ppm.
LC-MS *m/z*: $[M+H]^+$ 265 & 267.

Compound **15**

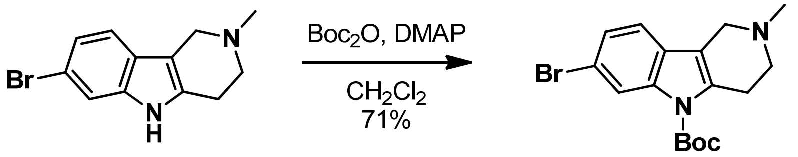

S1 **15**

***tert*-butyl 7-bromo-2-methyl-3,4-dihydro-1H-pyrido[4,3-b]indole-5(2H)-carboxylate** (**15**): To a stirred solution of **S1** (2.20 g, 8.30 mmol) in dichloromethane (100 mL) was added di-*tert*-butyl-dicarbonate (2.55 g, 11.69 mmol) and dimethylaminopyridine (73.4 mg, 0.60 mmol). The mixture was stirred for 15 minutes. The reaction was stopped with the removal of the excess of reagent and solvent in *vacuo*. Purification by flash chromatography (CH_2Cl_2/MeOH 94/6) afforded the desired product **15** (2.16 g, 5.913 mmol, 71%) as a yellow solid.

$\mathbf{R_F}$ 0.79 (CH_2Cl_2/MeOH 80/20).
^{1}**H NMR** (400 MHz, DMSO-*d*$_6$) δ 8.07 (s, 1H), 7.19 (br. s, 2H), 3.27 (s, 2H), 2.86 – 2.77 (m, 2H), 2.55 – 2.43 (m, 2H), 2.23 (br. s, 3H), 1.45 (s, 9H) ppm.
LC-MS *m/z:* $[M+H]^+$ 365 & 367.

Compound **S2**

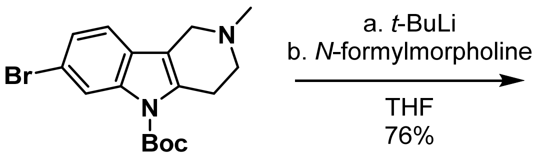

15 S2

***tert*-butyl 7-formyl-2-methyl-3,4-dihydro-1H-pyrido[4,3-b]indole-5(2H)-carboxylate** (**S2**): To a solution of **15** (2.791 g, 7.64 mmol) in tetrahydrofuran (153 mL) at –100 °C was added a solution of *tert*-butyllithium (1.7 M in pentane, 4.75 mL, 8.07 mmol). After stirring for 5 min, *N*-formylmorpholine (0.87 mL, 8.7 mmol) was added and the reaction mixture was stirred for 10 min. The reaction was stopped with the addition of a solution of saturated sodium hydrogen carbonate (30 mL) and the mixture was extracted with ethyl acetate (2 x 15 mL). The organic phase was washed with brine, dried over magnesium sulfate, filtered and concentrated *in vacuo*. Purification by flash chromatography (CH_2Cl_2/MeOH 96/4 to 94/6) afforded the desired indole **S2** (1.824 g, 5.80 mmol, 76%) as an orange solid.

R_F 0.53 (CH_2Cl_2/MeOH 90/10).
^{1}H NMR (400 MHz, $CDCl_3$) δ 10.04 (s, 1H), 8.66 (d, *J* = 1.0 Hz, 1H), 7.77 (dd, *J* = 8.1, 1.3 Hz, 1H), 7.43 (d, *J* = 8.1 Hz, 1H), 3.61 (t, *J* = 1.9 Hz, 2H), 3.20 - 3.17 (m, 2H), 2.81 (t, *J* = 5.8 Hz, 2H), 2.56 (s, 3H), 1.69 (s, 9H) ppm.
^{13}C NMR (101 MHz, $CDCl_3$) δ 192.5, 150.1, 138.3, 135.9, 132.9, 132.6, 123.4, 119.1, 117.8, 115.5, 84.7, 52.7, 51.3, 45.8, 28.4, 27.3 ppm.
LC-MS *m/z*: $[M+H]^+$ 315.

Compound **S3**

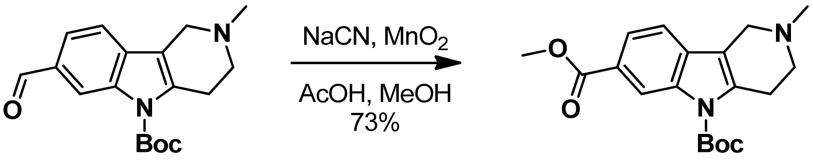

S2 S3

5-*tert*-butyl 7-methyl 2-methyl-3,4-dihydro-1H-pyrido[4,3-b]indole-5,7(2H)-dicarboxylate (**S3**): To a suspension of **S2** (1.824 g, 5.80 mmol), sodium cyanide (1.43 g, 29.24 mmol) and acetic acid (0.83 mL, 14.51 mmol) in methanol (120 mL) was added manganese dioxide (10.08 g, 115.9 mmol). The reaction mixture was stirred for 2 h at room temperature. The reaction was stopped with the removal of the excess of reagent and solvent *in vacuo*. The crude product was taken up in dichloromethane and the solids were filtered out. The filtrate was concentrated *in vacuo*. Purification by flash chromatography (CH_2Cl_2/MeOH 96/4 to 94/6) afforded the desired indole **S3** (1.46 g, 4.24 mmol, 73%) as an orange solid.

R_F 0.49 (CH_2Cl_2/MeOH 90/10).
^{1}H NMR (400 MHz, $CDCl_3$) δ 8.86 - 8.85 (m, 1H), 7.91 (dd, *J* = 8.2, 1.5 Hz, 1H), 7.34 (d, *J* = 8.2 Hz, 1H), 3.92 (s, 3H), 3.58 (t, *J* = 1.9 Hz, 2H), 3.19 - 3.16 (m, 2H), 2.78 (t, *J* = 5.8 Hz, 2H), 2.54 (s, 3H), 1.68 (s, 9H) ppm.

^{13}C NMR (101 MHz, $CDCl_3$) δ 168.0, 150.2, 137.2, 135.5, 131.7, 125.2, 124.1, 117.6, 117.0, 115.3, 84.3, 52.8, 52.1, 51.4, 45.9, 28.4, 27.2 ppm.

Compound **16**

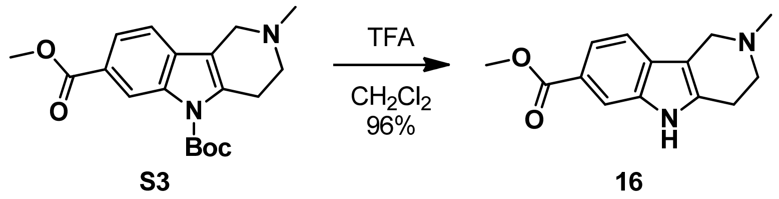

methyl 2-methyl-2,3,4,5-tetrahydro-1H-pyrido[4,3-b]indole-7-carboxylate (**16**): To a solution of **S3** (1.460 g, 4.24 mmol) in dichloromethane (8 mL) at 0 °C was added trifluoroacetic acid (10 mL, 131 mmol). The reaction mixture was allowed to warm up to room temperature and was stirred for 150 min at room temperature. The reaction was stopped with the removal of the excess of reagent and solvent *in vacuo*. The crude product was dissolved in dichloromethane (20 mL) and basified with an aqueous solution of sodium hydroxide (2 M, 20 mL). The aqueous and organic layers were separated and the aqueous layer was extracted with dichloromethane (6 x 8 mL). The combined organic layers were washed with brine, dried over magnesium sulfate, filtered and concentrated *in vacuo*. The desired indole **16** (990.0 mg, 4.05 mmol, 96%) was obtained as a yellow solid without further purification.

R_F 0.10 (CH_2Cl_2/MeOH 90/10).
^{1}H NMR (400 MHz, DMSO-d_6) δ 11.23 (s, 1H), 7.92 (d, J = 1.1 Hz, 1H), 7.58 (dd, J = 8.3, 1.5 Hz, 1H), 7.39 (d, J = 8.3 Hz, 1H), 3.83 (s, 3H), 3.53 (t, J = 1.5 Hz, 2H), 2.84 (t, J = 5.6 Hz, 2H), 2.72 (t, J = 5.5 Hz, 2H), 2.43 (s, 3H) ppm.
^{13}C NMR (101 MHz, DMSO-d_6) δ 167.3, 137.1, 135.1, 128.9, 121.0, 119.3, 116.7, 112.6, 108.1, 51.8, 51.7, 51.1, 45.5, 23.6 ppm.

Compound **S4**

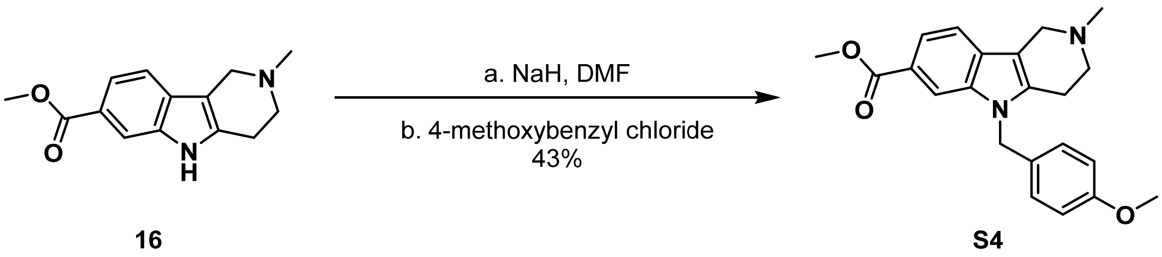

methyl 5-(4-methoxybenzyl)-2-methyl-2,3,4,5-tetrahydro-1H-pyrido[4,3-b]indole-7-carboxylate (**S4**): To a suspension of sodium hydride (60% in oil, 48.0 mg, 1.20 mmol) in dimethylformamide (11 mL) was added **16** (245.0 mg, 1.00 mmol). This suspension was stirred for 30 min, then 4-methoxybenzyl chloride (0.15 mL, 173.3 mg, 1.11 mmol) was added and the reaction mixture was stirred for 15 min at room temperature. The excess of base was consumed through the addition of a solution of saturated ammonium chloride (10 mL). The reaction mixture was extracted with dichloromethane (6 x 5 mL). The organic phase was washed with brine, dried over magnesium sulfate, filtered and concentrated *in vacuo*. Purification by flash chromatography (CH_2Cl_2/MeOH 95/5 to 90/10) afforded the desired product **S4** (156.0 mg, 0.43 mmol, 43%) as a white foam.

R$_F$ 0.76 (CH_2Cl_2/MeOH 90/10).

^{1}H NMR (400 MHz, $CDCl_3$) δ 8.02 (d, *J* = 1.1 Hz, 1H), 7.78 (dd, *J* = 8.3, 1.3 Hz, 1H), 7.44 (d, *J* = 8.3 Hz, 1H), 6.93 - 6.91 (m, 2H), 6.79 - 6.77 (m, 2H), 5.25 (s, 2H), 3.90 (s, 3H), 3.75 (s, 3H), 3.71 (br. s, 2H), 2.83 - 2.81 (m, 4H), 2.56 (s, 3H) ppm.

^{13}C NMR (101 MHz, $CDCl_3$) δ 168.5, 159.1, 137.5, 136.4, 129.5, 129.3, 127.5, 122.6, 120.6, 117.3, 114.4, 111.6, 109.2, 55.4, 52.4, 52.0, 51.8, 46.2, 45.9, 23.4 ppm.

LC-MS *m/z*: $[M+H]^+$ 365.

Compound **S5**

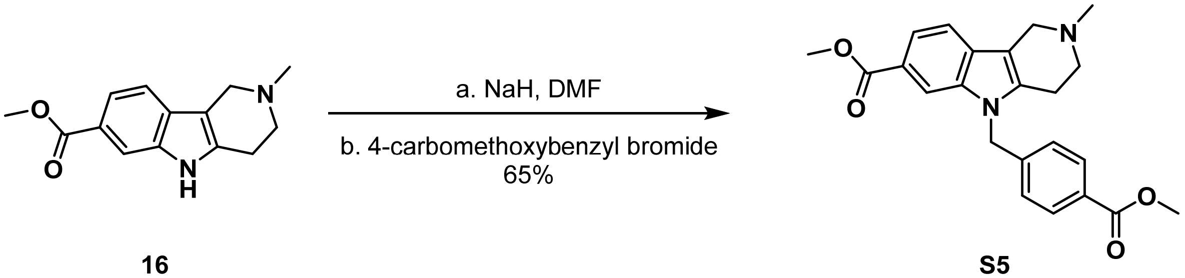

16 **S5**

Methyl 5-(4-(methoxycarbonyl)benzyl)-2-methyl-2,3,4,5-tetrahydro-1H-pyrido[4,3-b]indole-7-carboxylate (**S5**): To a suspension of sodium hydride (60% in oil, 49.0 mg, 1.22 mmol) in dimethylformamide (11 mL) was added **16** (247.0 mg, 1.01 mmol). This suspension was stirred for 30 min, then 4-carbomethoxybenzyl bromide (246.0 mg, 1.07 mmol) was added and the reaction mixture was stirred for 15 min at room temperature. The excess of base was consumed through the addition of a solution of saturated ammonium chloride (10 mL). The reaction mixture was extracted with ethyl acetate (3 x 5 mL). The organic phase was washed with brine, dried over magnesium sulfate, filtered and concentrated *in vacuo*. Purification by flash chromatography (CH_2Cl_2/MeOH 95/5 to 90/10) afforded the desired ester **S5** (311.2 mg, 0.79 mmol, 65%) as a yellow solid.

R$_F$ 0.71 (CH_2Cl_2/MeOH 90/10).

^{1}H NMR (400 MHz, $CDCl_3$) δ 7.96 (dd, *J* = 1.4, 0.7 Hz, 1H), 7.94 - 7.92 (m, 2H), 7.80 (dd, *J* = 8.3, 1.4 Hz, 1H), 7.46 (dd, *J* = 8.3, 0.6 Hz, 1H), 7.04 - 7.02 (m, 2H), 5.36 (s, 2H), 5.05 (t, *J* = 2.8 Hz, 1H), 3.89 - 3.88 (m, 6H), 3.73 (t, *J* = 1.5 Hz, 1H), 2.85 - 2.82 (m, 2H), 2.79 - 2.76 (m, 2H), 2.57 (s, 3H) ppm.

^{13}C NMR (101 MHz, $CDCl_3$) δ 168.3, 168.8, 142.6, 137.3, 136.3, 130.4, 129.7, 129.4, 126.2, 123.0, 120.9, 117.5, 111.4, 109.6, 52.3, 52.3, 52.0, 51.7, 46.5, 45.9, 23.3 ppm.

LC-MS *m/z:* $[M+H]^+$ 393.

Compound **2c**

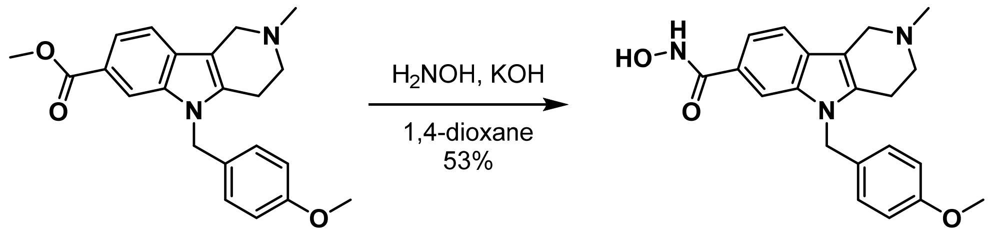

S4 2c

***N*-hydroxy-5-(4-methoxybenzyl)-2-methyl-2,3,4,5-tetrahydro-1H-pyrido[4,3-b]indole-7-carboxamide** (**2c**): To a solution of **S4** (150.0 mg, 411.6 µmol) in 1,4-dioxane (4 mL) were added an aqueous solution of hydroxylamine (50 wt%, 4 mL) and potassium hydroxide (93.0 mg, 1.657 mmol). The reaction mixture was stirred for 24 h at room temperature. The reaction was stopped with the addition of water (10 mL) and the mixture was extracted with ethyl acetate (5 x 5 mL). The organic phase was washed with brine, dried over magnesium sulfate, filtered and concentrated *in vacuo*. The crude solid was recrystallized in ethyl acetate:methanol and the obtained solid was washed with *n*-hexane to afford the desired product **2c** (80.0 mg, 0.22 mmol, 53%) as a white solid.

R$_F$ 0.21 (CH_2Cl_2/MeOH 90/10).
^{1}H NMR (600 MHz, DMSO-*d$_6$*) δ 11.05 (brs, 1H), 8.86 (brs, 1H), 7.88 (d, *J* = 0.6 Hz, 1H), 7.42 (dd, *J* = 8.3, 1.4 Hz, 1H), 7.39 (dd, *J* = 8.2, 0.7 Hz, 1H), 6.98 - 6.97 (m, 2H), 6.86-6.84 (m, 2H), 5.30 (s, 2H), 3.69 (s, 3H), 3.54 (brs, 2H), 2.77 (t, *J* = 5.3 Hz, 2H), 2.71 (d, *J* = 5.2 Hz, 2H), 2.41 (s, 3H) ppm.
HRMS-ESI *m/z*: $[M+H]^+$ calcd for $C_{21}H_{24}N_3O_3$: 366.1812; found: 366.1818.
mp = 183.2 °C (decomposition).

Compound **3c**

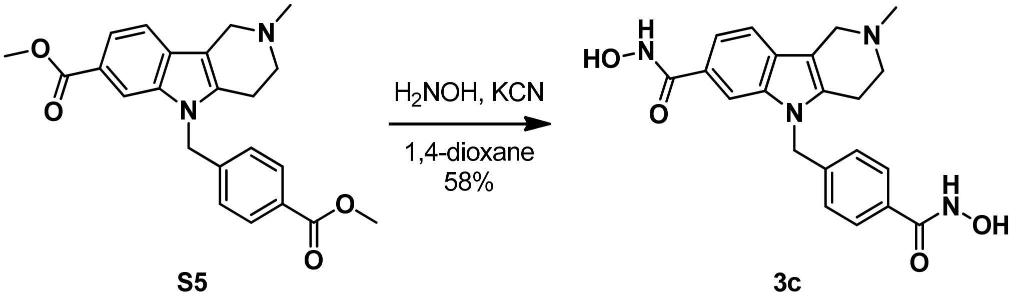

S5 3c

***N*-hydroxy-5-(4-(hydroxycarbamoyl)benzyl)-2-methyl-2,3,4,5-tetrahydro-1H-pyrido[4,3-b]indole-7-carboxamide** (**3c**): To a solution of **S5** (49.3 mg, 125.62 µmol) in 1,4-dioxane (1 mL) were added an aqueous solution of hydroxylamine (50 wt%, 2 mL) and potassium cyanide (35.2 mg, 540.1 µmol). The reaction mixture was stirred for 16 h at room temperature and then concentrated *in vacuo*. Purification of the crude material by HPLC afforded the TFA salt of the desired hydroxamic acid **3c** (29.0 mg, 0.057 mmol, 58%) as a white solid.

R$_F$ 0.21 (EtOAc/MeOH 50/50).
^{1}H NMR (400 MHz, DMSO-*d$_6$*) δ 11.17 (s, 1H), 11.12 (s, 1H), 10.10 (s, 1H), 8.98 (s, 1H), 7.95 (s, 1H), 7.69 - 7.67 (m, 2H), 7.56 - 7.50 (m, 2H), 7.13 - 7.11 (m, 2H), 5.59 - 5.45 (m, 2H), 4.71 - 4.67 (m, 1H), 4.37 - 4.31 (m, 1H), 3.78 - 3.77 (m, 2H), 3.18-3.05 (m, 2H), 3.00 (d, *J* = 3.6 Hz, 3H) ppm.

^{13}C NMR (101 MHz, DMSO-*d*$_6$) δ 164.8, 163.7, 140.7, 136.0, 133.9, 132.0, 127.3, 126.4, 126.3, 126.3, 118.5, 117.5, 115.1, 109.3, 102.7, 50.4, 45.0, 45.7, 41.9, 19.8 ppm.
HRMS-ESI *m/z*: $[M+Na]^+$ calcd for $C_{21}H_{22}N_4O_4Na$: 417.1533; found: 417.1541.
mp 98.1 °C.

Compound **S6**

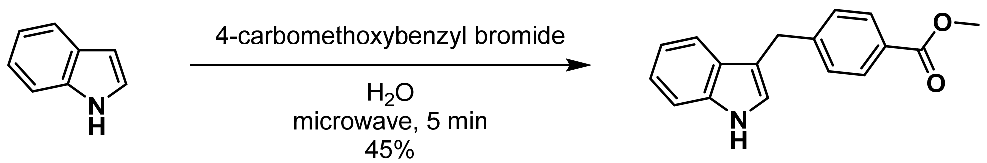

4 **S6**

methyl 4-((1H-indol-3-yl)methyl)benzoate (**S6**)[2, 3]: A mixture of 1H-indole **4** (1.02 g, 8.67 mmol) and 4-carbomethoxybenzyl bromide (1.9646 g, 8.58 mmol) in water (12 mL) was heated by microwave irradiation at 150 °C for 5 min. The reaction mixture was cooled to room temperature and a solution of saturated sodium hydrogen carbonate (20 mL) was added. The aqueous and organic layers were separated and the aqueous phase was extracted with dichloromethane (2 x 10 mL). The combined organic layers were washed with brine, dried over magnesium sulfate, filtered and concentrated *in vacuo*. Purification by flash chromatography (*n*-hexane:EtOAc 4:1) afforded the desired indole **S6** (1.024 mg, 3.86 mmol, 45%) as a light yellow oil.

R$_F$ 0.28 (*n*-hexane/EtOAc 80/20).
^{1}H NMR (400 MHz, DMSO-*d*$_6$) δ 10.90 (s, 1H), 7.87 - 7.84 (m, 2H), 7.43 - 7.33 (m, 4H), 7.20 (d, *J* = 2.4 Hz, 1H), 7.05 (ddd, *J* = 8.0, 7.0, 1.1 Hz, 1H), 6.92 (ddd, *J* = 8.0, 7.9, 1.0 Hz, 1H), 4.12 (s, 2H), 3.81 (s, 3H) ppm.
LC-MS *m/z:* $[M+H]^+$ 266.

Compound **1d**

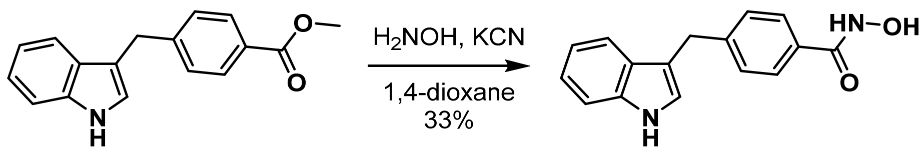

S6 **1d**

4-((1H-indol-3-yl)methyl)-*N*-hydroxybenzamide (**1d**): To a solution of **S6** (201.8 mg, 0.76 mmol) in 1,4-dioxane (4 mL) were added an aqueous solution of hydroxylamine (50 wt%, 4.0 mL) and potassium cyanide (53.2 mg, 0.82 mmol). The reaction mixture was stirred for 24 h at room temperature. The reaction was stopped with the removal of the excess of reagent and solvent *in vacuo*. Purification by HPLC afforded the desired hydroxamic acid **1d** (67.2 mg, 0.25 mmol, 33%) as a white solid.

R$_F$ 0.10 (*n*-hexane/EtOAc 50/50).
^{1}H NMR (400 MHz, DMSO-*d*$_6$) δ 11.10 (s, 1H), 10.87 (s, 1H), 8.84 (s, 1H), 7.65 - 7.62 (m, 2H), 7.41 - 7.39 (m, 1H), 7.35 - 7.32 (m, 3H), 7.19 (d, *J* = 2.3 Hz, 1H), 7.04 (ddd, *J* = 8.1, 7.0, 1.0 Hz, 1H), 6.92 (ddd, *J* = 7.9, 7.0, 1.0 Hz, 1H), 4.07 (s, 2H) ppm.

^{13}C NMR (101 MHz, DMSO-d_6) δ 164.3, 145.3, 136.4, 130.3, 128.3, 126.9, 126.9, 123.3, 121.0, 118.5, 118.3, 113.3, 111.4, 30.9 ppm.
HRMS-ESI *m/z*: $[M+Na]^+$ calcd for $C_{16}H_{14}N_2O_2Na$: 289.0947; found: 289.0950.
mp = 139.5 °C.

Compound **18**

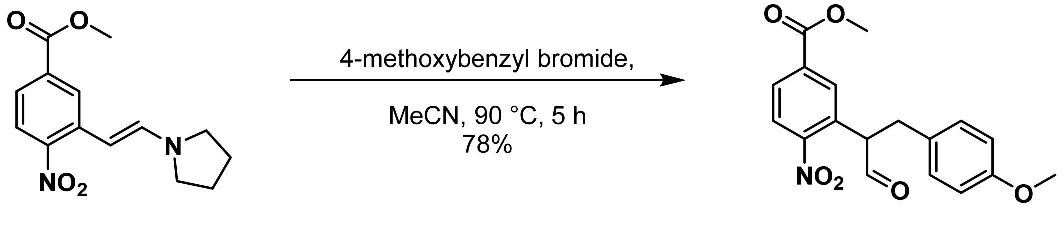

17 **18**

methyl 3-(1-(4-methoxyphenyl)-3-oxopropan-2-yl)-4-nitrobenzoate (**18**): The enamine **17**[4] (7.02 g, 25.41 mmol) and 4-methoxybenzyl bromide (5.11 g, 25.41 mmol) were dissolved in 15.0 mL of acetonitrile and heated to reflux for 5 h. After cooling to room temperature the mixture was diluted with water (5.0 mL) and concentrated on a rotary evaporator. The remaining black residue was purified by column chromatography (pure CH_2Cl_2) to afford the pure product **18** (6.8 g, 19.8 mmol, 78%).

R_F 0.5 (CH_2Cl_2).
^{1}H NMR (400 MHz, $CDCl_3$) δ 9.82 (s, 1H), 8.07 (dd, *J* = 8.4, 1.8 Hz, 1H), 7.96 (d, *J* = 1.8 Hz, 1H), 7.92 (dd, *J* = 8.5, 0.4 Hz, 1H), 7.02 – 6.97 (m, 2H), 6.78 – 6.73 (m, 2H), 4.52 (t, *J* = 7.5 Hz, 1H), 3.96 (s, 3H), 3.75 (s, 3H), 3.50 (dd, *J* = 14.1, 7.2 Hz, 1H), 3.05 (dd, *J* = 14.1, 7.8 Hz, 1H) ppm.

Compound **19**

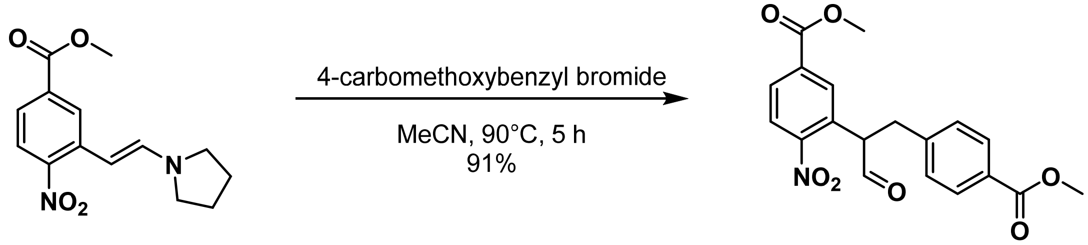

17 **19**

methyl 3-(1-(4-(methoxycarbonyl)phenyl)-3-oxopropan-2-yl)-4-nitrobenzoate (**19**): The enamine **17**[4] (11.94 g, 43.23 mmol) and 4-carbomethoxybenzyl bromide (9.93 g, 43.23 mmol) were dissolved in 30.0 mL of acetonitrile and heated to reflux for 5 h. After cooling to room temperature the mixture was diluted with water (10.0 mL) and concentrated on a rotary evaporator. The remaining black residue was purified by column chromatography (pure CH_2Cl_2) to afford the pure product **19** (14.55 g, 39.18 mmol, 91%).

R_F 0.20 - 0.30 (CH_2Cl_2).
^{1}H NMR (600 MHz, $CDCl_3$) δ 9.80 (s, 1H), 8.09 (dd, *J* = 8.4, 1.8 Hz, 1H), 7.94 (dd, *J* = 8.4, 0.4 Hz, 1H), 7.92 (d, *J* = 1.8 Hz, 1H), 7.90 – 7.88 (m, 2H), 7.17 – 7.14 (m, 2H), 4.59 – 4.53 (m, 1H), 3.96 (s, 3H), 3.88 (s, 3H), 3.62 (dd, *J* = 14.1, 7.0 Hz, 1H), 3.14 (dd, *J* = 14.1, 8.0 Hz, 1H) ppm.

Compound **20**

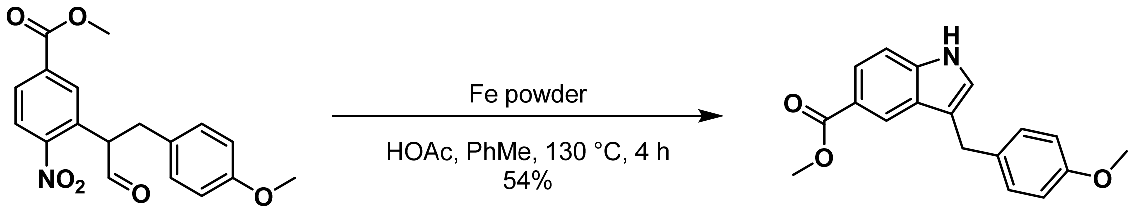

18 **20**

methyl 3-(4-methoxybenzyl)-1H-indole-5-carboxylate (**20**)[4]: The aldehyde **18** (6.8 g, 19.81 mmol) and iron powder (5.53 g, 99.05 mmol) were suspended in 70 mL of a mixture of glacial acetic acid / toluene (3:4) and heated to 130 °C under an atmosphere of nitrogen for 3.5 h. After cooling to room temperature the remaining solids (iron powder as well as iron acetate) were removed and the filtrate was concentrated. The mixture was diluted with water (100 mL) and extracted with toluene (3 x 100 mL). The combined organic phases were washed with saturated $NaHCO_3$ solution (150 mL) and brine (150 mL), dried over $MgSO_4$ and concentrated. The residue was purified by column chromatography (*n*-hexane/EtOAc 8:2 to 1:3) to afford a white solid as the pure product **20** (3.13 g, 10.61 mmol, 54%).

R$_F$ 0.6 (*n*-hexane/EtOAc 1:1)
^{1}H NMR (400 MHz, $CDCl_3$) δ 8.33 (dt, *J* = 1.6, 0.7 Hz, 1H), 8.16 (s, 1H), 7.90 (dd, *J* = 8.6, 1.6 Hz, 1H), 7.35 (dd, *J* = 8.6, 0.7 Hz, 1H), 7.22 – 7.17 (m, 2H), 6.93 (dt, *J* = 2.2, 1.0 Hz, 1H), 6.87 – 6.81 (m, 2H), 4.08 (d, *J* = 1.1 Hz, 2H), 3.92 (s, 3H), 3.78 (s, 3H) ppm.

Compound **21**

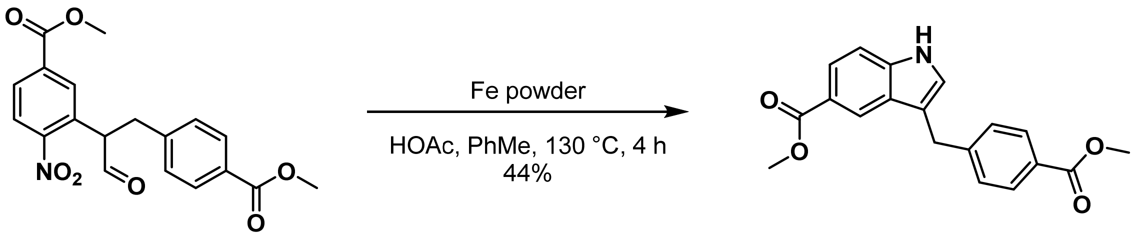

19 **21**

methyl 3-(4-(methoxycarbonyl)benzyl)-1H-indole-5-carboxylate (**21**)[4]: The aldehyde **19** (10.73 g, 28.89 mmol) and iron powder (8.07 g, 144.46 mmol) were suspended in 100 mL of a mixture of glacial acetic acid / toluene (2:3) and heated to 130 °C under an atmosphere of nitrogen for 3.5 h. After cooling to room temperature the remaining solids (iron powder as well as iron acetate) were removed and the filtrate was concentrated. The mixture was diluted with water (100 mL) and extracted with toluene (3 x 100 mL). The combined organic phases were washed with saturated $NaHCO_3$ solution (150 mL) and brine (150 mL), dried over $MgSO_4$ and left in the fridge for crystallization. The brown crystalline solid was filtered and washed with a small amount of dichloromethane and dried under high vacuum to afford the pure product **21** (4.10 g, 12.68 mmol, 44%).

^{1}H NMR (400 MHz, $CDCl_3$) δ 8.43 (br. s, 1H), 8.28 (s, 1H), 7.97 – 7.93 (m, 2H), 7.90 (dd, *J* = 8.6, 1.6 Hz, 1H), 7.36 (dd, *J* = 8.6, 0.7 Hz, 1H), 7.35 – 7.31 (m, 2H), 6.96 (dt, *J* = 2.2, 1.0 Hz, 1H), 4.18 (s, 2H), 3.91 (s, 3H), 3.89 (s, 3H) ppm.

Compound **2d**

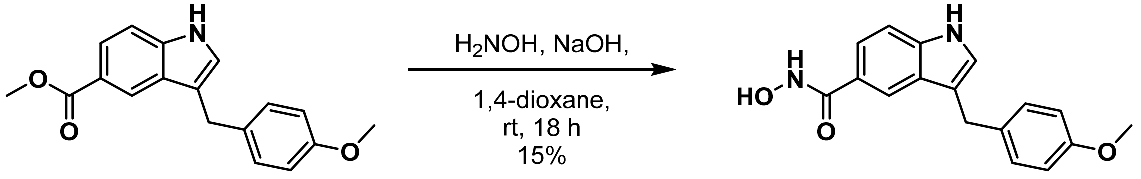

20 **2d**

***N*-hydroxy-3-(4-methoxybenzyl)-1H-indole-5-carboxamide** (**2d**): To a solution of **20** (205.0 mg, 0.69 mmol) in 2.0 mL of 1,4-dioxane was added a prepared mixture of sodium hydroxide (277.6 mg, 6.90 mmol) in an aqueous solution of hydroxylamine (50 wt%, 2.0 mL). The reaction mixture was stirred at room temperature for 18 h and then concentrated on a rotary evaporator. The crude material was subjected to column chromatography (CH_2Cl_2/MeOH 90/10) to afford the pure product **2d** as a pale-red solid (30.2 mg, 0.10 mmol, 15%).

R$_F$ 0.10 (CH_2Cl_2/MeOH 90/10).
^{1}**H NMR** (400 MHz, DMSO-*d*$_6$) δ 11.07 (s, 1H), 11.01 (s, 1H), 8.79 (d, *J* = 1.9 Hz, 1H), 7.98 – 7.93 (m, 1H), 7.50 (dd, *J* = 8.5, 1.7 Hz, 1H), 7.34 (dd, *J* = 8.6, 0.7 Hz, 1H), 7.22 – 7.17 (m, 3H), 6.85 – 6.80 (m, 2H), 3.98 (s, 2H), 3.69 (s, 3H) ppm.
^{13}**C NMR** (100 MHz, DMSO-*d*$_6$) δ 165.7, 157.3, 137.9, 133.4, 129.3, 126.4, 124.3, 123.0, 120.0, 118.2, 115.4, 113.6, 111.0, 55.0, 29.9 ppm.
HRMS-ESI *m/z*: $[M + Na]^+$ calcd for $C_{17}H_{16}N_2NaO_3$: 319.1054; found: 319.1055.

Compound **3d**

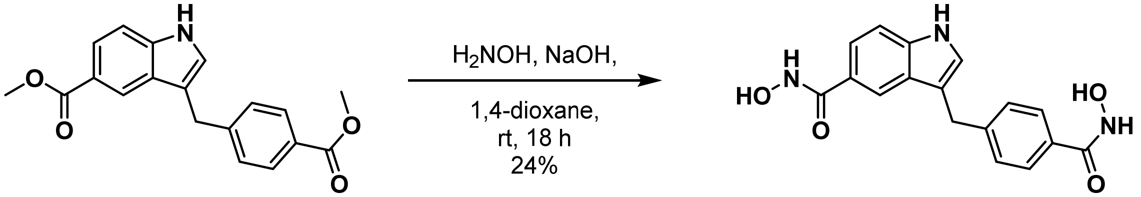

21 **3d**

***N*-hydroxy-3-(4-(hydroxycarbamoyl)benzyl)-1H-indole-5-carboxamide** (**3d**): To a solution of **21** (135.0 mg, 0.42 mmol) in 2.0 mL of 1,4-dioxane was added a prepared mixture of sodium hydroxide (252.0 mg, 6.3 mmol) in an aqueous solution of hydroxylamine (50 wt%, 2.0 mL). The reaction mixture was stirred at room temperature for 18 h and then concentrated on a rotary evaporator. The crude material was subjected to column chromatography (CH_2Cl_2/MeOH 90/10) to afford the pure product **3d** as a pale-red solid (31.5 mg, 0.10 mmol, 24%).

R$_F$ 0.08 (CH_2Cl_2/MeOH 90/10).
^{1}**H NMR** (400 MHz, DMSO-*d*$_6$) δ 11.14 (d, *J* = 2.3 Hz, 1H), 11.11 (s, 1H), 11.02 (s, 1H), 7.96 – 7.94 (m, 1H), 7.67 – 7.63 (m, 2H), 7.51 (dd, *J* = 8.5, 1.6 Hz, 1H), 7.38 – 7.33 (m, 3H), 7.27 (d, *J* = 2.3 Hz, 1H), 4.10 (s, 2H) ppm.
^{13}**C NMR** (100 MHz, DMSO-*d*$_6$) δ 165.7, 164.3, 145.0, 137.9, 130.4, 128.3, 127.0, 126.4, 124.7, 123.1, 120.1, 118.1, 114.4, 111.1, 30.6 ppm.
HRMS-ESI *m/z*: $[2M + Na]^+$ calcd for $C_{34}H_{30}N_6NaO_8$: 673.2018; found: 673.2021.

Compound **22**

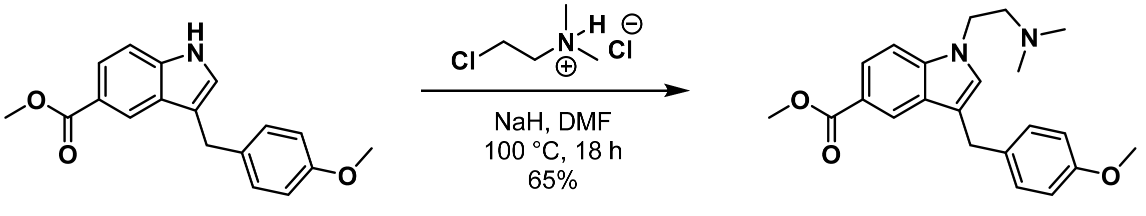

20 **22**

methyl 1-(2-(dimethylamino)ethyl)-3-(4-methoxybenzyl)-1H-indole-5-carboxylate (**22**): The indole **20** (303.7 mg, 1.03 mmol) was dissolved in 5.0 mL of dry DMF at room temperature. Then sodium hydride (60 wt% in mineral oil, 90.4 mg, 2.26 mmol) was added and the resulting mixture was stirred for 1.5 h, whereupon 2-chloroethyl-*N*,*N*-dimethylamine hydrochloride (163.2 mg, 1.13 mmol) was added. The mixture was heated to 100 °C for 18 h. After cooling to room temperature the reaction mixture was diluted with 20.0 mL of saturated $NaHCO_3$ solution and extracted with ethyl acetate (3 x 100 mL). The combined organic phases were washed with a saturated solution of $NaHCO_3$ (1 x 100 mL), water (1 x 100 mL) and brine (2 x 100 mL), dried over $MgSO_4$, filtered and concentrated. The crude material was purified by column chromatography (*n*-hexane/EtOAc 1/1) to afford an off-white solid as the pure product **22** (245.8 mg, 0.67 mmol, 65%).

^{1}H NMR (400 MHz, $CDCl_3$) δ 8.30 (s, 1H), 7.90 (dd, *J* = 8.7, 1.6 Hz, 1H), 7.30 (dd, *J* = 8.7, 0.7 Hz, 1H), 7.21 – 7.15 (m, 2H), 6.87 – 6.80 (m, 3H), 4.16 (t, *J* = 7.2 Hz, 2H), 4.05 (s, 2H), 3.91 (s, 3H), 3.78 (s, 3H), 2.70 – 2.63 (m, 2H), 2.27 (s, 6H) ppm.

Compound **2e**

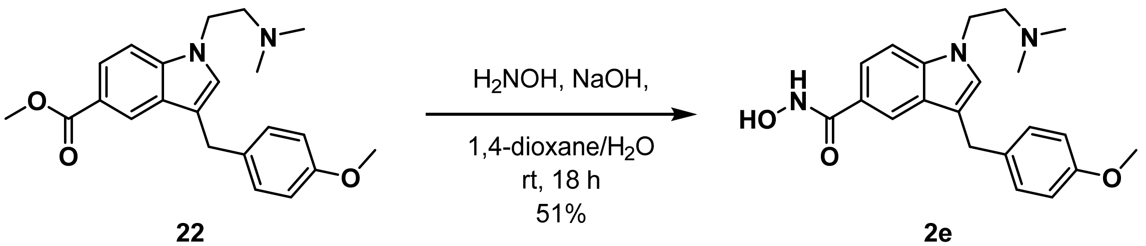

22 **2e**

1-(2-(dimethylamino)ethyl)-*N*-hydroxy-3-(4-methoxybenzyl)-1H-indole-5-carboxamide (**2e**): To a solution of **22** (245.8 mg, 0.67 mmol) in 2.0 ml of 1,4-dioxane was added a prepared mixture of NaOH (268.0 mg, 6.7 mmol) in an aqueous solution of NH_2OH (50 wt%, 1.5 mL). The reaction mixture was stirred at room temperature for 18 h and then concentrated in vacuo. The crude material was purified by column chromatography (CH_2Cl_2/MeOH 90/10) affording **2e** (123.5 mg, 0.34 mmol, 51%) as on off-white solid.

R_F 0.15 (CH_2Cl_2/MeOH 90/10).
^{1}H NMR (400 MHz, DMSO-d_6) δ 11.06 (s, 1H), 8.83 (s, 1H), 7.96 (t, *J* = 1.1 Hz, 1H), 7.55 (dd, *J* = 8.6, 1.7 Hz, 1H), 7.46 (dd, *J* = 8.7, 0.6 Hz, 1H), 7.24 (s, 1H), 7.21 – 7.16 (m, 2H), 6.88 – 6.78 (m, 2H), 4.24 (t, *J* = 6.6 Hz, 2H), 3.97 (s, 2H), 3.69 (s, 3H), 2.68 – 2.58 (m, 2H), 2.20 (s, 6H) ppm.
^{13}C NMR (101 MHz, DMSO-d_6) δ 165.5, 157.4, 137.6, 133.2, 129.3, 127.9, 126.8, 123.1, 112.0, 118.4, 115.0, 113.7, 109.3, 58.4, 55.0, 45.1, 43.4, 29.8 ppm.

HRMS-ESI *m/z*: $[M + Na]^+$ calcd for $C_{21}H_{25}N_3NaO_3$: 390.1789; found: 390.1798.

Compound **23**

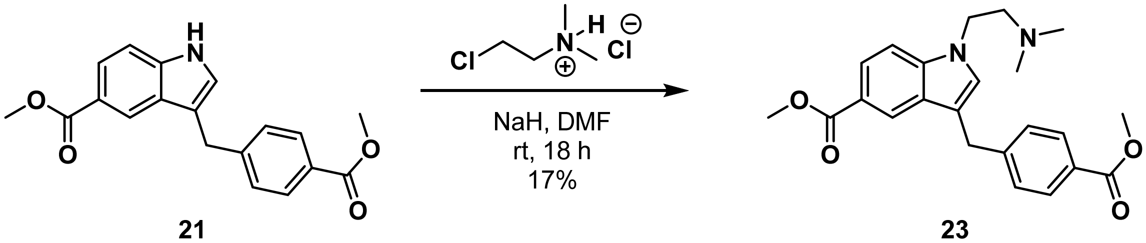

21 23

methyl 1-(2-(dimethylamino)ethyl)-3-(4-(methoxycarbonyl)benzyl)-1H-indole-5-carboxylate (23): The indole **21** (503.6 mg, 1.56 mmol) dissolved in 5.0 mL of dry DMF at room temperature. Then sodium hydride (60 wt% in mineral oil, 137.3 mg, 3.43 mmol) was added and the resulting mixture was stirred for 30 min, whereupon 2-chloroethyl-*N*,*N*-dimethylamine hydrochloride (269.4 mg, 1.87 mmol) was added. The reaction mixture was stirred at room temperature for 18 h. The reaction mixture was diluted with 20.0 mL of saturated $NaHCO_3$ solution and extracted with ethyl acetate (3 x 100 mL). The combined organic phases were washed with a saturated solution of $NaHCO_3$ (1 x 100 mL), water (1 x 100 mL) and brine (2 x 100 mL), dried over $MgSO_4$, filtered and concentrated. The crude material was purified by column chromatography (CH_2Cl_2/MeOH 95/5) to afford an off-white solid as the pure product **23** (106.7 mg, 0.27 mmol, 17%).

R$_F$ 0.47 (CH_2Cl_2/MeOH 95/5).
^{1}H NMR (400 MHz, $CDCl_3$) δ 8.25 (dd, *J* = 1.7, 0.7 Hz, 1H), 7.98 – 7.93 (m, 2H), 7.91 (dd, *J* = 8.7, 1.6 Hz, 1H), 7.36 – 7.30 (m, 3H), 6.91 – 6.88 (m, 1H), 4.21 – 4.15 (m, 4H), 3.90 (s, 3H), 3.89 (s, 3H), 2.67 (t, *J* = 7.1 Hz, 2H), 2.28 (s, 6H) ppm.

Compound **3e**

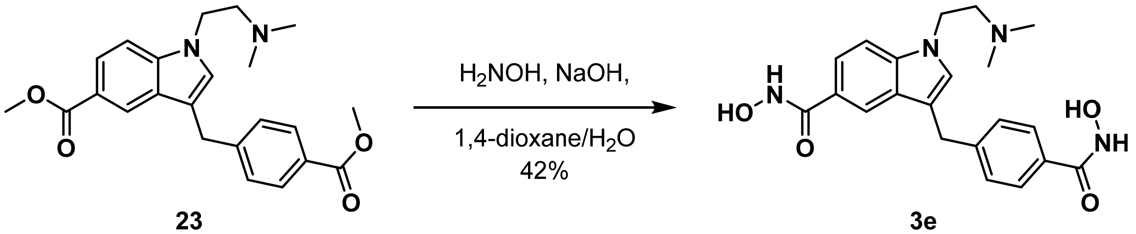

23 3e

1-(2-(dimethylamino)ethyl)-*N*-hydroxy-3-(4-(hydroxycarbamoyl)benzyl)-1H-indole-5-carboxamide (**3e**): To a solution of **23** (106.7 mg, 0.27 mmol) in 1.0 mL of 1,4-dioxane was added a prepared mixture of sodium hydroxide (108.0 mg, 2.7 mmol) in an aqueous solution of hydroxylamine (50 wt%, 1.5 mL). The reaction mixture was stirred at room temperature for 18 h and then concentrated on a rotary evaporator. The crude material was purified by HPLC affording an off-white solid as the pure product **3e** as its trifluoroacetate salt (58.4 mg, 0.11 mmol, 42%). The compound is extremely hygroscopic.

^{1}H NMR (400 MHz, DMSO-d_6) δ 11.13 (s, 1H), 11.11 (s, 1H), 10.04 (s, 1H), 8.96 (br. s, 1H), 8.88 (br. s, 1H), 8.01 (dd, *J* = 1.7, 0.7 Hz, 1H), 7.69 – 7.65 (m, 2H), 7.63 (dd, *J* = 8.7, 1.6 Hz, 1H), 7.58 (dd,

J = 8.7, 0.7 Hz, 1H), 7.38 – 7.34 (m, 2H), 7.32 (s, 1H), 4.58 – 4.50 (m, 2H), 4.10 (s, 2H), 3.48 (t, J = 7.2 Hz, 2H), 2.82 (s, 6H) ppm.
^{13}C NMR (101 MHz, DMSO-d_6) δ 165.2, 164.1, 158.1 (q, J = 31.0 Hz), 144.4, 137.5, 130.5, 128.4, 127.8, 127.0, 127.0, 123.9, 120.6, 118.5, 117.2 (q, J = 300.0 Hz), 115.2, 109.6, 55.1, 42.7, 40.6, 30.4 ppm.
HRMS-ESI *m/z*: [2M + H]$^+$ calcd for $C_{42}H_{49}N_8O_8$: 793.3668; found: 793.3677.

Compound **27**

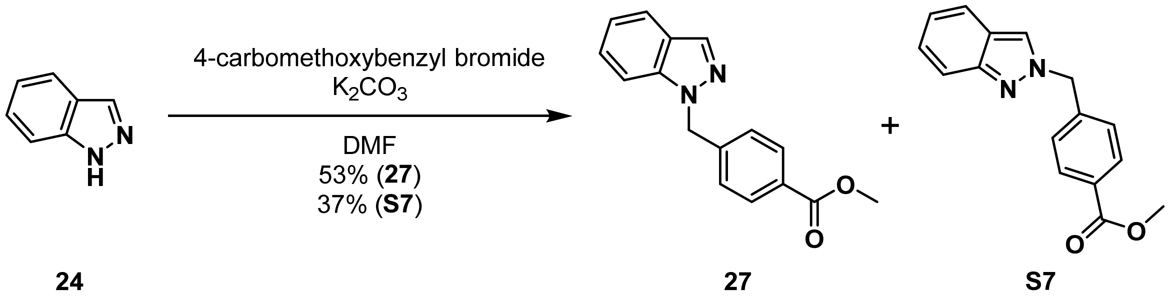

methyl 4-((1H-indazol-1-yl)methyl)benzoate (**27**): To a solution of 1H-indazole **24** (1.1921 g, 10.09 mmol) and 4-carbomethoxybenzyl bromide (2.7715 g, 12.10 mmol) in dimethylformamide (10 mL) was added potassium carbonate (2.0298 g, 14.69 mmol). The suspension was stirred for 16 h at room temperature. The mixture was diluted with water (50 mL) and afterwards extracted with ethyl acetate (2 x 10 mL). The organic phase was washed with brine, dried over magnesium sulfate, filtered and concentrated *in vacuo*. Purification by flash chromatography (*n*-hexane/EtOAc 75/25 to 66/33) afforded the desired esters **27** (1.422 g, 5.34 mmol, 53%) and **S7** (999.3 mg, 3.75 mmol, 37%) both as a white solids.

27:
R$_F$ 0.72 (*n*-hexane/EtOAc 50/50).
^{1}H NMR (400 MHz, DMSO-d_6) δ 8.16 (s, 1H), 7.90 - 7.88 (m, 2H), 7.78 (d, J = 8.0 Hz, 1H), 7.67 (d, J = 8.6 Hz, 1H), 7.38 - 7.34 (m, 1H), 7.32 - 7.30 (m, 2H), 7.13 (t, J = 7.4 Hz, 1H), 5.76 (s, 2H), 3.79 (s, 3H) ppm.
^{13}C NMR (101 MHz, DMSO-d_6) δ 165.9, 142.9, 139.4, 133.5, 129.4, 128.8, 127.4, 126.3, 123.8, 121.0, 120.6, 109.6, 52.0, 51.4 ppm.
LC-MS *m/z*: [M + H]$^+$ 267.

S7:
R$_F$ 0.58 (*n*-hexane/EtOAc 50/50).
^{1}H NMR (400 MHz, DMSO-d_6) δ 8.52 (s, 1H), 7.96 - 7.93 (m, 2H), 7.72 (dt, J = 8.4, 1.1 Hz, 1H), 7.63 (dq, J = 8.8, 1.0 Hz, 1H), 7.42 - 7.39 (m, 2H), 7.24 (ddd, J = 8.6, 6.6, 1.1 Hz, 1H), 7.04 (ddd, J = 8.4, 6.7, 0.8 Hz, 1H), 5.76 (s, 2H), 3.82 (s, 3H) ppm.
^{13}C NMR (101 MHz, DMSO-d_6) δ 165.9, 148.5, 142.3, 129.5, 129.1, 127.9, 125.6, 124.4, 121.6, 121.2, 120.7, 117.1, 55.8, 52.1 ppm.
LC-MS *m/z:* [M + H]$^+$ 267.

Compound **1f**

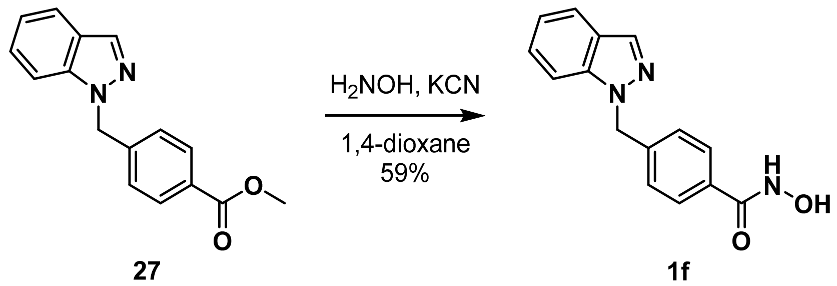

4-((1H-indazol-1-yl)methyl)-*N*-hydroxybenzamide (**1f**): To a solution of **27** (665.7 mg, 2.50 mmol) in 1,4-dioxane (12 mL) were added an aqueous solution of hydroxylamine (50 wt%, 10.0 mL) and potassium cyanide (87.8 mg, 1.35 mmol). The reaction mixture was stirred 24 h at room temperature. The reaction was stopped with the addition of a solution of saturated sodium hydrogen carbonate (50 mL) and ethyl acetate (25 mL). A solid precipitated which was filtered, washed with ethyl acetate as well as water and dried to afford the desired hydroxamic acid **1f** (396.3 mg, 1.48 mmol, 59%) as a white solid which was used without further purification.

$\mathbf{R_F}$ 0.48 (CH_2Cl_2/MeOH 90/10).
^{1}H NMR (400 MHz, DMSO-d_6) δ 11.14 (s, 1H), 9.01 (s, 1H), 8.13 (d, *J* = 1.0 Hz, 1H), 7.78 (dt, *J* = 8.1, 1.0 Hz, 1H), 7.72 – 7.64 (m, 3H), 7.38 (ddd, *J* = 8.3, 6.9, 1.1 Hz, 1H), 7.30 – 7.21 (m, 2H), 7.14 (ddd, *J* = 8.0, 6.9, 0.9 Hz, 1H), 5.71 (s, 2H) ppm.
HRMS-ESI *m/z*: $[M + Na]^+$ calcd for $C_{15}H_{13}N_3O_2Na$: 290.0900; found: 290.0903.
mp 170.2 °C.

Compound **28**

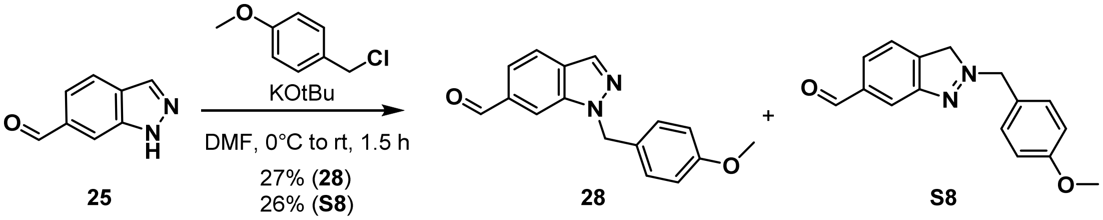

1-(4-methoxybenzyl)-1H-indazole-6-carbaldehyde (**28**): **28** was synthesized according to a published procedure.[5] Therefore, 1*H*-indazole-6-carbaldehyde **25** (0.958 g, 6.55 mmol, 1.0 eq.) and KO^{t}Bu (0.883 g, 7.867 mmol, 1.2 eq.) were dissolved in DMF (10 mL) and stirred at 0 °C for 10 min. 4-methoxybenzyl chloride (0.958 g, 6.55 mmol, 1.0 eq.) was added to the solution and stirring was continued at room temperature for 1.5 h. The reaction mixture was quenched with NH_4Cl (40 mL). The product was extracted with diethylether (3×50 mL). The combined organic layers were washed with brine (30 mL), dried with $MgSO_4$ and the solvent removed under reduced pressure. The residue was pre-loaded on coarse silica gel. Purification by column chromatography using hexane/ethyl acetate (8/2, R_F = 0.29) as eluent afforded a brown oil as the product **28** (0.48 g, 1.78 mmol, 27%) and **S8** (0.46 g, 1.73 mmol, 26%).

28:
$\mathbf{R_F}$ 0.29 (*n*-hexane/EtOAc 8/2).

^{1}H NMR (400 MHz, $CDCl_3$) δ 10.08 (s, 1H), 8.11 (d, *J* = 1.0 Hz, 1H), 7.90 (dd, *J* = 1.0 Hz, 1H), 7.88 – 7.82 (m, 1H), 7.67 (dd, *J* = 8.3, 1.2 Hz, 1H), 7.22 – 7.17 (m, 2H), 6.87 – 6.81 (m, 2H), 5.61 (s, 2H), 3.77 (s, 3H) ppm.
LC-MS *m*/*z*: $[M + H]^+$ 267.

S8:
R_F 0.12 (*n*-hexane/EtOAc 8/2).
^{1}H NMR (400 MHz, $CDCl_3$) δ 10.07 (d, *J* = 0.7 Hz, 1H), 8.24 (q, *J* = 1.0 Hz, 1H), 7.91 (d, *J* = 0.9 Hz, 1H), 7.69 (dt, *J* = 8.7, 0.8 Hz, 1H), 7.60 (dd, *J* = 8.7, 1.3 Hz, 1H), 7.31 – 7.27 (m, 2H), 6.93 – 6.89 (m, 2H), 5.58 (s, 2H), 3.81 (s, 3H) ppm.

Compound **29**

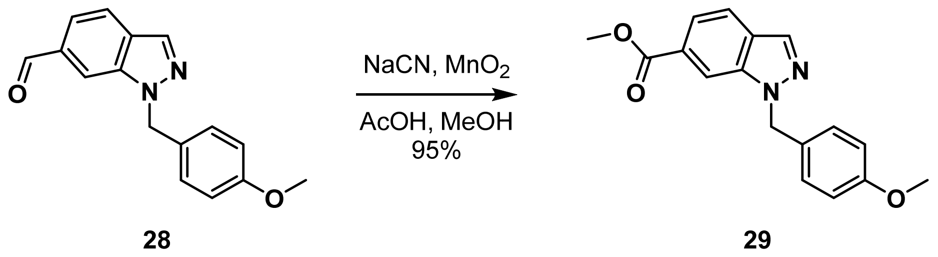

methyl 1-(4-methoxybenzyl)-1H-indazole-6-carboxylate (**29**)[5]: To a solution of aldehyde **28** (206.1 mg, 0.77 mmol) in 8.0 mL of methanol (with a few drops of CH_2Cl_2) were added sodium cyanide (113.8 mg, 2.32 mmol), acetic acid (0.13 mL, 2.32 mmol) and manganese dioxide (2.01 g, 23.22 mmol). The reaction mixture was stirred at room temperature for 2 h and afterwards filtered through a pad of celite which was thoroughly washed with CH_2Cl_2 (100 mL). The organic phase was washed with a saturated solution of $NaHCO_3$ (3 x 100 mL) and brine (1 x 150 mL), dried over $MgSO_4$ and concentrated to afford the product **29** (216.2 mg, 0.73 mmol, 95%) which was used in the next step without further purification.

^{1}H NMR (400 MHz, $CDCl_3$) δ 8.17 (q, *J* = 1.1 Hz, 1H), 8.07 (d, *J* = 1.0 Hz, 1H), 7.80 (dd, *J* = 8.5, 1.3 Hz, 1H), 7.76 (dd, *J* = 8.5, 0.9 Hz, 1H), 7.23 – 7.15 (m, 2H), 6.88 – 6.79 (m, 2H), 5.58 (s, 2H), 3.95 (s, 3H), 3.76 (s, 3H) ppm.

Compound **2f**

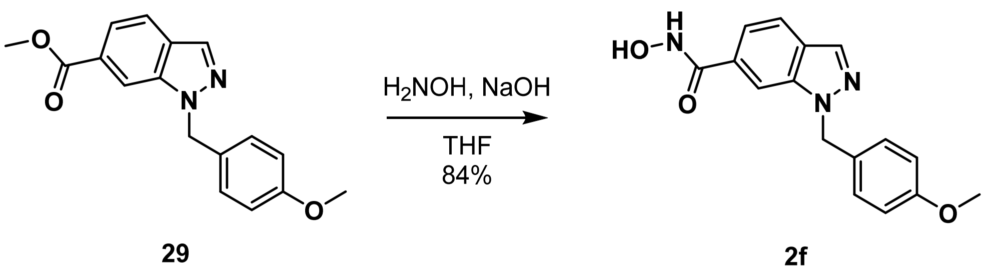

***N*-hydroxy-1-(4-methoxybenzyl)-1H-indazole-6-carboxamide** (**2f**): The ester **29** (106.0 mg, 0.36 mmol) was dissolved in 1.5 mL THF and a solution of sodium hydroxide (67.0 mg, 1.78 mmol) dissolved in 1.0 mL of an aqueous solution of hydroxylamine (50 wt%) was added. The mixture was stirred at room temperature for 1 h, diluted with water (5.0 mL) and THF was removed on a rotary evaporator. The remaining aqueous solution was adjusted to pH 7-8 with 1N HCl and afterwards

extracted with ethyl acetate (3 x 15 mL). The combined organic phases were washed with brine (30.0 mL), dried over $MgSO_4$, filtered and concentrated under high vacuum to afford a white powder as the pure product **2f** (89.0 mg, 0.30 mmol, 84%).

^{1}H NMR (400 MHz, DMSO-*d$_6$*) δ 11.28 (br. s, 1H), 9.09 (br. s, 1H), 8.15 (d, *J* = 0.9 Hz, 1H), 8.12 (d, *J* = 1.3 Hz, 1H), 7.81 (dd, *J* = 8.4, 0.8 Hz, 1H), 7.49 (dd, *J* = 8.5, 1.3 Hz, 1H), 7.25 – 7.19 (m, 2H), 6.90 – 6.83 (m, 2H), 5.61 (s, 2H), 3.69 (s, 3H) ppm.
^{13}C NMR (100 MHz, DMSO-*d$_6$*) δ 164.4, 158.7, 138.6, 133.0, 130.7, 129.3, 128.8, 125.1, 120.9, 119.00, 113.9, 109.0, 55.1, 51.4 ppm.
HRMS-ESI *m/z*: [M – H]$^-$ calcd for $C_{16}H_{14}N_3O_3$: 296.1040; found: 296.1039.

Compound **30**

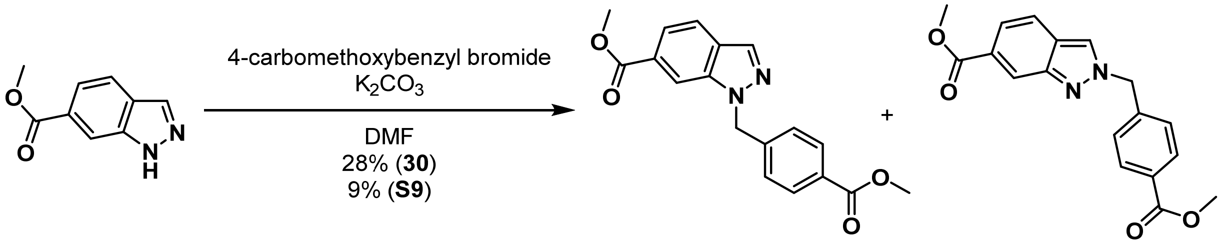

26 **30** **S9**

methyl 1-(4-(methoxycarbonyl)benzyl)-1H-indazole-6-carboxylate (**30**)[6]: To a solution of methyl 1H-indazole-6-carboxylate **26** (1.76 g, 10.06 mmol) and 4-carbomethoxybenzyl bromide (2.77 g, 12.07 mmol) in dimethylformamide (5 mL) was added potassium carbonate (2.0189 g, 14.61 mmol). The suspension was stirred for 16 h at room temperature. The mixture was diluted with water (50 mL) and afterwards extracted with ethyl acetate (2 x 10 mL). The organic phase was washed with brine, dried over magnesium sulfate, filtered and concentrated *in vacuo*. Purification by flash chromatography (*n*-hexane:EtOAc - 75:25 to 66:33) afforded the desired esters **30** (1.098 g, 3.38 mmol, 28%) and **S9** (337.8 mg, 1.04 mmol, 9%) both as a light yellow solids.

30:
R_F 0.51 (*n*-hexane/EtOAc 50/50).
^{1}H NMR (400 MHz, DMSO-*d$_6$*) δ 8.36 (q, *J* = 1.1 Hz, 1H), 8.28 (d, *J* = 1.0 Hz, 1H), 7.92 – 7.87 (m, 3H), 7.71 (dd, *J* = 8.5, 1.3 Hz, 1H), 7.31 – 7.26 (m, 2H) ppm.
^{13}C NMR (101 MHz, DMSO *d$_6$*) δ 166.5, 165.9, 142.8, 138.9, 133.8, 129.5, 128.8, 127.6, 127.3, 126.3, 121.4, 120.7, 111.7, 52.3, 52.1, 51.5 ppm.
LC-MS *m/z*: [M + H]$^+$ 325.

S9:
R_F 0.41 (*n*-hexane/EtOAc 50/50).
^{1}H NMR (400 MHz, DMSO-*d$_6$*) δ 8.66 (s, 1H), 8.34 – 8.26 (m, 1H), 8.00 – 7.89 (m, 2H), 7.88 – 7.79 (m, 1H), 7.57 (dd, *J* = 8.7, 1.3 Hz, 1H), 7.47 – 7.38 (m, 2H), 5.82 (s, 2H), 3.87 (s, 3H), 3.85 (s, 3H) ppm.
LC-MS *m/z*: [M + H]$^+$ 325.

Compound **3f**

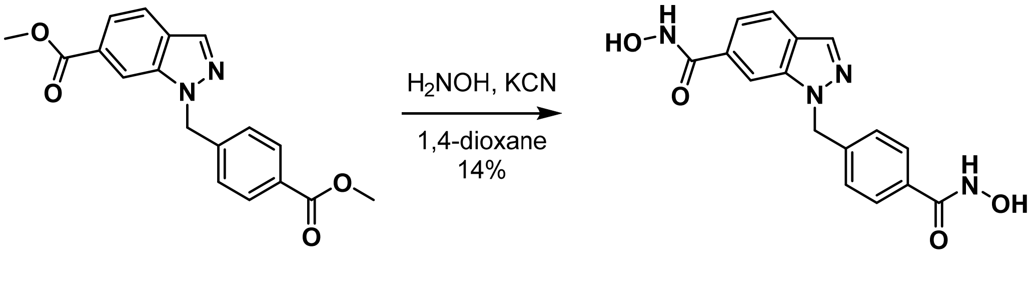

30 **3f**

***N*-hydroxy-1-(4-(hydroxycarbamoyl)benzyl)-1H-indazole-6-carboxamide** (**3f**): To a solution of **30** (387.7 g, 1.20 mmol) in 1,4-dioxane (12 mL) were added an aqueous solution of hydroxylamine (50 wt%, 12.0 mL) and potassium cyanide (52.3 mg, 803.2 µmol). The reaction mixture was stirred for 48 h at room temperature. The reaction was stopped with the removal of the excess of reagent and solvent *in vacuo*. Purification by HPLC afforded the desired hydroxamic acid **3f** (56.3 mg, 0.17 mmol, 14%) as a white solid.

R$_F$ 0.75 (CH_2Cl_2/MeOH 50/50).
^{1}H NMR (600 MHz, DMSO-d_6) δ 11.27 (s, 1H), 11.13 (s, 1H), 9.04 (br.s, 2H), 8.20 (d, J = 1.0 Hz, 1H), 8.14 – 8.10 (m, 1H), 7.84 (dd, J = 8.4, 0.8 Hz, 1H), 7.70 – 7.66 (m, 2H), 7.51 (dd, J = 8.4, 1.3 Hz, 1H), 7.29 – 7.25 (m, 2H), 5.75 (s, 2H) ppm.
^{13}C NMR (151 MHz, DMSO-d_6) δ 164.4, 163.9, 140.4, 138.9, 133.5, 132.1, 130.9, 127.2, 127.2, 125.1, 121.0, 119.1, 108.9, 51.5 ppm.
HRMS-ESI *m/z*: [M + Na]$^+$ calcd for $C_{16}H_{14}N_4O_4Na$: 349.0907; found: 349.0911.
mp 72.5 °C.

Compound **S10**

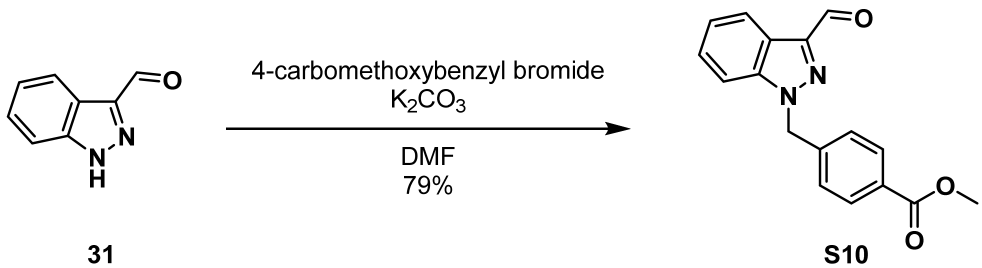

31 **S10**

methyl 4-((3-formyl-1H-indazol-1-yl)methyl)benzoate (**S10**): To a solution of 1H-indazole-3-carbaldehyde **31** (522.7 mg, 3.58 mmol) and 4-carbomethoxybenzyl bromide (1.02 g, 4.46 mmol) in dimethylformamide (5 mL) was added potassium carbonate (742.5 mg, 5.37 mmol). The suspension was stirred for 16 h at room temperature. The mixture was diluted with water (50 mL) and afterwards extracted with ethyl acetate (2 x 10 mL). The organic phase was washed with brine, dried over magnesium sulfate, filtered and concentrated *in vacuo*. Purification by flash chromatography (*n*-hexane/EtOAc 80/20) afforded the desired product **S10** (827.7 mg, 2.81 mmol, 79%) as a white solid.

R$_F$ 0.51 (*n*-hexane/EtOAc 2/1).
^{1}H NMR (400 MHz, DMSO-d_6) δ 10.15 (s, 1H), 8.13 (d, J = 8.1 Hz, 1H), 7.89 - 7.84 (m, 3H), 7.50 (t, J = 7.2 Hz, 1H), 7.38 - 7.33 (m, 3H), 5.91 (s, 2H), 3.77 (s, 3H) ppm.

^{13}C NMR (100 MHz, DMSO-d_6) δ 186.9, 165.8, 142.7, 141.5, 140.9, 129.6, 129.2, 127.8, 127.7, 124.4, 121.3, 121.1, 110.9, 52.5, 52.1 ppm.
LC-MS *m/z*: $[M + H]^+$ 295.

Compound **S11**

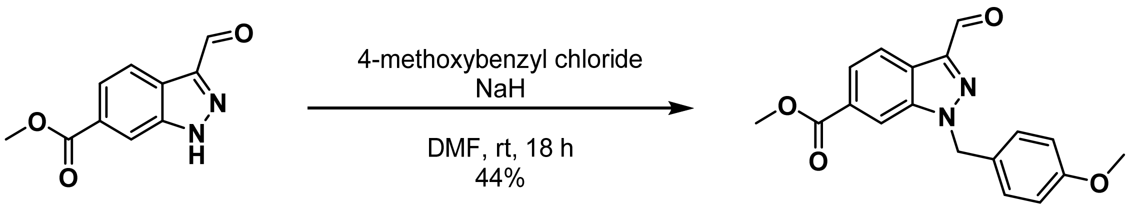

32 **S11**

methyl 3-formyl-1-(4-methoxybenzyl)-1H-indazole-6-carboxylate (**S11**): The aldehyde **32** (1.0 g, 4.90 mmol) was dissolved in 10.0 mL DMF at 0 °C whereupon sodium hydride (0.24 g, 5.88 mmol) was added. The suspension was stirred for 5 minutes at 0 °C and 30 min at room temperature. Then 4-methoxybenzyl chloride (0.92 g, 0.80 mL, 5.88 mmol) was added and the resulting mixture was stirred at room temperature for 18 h. The reaction was quenched by the addition of a saturated $NaHCO_3$ solution (100.0 mL). The mixture was extracte with ethyl acetate (3 x 100 mL) and the combine organic phases were washed with saturated $NaHCO_3$ solution (150 mL) and brine (150 mL), dried over $MgSO_4$, filtered and concentrated. The crude material was purified by column chromatography (petrol ether/EtOAc 2:1). The resulting product was contaminated with the *N2*-substituted regioisomer, but could be purified by recrystallized from toluene to obtain **S11** (0.71 g, 2.19 mmol, 44%) as a pink solid.

^{1}H NMR (400 MHz, $CDCl_3$) δ 10.26 (s, 1H), 8.34 (dd, J = 8.5, 0.9 Hz, 1H), 8.22 (t, J = 1.1 Hz, 1H), 7.99 (dd, J = 8.5, 1.3 Hz, 1H), 7.27 – 7.22 (m, 2H), 6.89 – 6.84 (m, 2H), 5.66 (s, 2H), 3.97 (s, 3H), 3.77 (s, 3H) ppm.

Compound **S12**

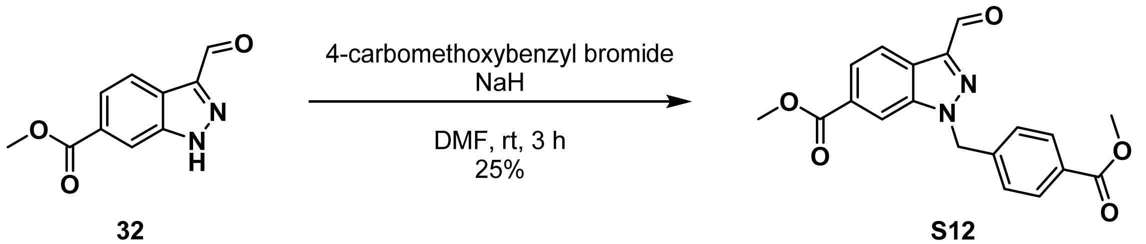

32 **S12**

methyl 3-formyl-1-(4-(methoxycarbonyl)benzyl)-1H-indazole-6-carboxylate (**S12**): To a suspension of sodium hydride (60% in oil, 241.0 mg, 6.02 mmol) in dimethylformamide (10 mL) was added indazole **32** (1.03 g, 5.02 mmol). This suspension was stirred for 30 min. 4-carbomethoxybenzyl bromide (246.0 mg, 1.07 mmol) was added and the reaction mixture was stirred for 3 h at room temperature. The excess of base was consumed through the addition of a solution of saturated ammonium chloride (10 mL). The reaction mixture was extracted with ethyl acetate (3 x 100 mL). The organic phase was washed with brine, dried over magnesium sulfate, filtered and concentrated *in vacuo*.

Purification by flash chromatography (petroleum ether/EtOAc 80:20 to 50/50) afforded the desired diester **S12** (445.5 mg, 1.26 mmol, 25%) as an off-white solid.

R_F 0.15 (petroleum ether/EtOAc 80/20).
^{1}H NMR (400 MHz, $CDCl_3$) δ 10.27 (s, 1H), 8.38 (dd, *J* = 8.5, 0.8 Hz, 1H), 8.16 (t, *J* = 1.1 Hz, 1H), 8.04 – 7.99 (m, 3H), 7.34 – 7.28 (m, 2H), 5.79 (s, 2H), 3.96 (s, 3H), 3.90 (s, 3H) ppm.

Compound **33**

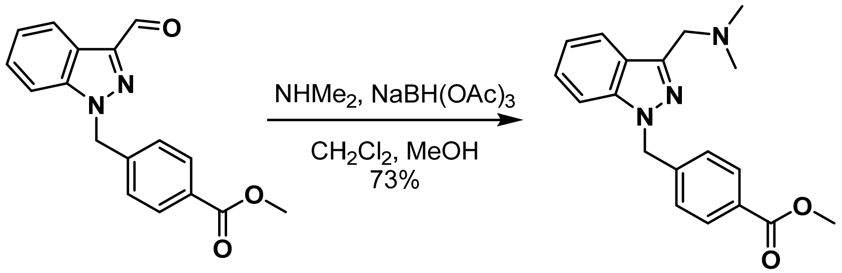

methyl 4-((3-((dimethylamino)methyl)-1H-indazol-1-yl)methyl)benzoate (**33**): To a solution of **S10** (227.8 mg, 774.0 μmol) in dichloromethane (20 mL) and dimethylamine (2.0 M in MeOH, 0.70 mL, 1.40 mmol) was added sodium triacetoxyborohydride (435.8 mg, 2.056 mmol) at 0 °C. Upon complete addition the cooling bath was removed and the reaction mixture was stirred for 48 h at room temperature. The reaction was stopped with the addition of dichloromethane (10 mL) and a solution of saturated sodium hydrogen carbonate (20 mL). The aqueous and organic layers were separated and the organic phase was washed with brine, dried over magnesium sulfate, filtered and concentrated *in vacuo*. Purification by flash chromatography (CH_2Cl_2/MeOH 90/10) afforded the desired ester **33** (182.2 mg, 563.4 μmol, 73%) as a yellow solid.

R_F 0.32 (CH_2Cl_2/MeOH 90/10).
^{1}H NMR (400 MHz, DMSO-d_6) δ 7.90 - 7.87 (m, 2H), 7.84 (d, *J* = 8.1 Hz, 1H), 7.63 (d, *J* = 8.4 Hz, 1H), 7.38 - 7.34 (m, 1H), 7.29 - 7.27 (m, 2H), 7.14 - 7.10 (m, 1H), 5.71 (s, 2H), 3.80 (s, 3H), 3.76 (s, 2H), 2.19 (s, 6H) ppm.
^{13}C NMR (100 MHz, DMSO-d_6) δ 165.9, 143.2, 124.5, 140.5, 129.4, 128.7, 127.3, 126.4, 123.0, 121.0, 120.2, 109.6, 55.2, 52.1, 51.1, 45.0 ppm.
LC-MS *m/z*: $[M + H]^+$ 324.

Compound **1g**

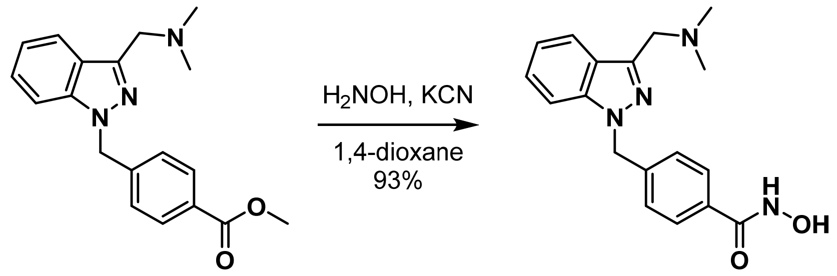

33 **1g**

4-((3-((dimethylamino)methyl)-1H-indazol-1-yl)methyl)-*N*-hydroxybenzamide (**1g**): To a solution of **33** (153.6 mg, 475.0 µmol) in 1,4-dioxane (10 mL) were added an aqueous solution of hydroxylamine (50 wt%, 10.0 mL) and potassium cyanide (65.3 mg, 1.003 mmol). The reaction mixture was stirred for 48 h at room temperature. The reaction was stopped with the removal of the excess of reagent and solvent *in vacuo*. Purification by HPLC afforded the TFA salt of the desired hydroxamic acid **1g** (193.2 mg, 0.44 mmol, 93%) as an orange oil.

R$_F$ 0.23 (CH_2Cl_2/MeOH 80/20).
^{1}H NMR (400 MHz, DMSO-d_6) δ 11.19 (s, 1H), 10.30 (s, 2H), 8.01 - 7.99 (m, 1H), 7.79 (d, *J* = 8.5 Hz, 1H), 7.69 - 7.67 (m, 2H), 7.47 (ddd, *J* = 1.0, 6.9, 8.6 Hz, 1H), 7.30 - 7.25 (m, 3H), 5.78 (s, 2H), 4,71 (s, 2H), 2.83 (s, 6H) ppm.
^{13}C NMR (101 MHz, DMSO d_6) δ 163.8, 158.5 (q, *J* = 31.8 Hz), 140.3, 140.1, 134.9, 132.2, 127.3, 127.2, 127.1, 123.4, 121.6, 120.4, 117.1 (q, *J* = 298.7 Hz), 110.3, 104.2, 51.7, 51.1, 42.1 ppm.
HRMS-ESI *m/z*: $[M + H]^+$ calcd for $C_{18}H_{21}N_4O_2$: 325.1659; found: 325.1659.

Compound **34**

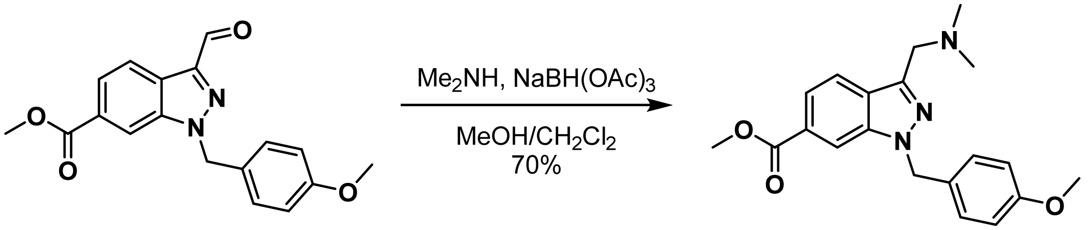

S11 **34**

methyl 3-((dimethylamino)methyl)-1-(4-methoxybenzyl)-1H-indazole-6-carboxylate (**34**): The aldehyde **S11** (0.35 g, 1.08 mmol) was dissolved in 20.0 mL of CH_2Cl_2 and treated with dimethylamine (2M in MeOH; 1.6 mL) and sodium triacetoxyborohydride (0.45 g, 2.19 mmol). The mixture was stirred at room temperature for 18 h. Afterwards the reaction mixture was diluted with ethyl acetate (100 mL) and washed with saturated $NaHCO_3$ solution (2 x 100 mL), dried over $MgSO_4$, filtered and concentrated. The crude material was purified by column chromatography (CH_2Cl_2/MeOH - 9:1) to afford a colorless oil as the pure product **34** (269.7 mg, 0.76 mmol, 70%).

R$_F$ 0.5 (CH_2Cl_2/MeOH 9/1).
^{1}H NMR (400 MHz, $CDCl_3$) δ 8.09 (t, *J* = 1.1 Hz, 1H), 7.86 (dd, *J* = 8.5, 0.9 Hz, 1H), 7.77 (dd, *J* = 8.5, 1.3 Hz, 1H), 7.20 – 7.13 (m, 2H), 6.86 – 6.78 (m, 2H), 5.56 (s, 2H), 3.96 – 3.92 (m, 3H), 3.85 (s, 2H), 3.79 – 3.73 (m, 3H), 2.32 (s, 6H) ppm.

Compound **2g**

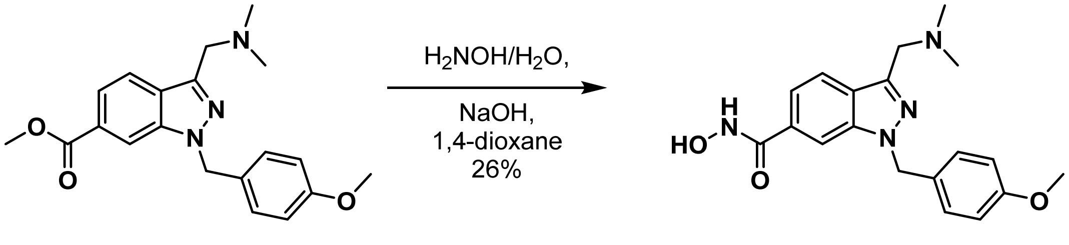

34 **2g**

3-((dimethylamino)methyl)-*N*-hydroxy-1-(4-methoxybenzyl)-1H-indazole-6-carboxamide (**2g**): The ester **34** (269.7 mg, 0.76 mmol) was dissolved in 1.0 mL of 1,4-dioxane and treated with a prepared mixture of an aqueous solution of hydroxylamine (50 wt%, 1.50 mL) and sodium hydroxide (152.6 mg, 3.82 mmol). The resulting reaction mixture was stirred at room temperature for 18 h and then concentrated on a rotary evaporator. This material was subjected to column chromatography (CH_2Cl_2/MeOH 9/1) to afford a white solid as the pure product **2g** (68.8 mg, 0.19 mmol, 26%).

R$_F$ 0.08 (CH_2Cl_2/MeOH 9/1).
^{1}H NMR (400 MHz, DMSO-*d$_6$*) δ 11.26 (s, 1H), 9.08 (s, 1H), 8.10 – 8.03 (m, 1H), 7.86 (dd, *J* = 8.5, 0.8 Hz, 1H), 7.48 (dd, *J* = 8.5, 1.3 Hz, 1H), 7.22 – 7.17 (m, 2H), 6.89 – 6.83 (m, 2H), 5.56 (s, 2H), 3.76 (s, 2H), 3.69 (s, 3H), 2.18 (s, 6H) ppm.
^{13}C NMR (100 MHz, DMSO-*d$_6$*) δ 158.7, 142.1, 139.6, 130.8, 129.4, 128.6, 124.4, 121.0, 118.6, 113.9, 108.9, 55.1, 55.0, 51.2, 45.0 ppm.
HRMS-ESI *m/z*: $[M + H]^+$ calcd for $C_{19}H_{23}N_4O_3$: 355.1765; found: 355.1765.

Compound **35**

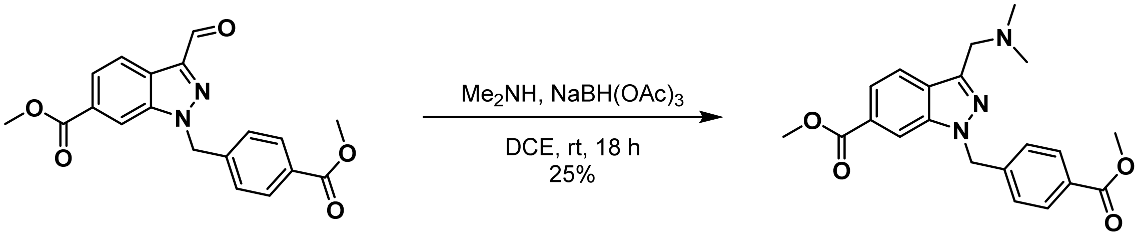

S12 **35**

methyl 3-((dimethylamino)methyl)-1-(4-(methoxycarbonyl)benzyl)-1H-indazole-6-carboxylate (**35**): The aldehyde **S12** (428.5 mg, 1.22 mmol) was dissolved in 20.0 mL of DCE and treated with dimethylamine (2M in MeOH; 1.83 mL, 3.66 mmol) and sodium triacetoxyborohydride (517.2 mg, 2.44 mmol). The mixture was stirred at room temperature for 18 h. Afterwards the reaction mixture was diluted with ethyl acetate (100 mL) and washed with saturated $NaHCO_3$ solution (2 x 100 mL), dried over $MgSO_4$, filtered and concentrated. The crude material was purified by column chromatography (CH_2Cl_2/MeOH 9/1) to afford a colorless oil as the pure product **35** (118.7 mg, 0.31 mmol, 25%).

R$_F$ 0.5 (CH_2Cl_2/MeOH 90/10).
^{1}H NMR (400 MHz, $CDCl_3$) δ 8.05 (dd, *J* = 1.3, 0.8 Hz, 1H), 7.97 – 7.93 (m, 2H), 7.90 (dd, *J* = 8.5, 0.8 Hz, 1H), 7.81 (dd, *J* = 8.5, 1.3 Hz, 1H), 7.22 – 7.18 (m, 2H), 5.67 (s, 2H), 3.93 (s, 3H), 3.88 (s, 3H), 3.87 (s, 2H), 2.33 (s, 6H) ppm.

^{13}C NMR (100 MHz, $CDCl_3$) δ 167.3, 166.8, 143.4, 141.8, 140.3, 130.2, 129.9, 128.6, 127.0, 126.4, 121.3, 121.2, 111.5, 55.8, 52.6, 52.5, 52.3, 45.7 ppm.

Compound **3g**

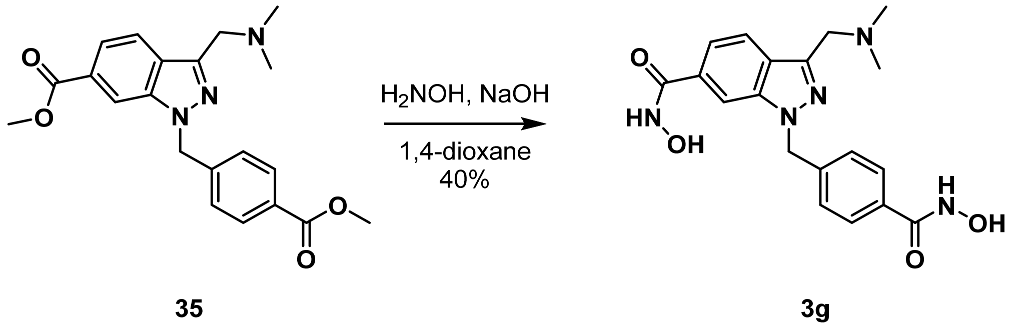

3-((dimethylamino)methyl)-*N*-hydroxy-1-(4-(hydroxycarbamoyl)benzyl)-1H-indazole-6-carboxamide (**3g**): The ester **35** (109 mg, 0.29 mmol) was dissolved in 2.0 mL of 1,4-dioxane and treated with a prepared mixture of an aqueous solution of hydroxylamine (50 wt%, 2.0 mL) and sodium hydroxide (114.3 mg, 2.86 mmol). The resulting reaction mixture was stirred at room temperature for 18 h and then concentrated on a rotary evaporator. This material was purified by HPLC to afford a white solid as the TFA salt of **3g** (52.3 mg, 0.11 mmol, 40%).

^{1}H NMR (400 MHz, DMSO-d_6) δ 11.36 (s, 1H), 11.17 (s, 1H), 10.13 (s, 1H), 9.07 (br. s, 1H), 9.03 (br.s, 1H), 8.21 (d, J = 1.3 Hz, 1H), 8.06 (dd, J = 8.5, 0.8 Hz, 1H), 7.72 – 7.67 (m, 2H), 7.64 (dd, J = 8.6, 1.3 Hz, 1H), 7.33 – 7.28 (m, 2H), 5.82 (s, 2H), 4.71 (s, 2H), 2.83 (s, 6H) ppm.
^{13}C NMR (100 MHz, DMSO-d_6) δ 164.0, 163.8, 157.9 (q, J = 31.4 Hz), 139.9, 135.1, 132.3, 131.7, 127.3, 124.7, 120.5, 120.0, 117.1 (q, J = 299.5 Hz), 109.5, 51.8, 51.1, 42.1 ppm.
HRMS-ESI *m/z*: $[2M + K]^+$ calcd for $C_{38}H_{42}KN_{10}O_8$: 805.2819; found: 805.3535.

Synthesis of DTBTA-Eu^{3+}-labelled Streptactin: To a solution of ATBTA-Eu^{3+} (39 mM; 60 µL, 2.3 µmol) from TCI, cat#A2083 in aqueous NaOAc (100 mM, pH 4.9) was added a solution of cyanuric chloride (93 mM in acetone; 25 µL, 2.3 µmol), then vortexed and mixed on a tube roller mixer at room temperature for 30 min. The solution was added dropwise to acetone (1 mL), the resulting suspension was centrifuged (10 000 rpm, 3 min), the precipitate was washed (3x) by resuspending in acetone (1 mL each time), followed by centrifugation, then air dried at 37 °C for 1 h. The precipitate was redissolved in sodium carbonate buffer (100 mM, pH 9.3; 400 µL). To 100 µL of this solution was added a solution of Strep-Tactin XT (5.0 mg, gift from IBA Life Sciences) in sodium carbonate buffer (100 mM, pH 9.3; 400 µL), then vortexed and mixed with a rotary mixer at 4 °C overnight. The reaction mixture was purified with a PD10 desalting column (17-0851-01, GE Healthcare), pre-saturated with BSA, using TBS buffer (50 mM Tris/HCl pH 7.5, 150 mM NaCl, 0.01% NaN_3). Concentration and labelling ratio of the product fraction was determined with a NanoDrop (ThermoFisher) spectrophotometer.

Synthesis of FRET tracer **S15**[7]

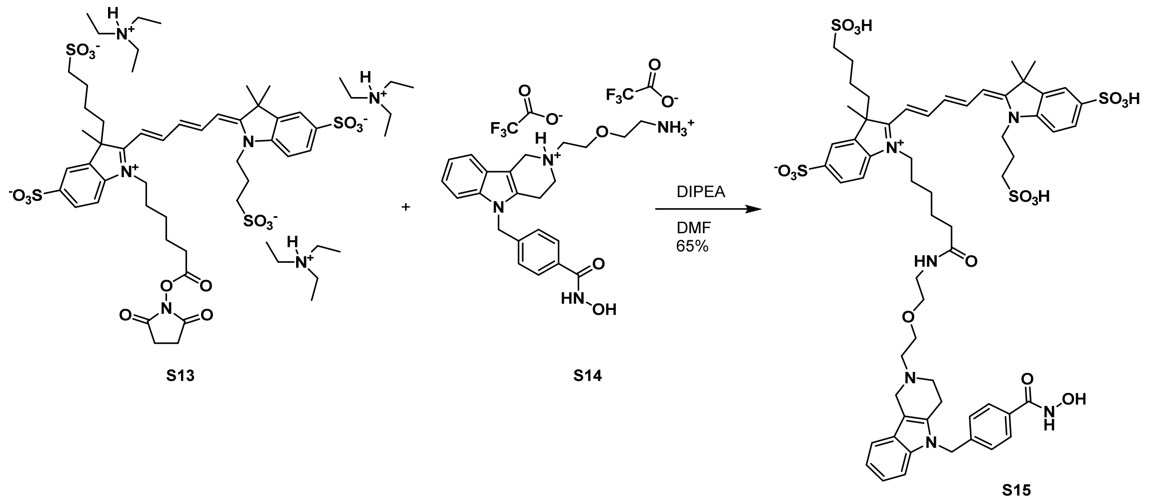

AF647 tubastatin A tracer (**S15**): AF647 NHS ester **S13** (5.0 mg, 3.93 µmol, 1 equiv.) from Fluoroprobes, cat#1121-1, lot#10022 was mixed with **S14**[7] (10.0 mg, 16µmol, 4 equiv.) in 1.5 mL of DMF. To this solution was added *i*Pr_2NEt (5 µL, 27.0 µmol, 7 equiv). After stirring at room temperature for 30 minutes the solution was concentrated and then purified by HPLC under basic conditions with a gradient of 1 – 40% acetonitrile over 18 min to afford **S15** (3.5 mg, 2.54 µmol, 65%) as a dark blue solid.

LC-MS *m/z*: $[(M-2H)/2]^{2-}$ 630.2.

Figure S1. Dose-response curves of Tubastatin A, PCI-34051, and their combination

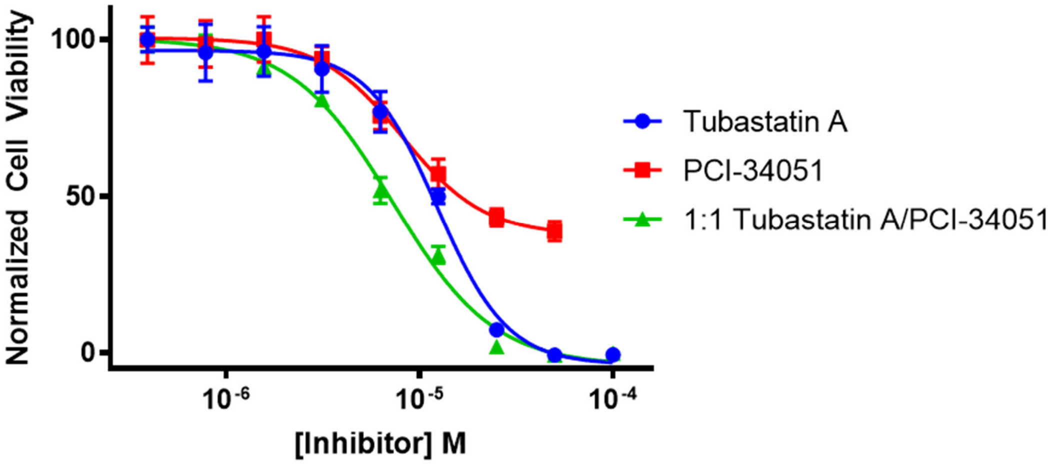

Dose-response curves of PCI-34051 (red), Tubastatin A (blue) and a 1:1 molar ratio of the two substances (green). PCI-34051 alone is not fully cytotoxic, but arrests cell growth, with an EC_{50} = 8.1 µM and a maximum effect of 62% cell growth inhibition. Tubastatin A is fully cytotoxic with an EC_{50} = 12.3 µM. The combination treatment is also fully cytotoxic and gives an improved EC_{50} = 7.3 µM.

Table S1. Enzyme inhibition values with error calculations

Cmpd	pIC50[a]					
	HDAC1	HDAC2	HDAC3	HDAC6	HDAC8	HDAC10
**1a**	--	--	--	--	6.42 (5.97–6.87)	7.18 ± 0.06 (n = 5)
**PCI–34051 (2a)**	4.33 (4.07–4.59)	<4.0 (No fit)	4.24 (4.18–4.29)	5.15 (5.06–5.24)	7.29 (7.24–7.34)	4.38 ± 0.03 (n = 3)
**3a**	6.58 (6.57–6.60)	5.65 (5.61–5.68)	5.26 (No fit–5.32)	7.74 (7.63–7.85)	7.17 (7.07–7.27)	8.59 (8.52–8.60)
**1b**	--	--	--	--	6.02 (5.95–6.09)	8.28 ± 0.03 (n = 2)
**2b**	--	--	--	--	7.31 (7.27–7.36)	5.79 ± 0.02 (n = 4)
**3b**	6.21 (6.16–6.25)	5.27 (5.24–5.30)	5.16 (very wide)	7.12 (6.99–7.26)	6.78 (6.74–6.83)	8.55 (8.47–8.64)
**Tubastatin A (1c)**	5.91 (5.86–5.96)	4.89 (4.79–4.94)	5.78 (5.70–5.86)	7.70 (7.62–7.77)	5.70 (5.53–5.87)	7.90 ± 0.04 (n = 5)
**2c**	--	--	--	--	6.85 (6.78–6.92)	5.19 ± 0.04 (n = 4)
**3c**	6.30 (6.29–6.32)	5.38 (5.35–5.42)	4.87 (4.75–No fit)	7.32 (7.17–7.48)	6.29 (6.21–6.37)	7.91 (7.82–8.01)
**1d**	--	--	--	--	--	7.56 ± 0.05 (n =4)
**2d**	--	--	--	--	7.16 (7.10–7.21)	5.50 ± 0.06 (n = 4)
**3d**	6.28 (6.26–6.30)	5.33 (5.27–5.38)	5.19 (very wide)	7.65 (7.56–7.75)	7.01 (6.98–7.04)	8.49 (8.37–8.61)
**2e**	--	--	--	--	7.36 (7.29–7.43)	6.18 ± 0.02 (n = 4)
**3e**	6.09 (6.08–6.11)	5.15 (5.09–5.21)	5.18 (very wide)	7.35 (7.26–7.43)	7.18 (7.13–7.23)	8.52 (8.45–8.60)
**1f**	--	--	--	--	6.41 (6.35–6.47)	6.63 ± 0.02 (n = 4)
**2f**	--	--	--	--	7.68 (7.60–7.76)	4.72 ± 0.01 (n = 4)
**3f**	7.06 (7.03–7.09)	6.14 (6.10–6.17)	5.88 (5.82–5.94)	7.82 (7.67–7.97)	7.37 (7.33–7.40)	8.47 (8.33–8.64)
**1g**	--	--	--	--	6.07 (6.00–6.13)	7.71 ± 0.02 (n = 2)
**2g**	--	--	--	--	7.53 (7.48–7.59)	5.16 ± 0.04 (n = 4)
**3g**	6.18 (6.14–6.21)	5.02 (4.87–No fit)	5.18 (very wide)	7.52 (7.45–7.60)	7.05 (7.00–7.09)	8.40 (7.97–10.65)

[a] Values in parenthesis are 95% confidence intervals, determined from a 4-parameter dose-response non-linear regression. Values reflect one experiment measured in triplicate. Plus/minus values are the standard deviation of the arithmetic mean of pIC_{50} values determined in the number of experiments (n) performed as shown in parenthesis.

Table S2. Crystallographic data collection and refinement statistics for the HDAC10-3a complex

**Data Collection**	
Space group	$P3_121$
a,b,c (Å)	80.6, 80.6, 245.4
α,β,γ (°)	90 90 120
R_{merge}[b]	0.109 (1.349)[a]
R_{pim}[c]	0.038 (0.478)[a]
$CC_{1/2}$[d]	0.998 (0.591)[a]
Redundancy	9.0 (8.8)[a]
Completeness (%)	100 (100)[a]
I/σ	11.7 (2.0)[a]
**Refinement**	
Resolution (Å)	69.76-2.05 (2.12-2.05)[a]
No. reflections	58990 (11619)[a]
R_{work}/R_{free}[e]	0.193/ 0.232 (0.298/0.337)[a]
**No. atoms**[f]	
Protein	4880
Ligand	69
Solvent	261
**Average B factors (Å^2)**	
Protein	43
Ligand	42
Solvent	43
**R.m.s. deviations**	
Bond lengths (Å)	0.008
Bond angles (°)	1.0
**Ramachandran plot**[g]	
Favored	95.4
Allowed	4.29
Outliers	0.32
PDB accession code	6VNQ

[a]Values in parentheses refer to the highest-resolution shell indicated. [b]$R_{merge} = \sum_{hkl}\sum_i|I_{i,hkl} - \langle I\rangle_{hkl}|/\sum_{hkl}\sum_i I_{i,hkl}$, where $\langle I\rangle_{hkl}$ is the average intensity calculated for reflection *hkl* from replicate measurements. Note that aberrantly high R_{merge} values can result for highly redundant data sets, in which case $R_{p.i.m.}$ serves as a better indicator of data quality. [c]$R_{p.i.m.} = (\sum_{hkl}(1/(N-1))^{1/2}\sum_i|I_{i,hkl} - \langle I\rangle_{hkl}|)/\sum_{hkl}\sum_i I_{i,hkl}$, where $\langle I\rangle_{hkl}$ is the average intensity calculated for reflection *hkl* from replicate measurements and N is the number of reflections. [d]Pearson correlation coefficient between random half-datasets. [e]$R_{work} = \sum||F_o| - |F_c||/\sum|F_o|$ for reflections contained in the working set. $|F_o|$ and $|F_c|$ are the observed and calculated structure factor amplitudes, respectively. R_{free} is calculated using the same expression for reflections contained in the test set held aside during refinement. [f]Per asymmetric unit. [g]Calculated with MolProbity.

Table S3. Crystallographic data collection and refinement statistics for the HDAC6-3a complex

**Data Collection**	
Space group	$P2_1$
a,b,c (Å)	48.4, 107.1, 74.4
α,β,γ (°)	90 90 90
R_{merge}[b]	0.136 (0.492)[a]
R_{pim}[c]	0.131 (0.468)[a]
$CC_{1/2}$[d]	0.912 (0.518)[a]
Redundancy	1.7 (1.7)[a]
Completeness (%)	88.5 (90.8)[a]
I/σ	4.8 (2.3)[a]
**Refinement**	
Resolution (Å)	44.09-1.94 (2.01-1.94)[a]
No. reflections	49179 (5063)[a]
R_{work}/R_{free}[e]	0.163/ 0.212 (0.207/0.270)[a]
**No. atoms[f]**	
Protein	5480
Ligand	62
Solvent	448
**Average B factors (Å^2)**	
Protein	10
Ligand	21
Solvent	15
**R.m.s. deviations**	
Bond lengths (Å)	0.006
Bond angles (°)	0.8
**Ramachandran plot[g]**	
Favored	97.0
Allowed	3.00
Outliers	0.00
PDB accession code	6VNR

[a]Values in parentheses refer to the highest-resolution shell indicated. [b]$R_{merge} = \sum_{hkl}\sum_i|I_{i,hkl} - \langle I\rangle_{hkl}|/\sum_{hkl}\sum_i I_{i,hkl}$, where $\langle I\rangle_{hkl}$ is the average intensity calculated for reflection *hkl* from replicate measurements. [c]$R_{p.i.m.} = (\sum_{hkl}(1/(N-1))^{1/2}\sum_i|I_{i,hkl} - \langle I\rangle_{hkl}|)/\sum_{hkl}\sum_i I_{i,hkl}$, where $\langle I\rangle_{hkl}$ is the average intensity calculated for reflection *hkl* from replicate measurements and N is the number of reflections. [d]Pearson correlation coefficient between random half-datasets. [e]$R_{work} = \sum||F_o| - |F_c||/\sum|F_o|$ for reflections contained in the working set. $|F_o|$ and $|F_c|$ are the observed and calculated structure factor amplitudes, respectively. R_{free} is calculated using the same expression for reflections contained in the test set held aside during refinement. [f]Per asymmetric unit. [g]Calculated with MolProbity.

Table S4. Prediction of log P values of synthesized inhibitors

1: X = H; Y = C(O)NHOH
2: X = C(O)NHOH; Y = OMe
3: X = Y = C(O)NHOH

Scaffold	Cmpd.	log P[a]
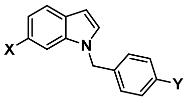	**1a**	2.62
	**2a**	2.49
	**3a**	1.69
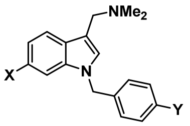	**1b**	2.56
	**2b**	2.43
	**3b**	1.63
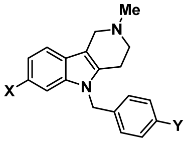	**1c**	2.42
	**2c**	2.29
	**3c**	1.49
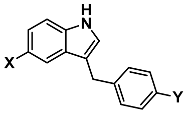	**1d**	2.74
	**2d**	2.61
	**3d**	1.81
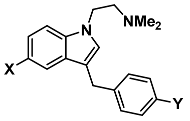	**2e**	2.85
	**3e**	2.05
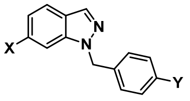	**1f**	2.23
	**2f**	2.10
	**3f**	1.30
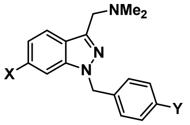	**1g**	2.59
	**2g**	2.47
	**3g**	1.67

[a] values predicted with ChemDraw, Version 16.0.1.4

References

1. Buggy, J. J.; Balasubramanian, S.; Verner, E.; Tai, V. W. F.; Lee, C.-S. Indole Derivatives as Inhibitors of Histone Deacetylase. US 2013/0156727 A1, 2013.

2. De Rosa, M.; Soriente, A. Rapid and General Protocol towards Catalyst-Free Friedel-Crafts C-Alkylation of Indoles in Water Assisted by Microwave Irradiation. *Eur. J. Org. Chem.* **2010**, 1029-1032.

3. Lee, C.; Yang, H.-M.; Kim, D.; Bae, M.; Choi, Y.; Ha, N. Novel Indole Derivative Compound and Pharmaceutical Composition Comrpising the same. WO 2015/102426 A1, 2015.

4. Jacobs, R. T.; Costello, G. F.; Brook, S. A.; Harrison, P. J. Chemical Process for the Preparation of 3-Alkylated Indole. US5280125A, 1994.

5. Corey, E. J.; Gilman, N. W.; Ganem, B. E. New methods for the oxidation of aldehydes to carboxylic acids and esters. *J. Am. Chem. Soc.* **1968,** *90*, 5616-5617.

6. Cummings, G.; Patel, N. K. B.; Forrest, B. T.; Liu, Y.; Li, S.-W.; Sampson, P. B.; Edwards, L. Synthesis of chiral 2-(1H-indazol-6-yl)-spiro[cyclopropane-1,3-indolin]-2'-ones. WO 2011/123947 A1, 2011.

7. Géraldy, M.; Morgen, M.; Sehr, P.; Steimbach, R.; Ridinger, J.; Oehme, I.; Witt, O.; Nogueira, M. S.; Koch, O.; Gunkel, N.; Miller, A. K.; Malz, M. Selective Inhibition of Histone Deacetylase 10: Hydrogen Bonding to the Gatekeeper Residue is Implicated. *J. Med. Chem.* **2019**, *62*, 4426-4443.

NMR Spectra

Bishydroxamic acid 3a, 1H, DMSO-d6

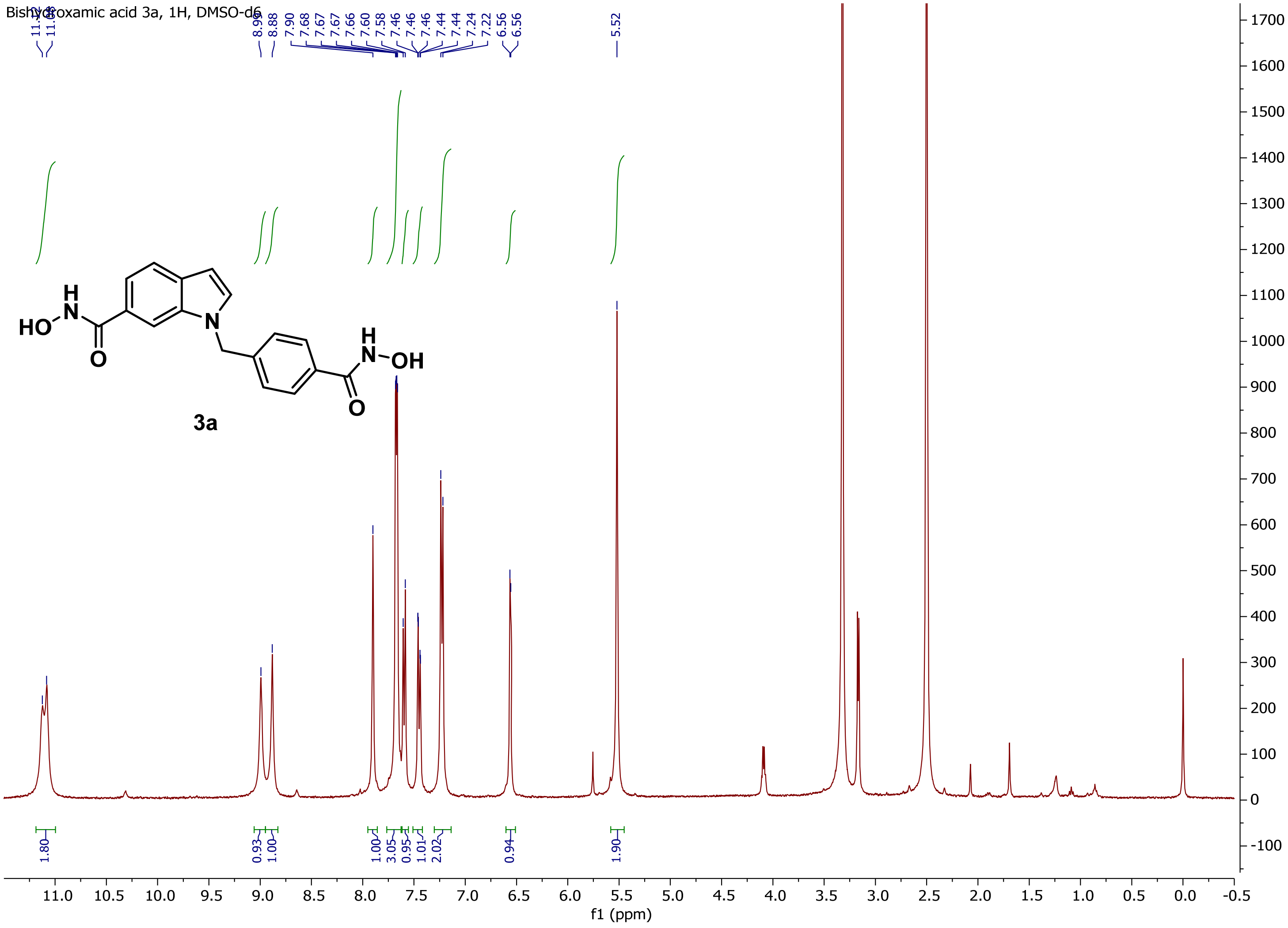

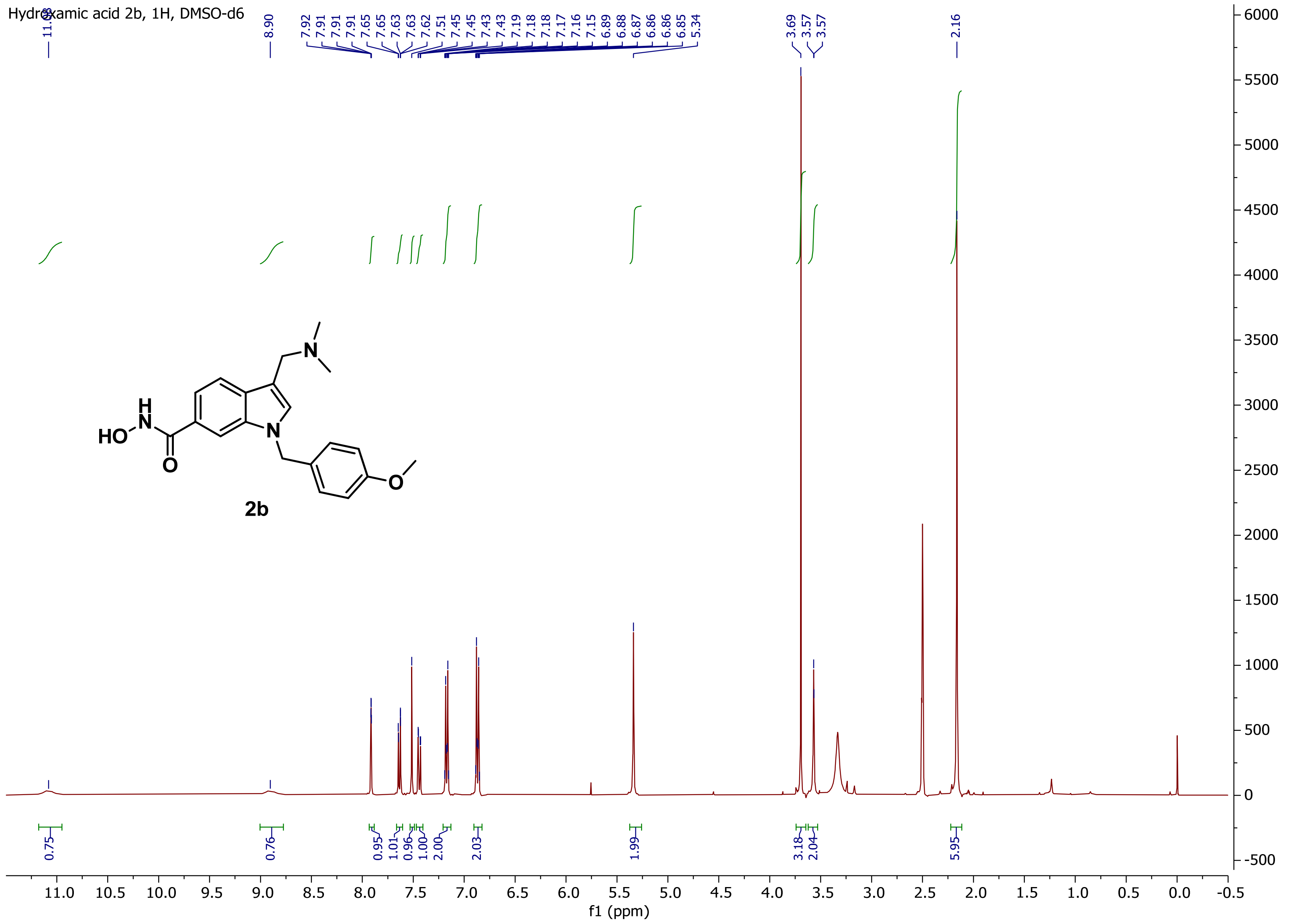

Hydroxamic acid 2b, 1H, DMSO-d6
2b
HO
H
N
O
N
N
O
f1 (ppm)

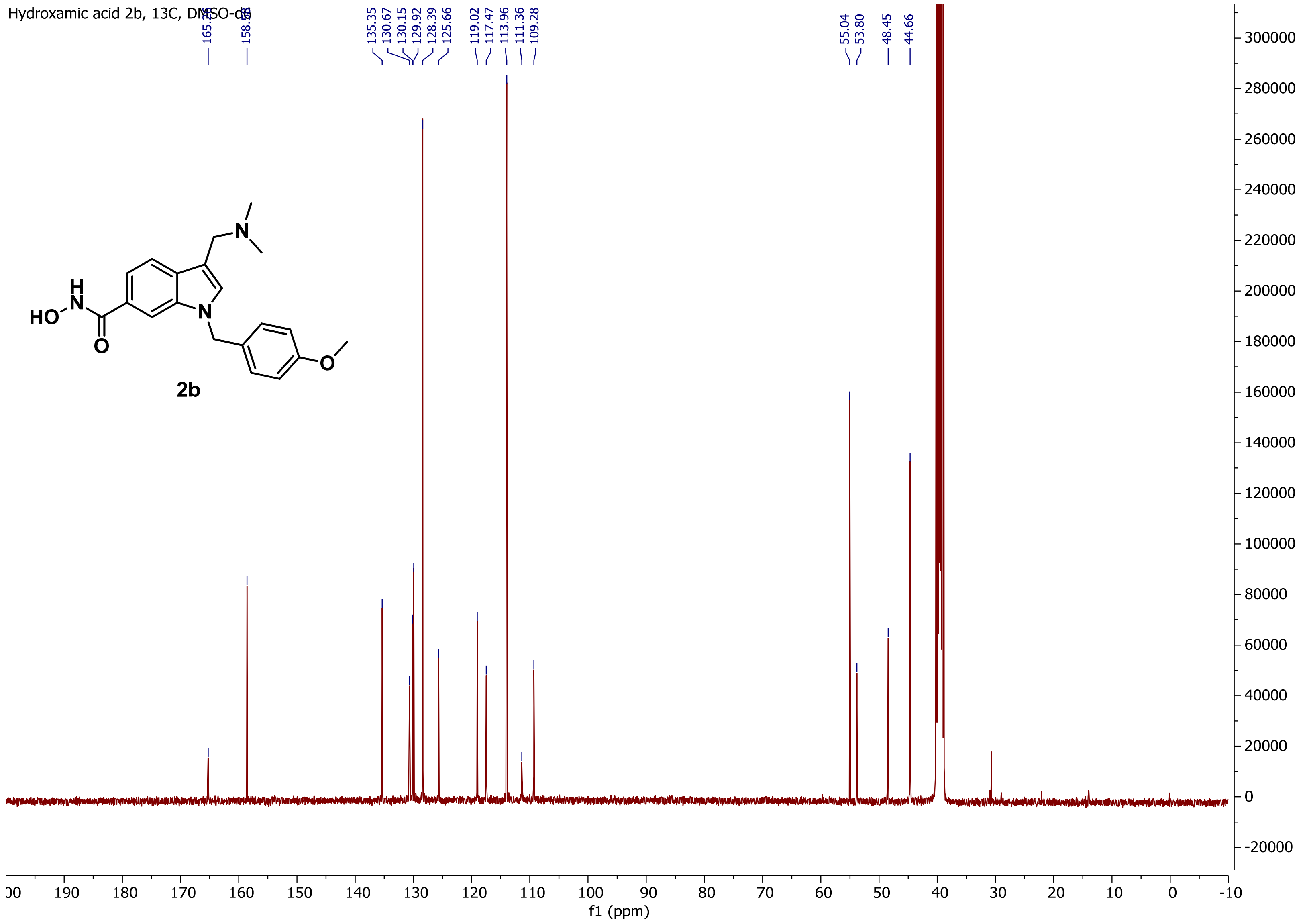
Hydroxamic acid 2b, 13C, DMSO-d6
2b
135.35
130.67
130.15
129.92
128.39
125.66
119.02
117.47
113.96
111.36
109.28
55.04
53.80
48.45
44.66
f1 (ppm)

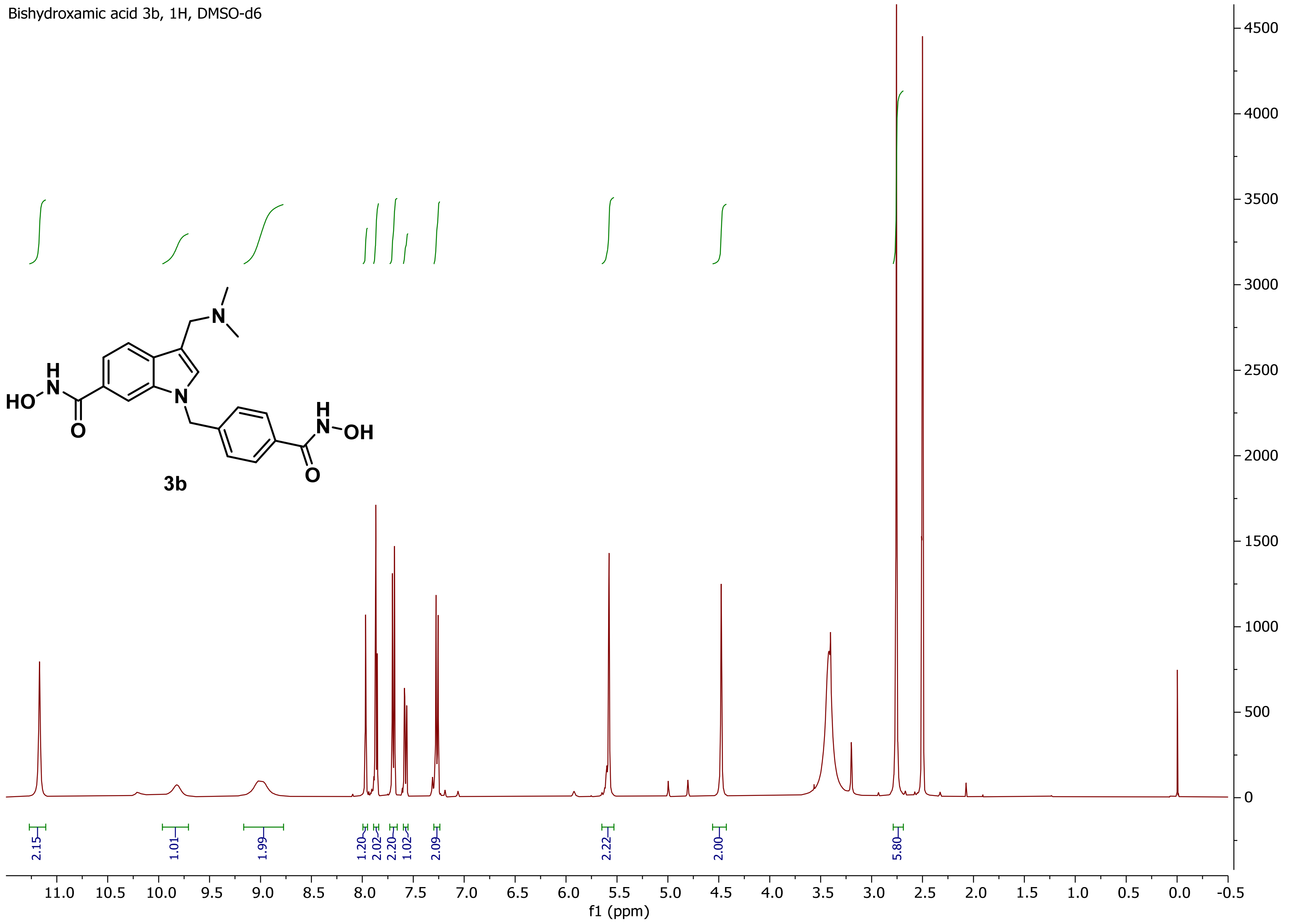
Bishydroxamic acid 3b, 1H, DMSO-d6
3b
2.15
1.01
1.99
1.20
2.02
2.20
1.02
2.09
2.22
2.00
5.80
f1 (ppm)

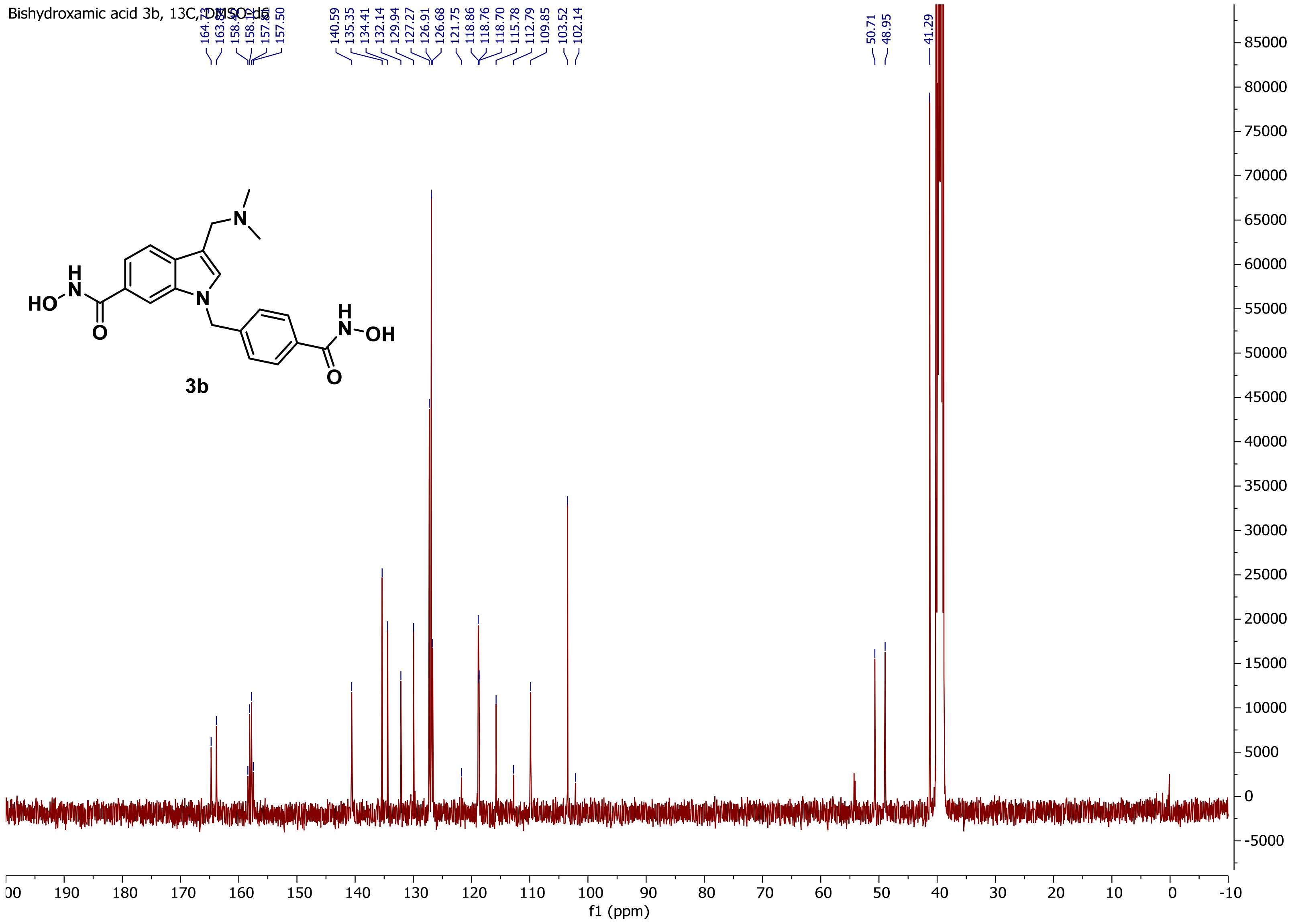
Bishydroxamic acid 3b, 13C, DMSO-d6
164.73
163.8
158.4
158.1
157.8
157.50
140.59
135.35
134.41
132.14
129.94
127.27
126.91
126.68
121.75
118.86
118.76
118.70
115.78
112.79
109.85
103.52
102.14
50.71
48.95
41.29
3b
f1 (ppm)

Hydroxamic acid 2c, 1H, DMSO-d6

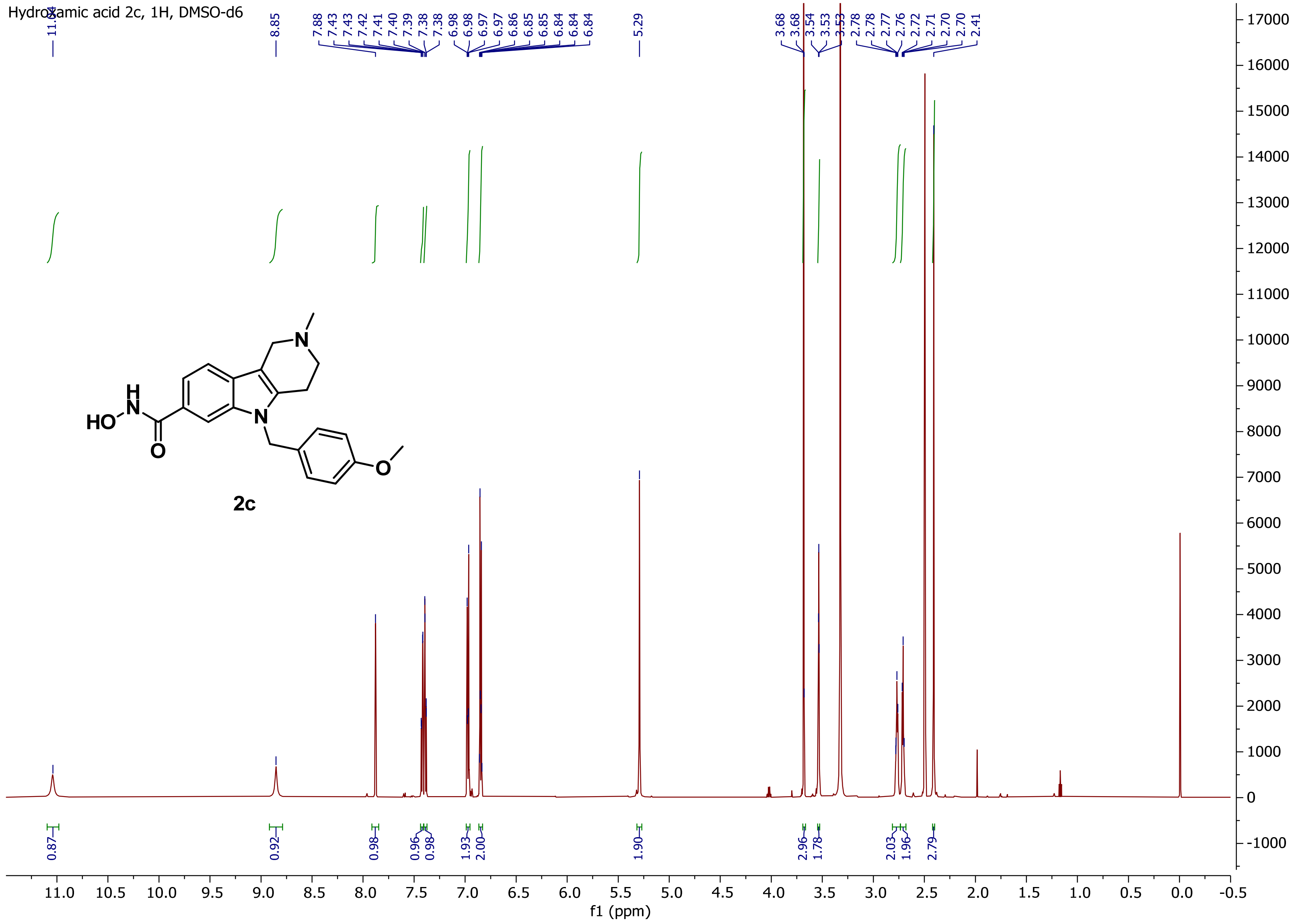

2c

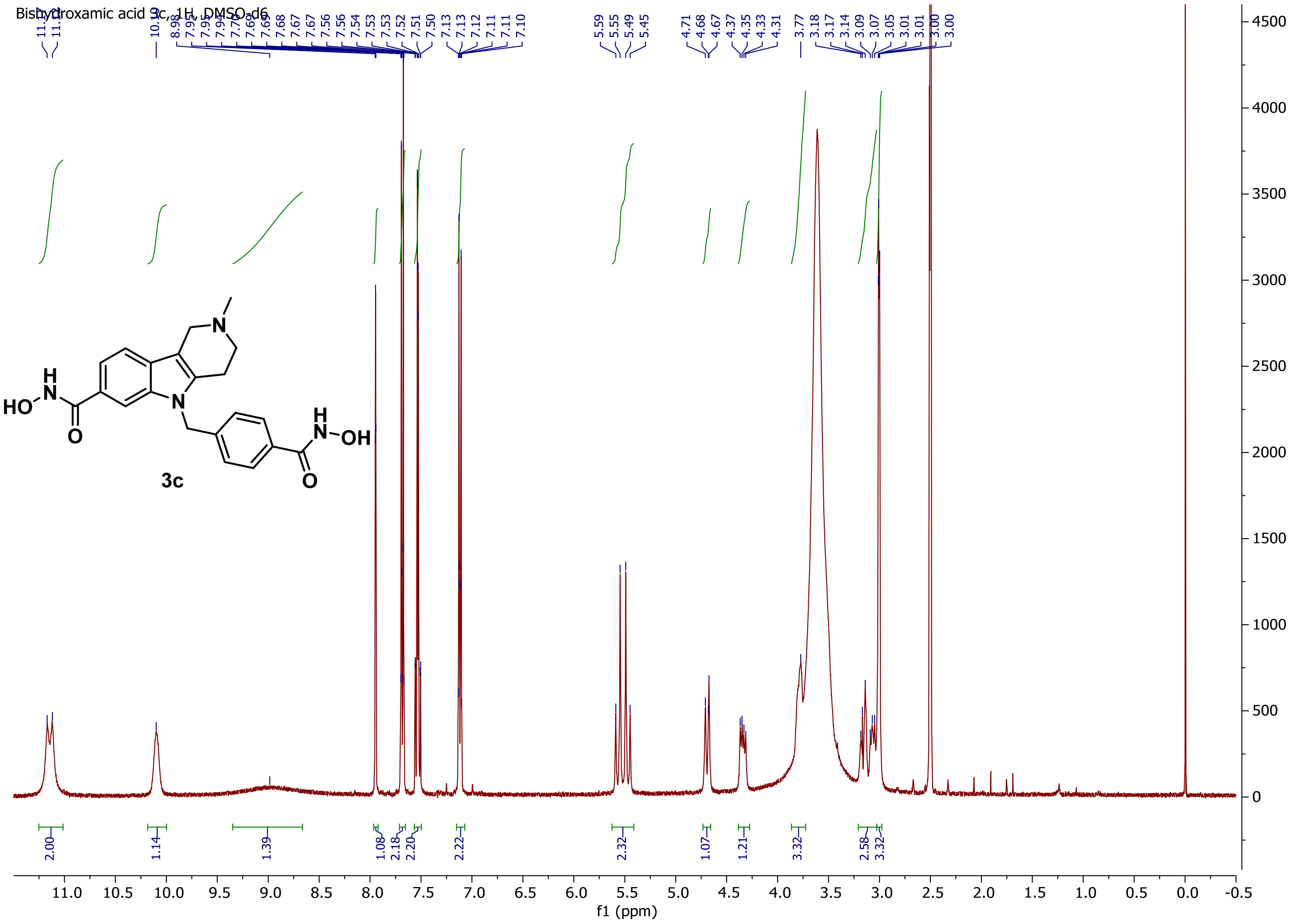
Bishydroxamic acid 3c, 1H, DMSO-d6
3c
f1 (ppm)

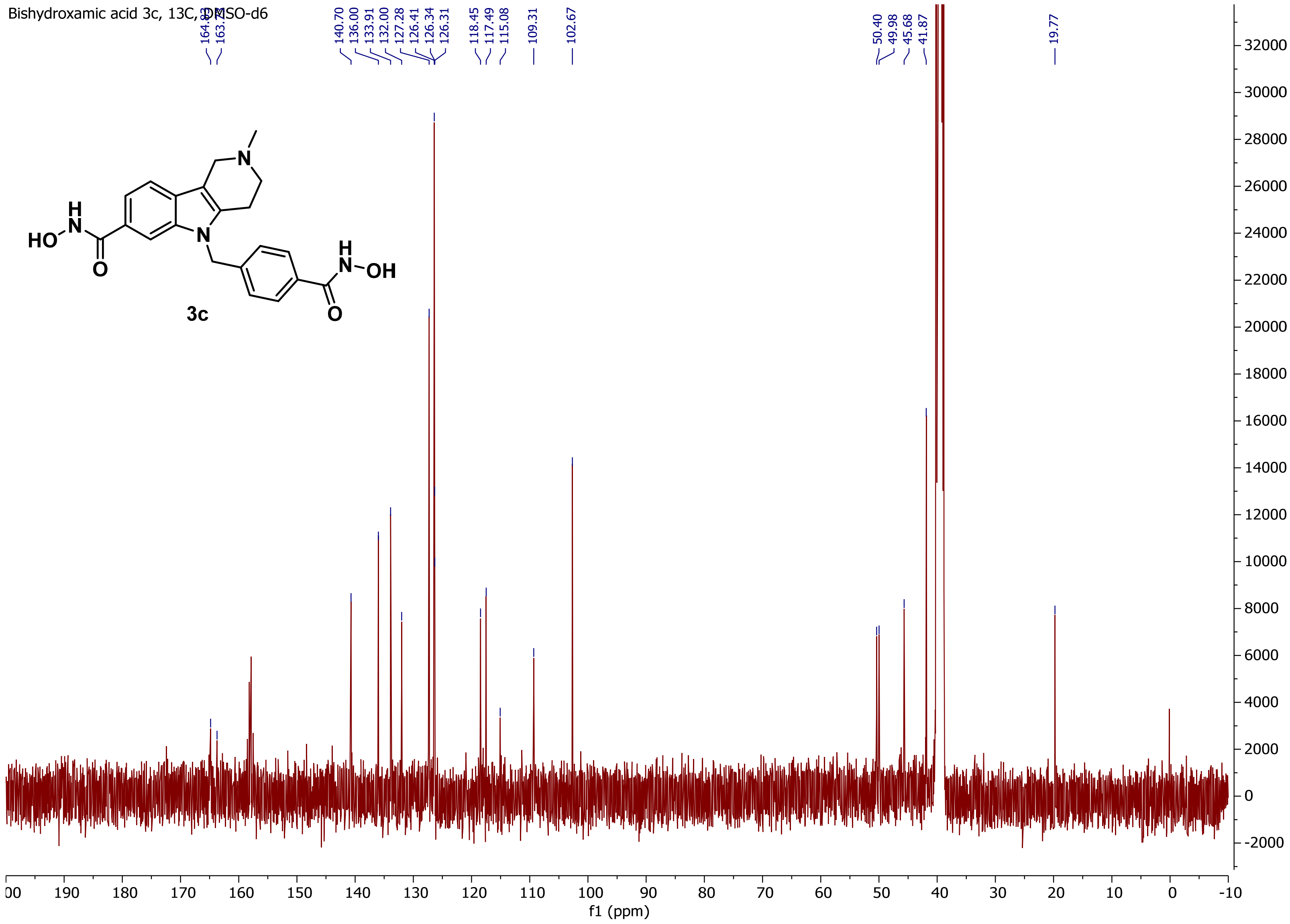
Bishydroxamic acid 3c, 13C, DMSO-d6
164.83
163.72
140.70
136.00
133.91
132.00
127.28
126.41
126.34
126.31
118.45
117.49
115.08
109.31
102.67
50.40
49.98
45.68
41.87
19.77
HO
H
N
O
N
N
H
N
OH
O
3c
f1 (ppm)

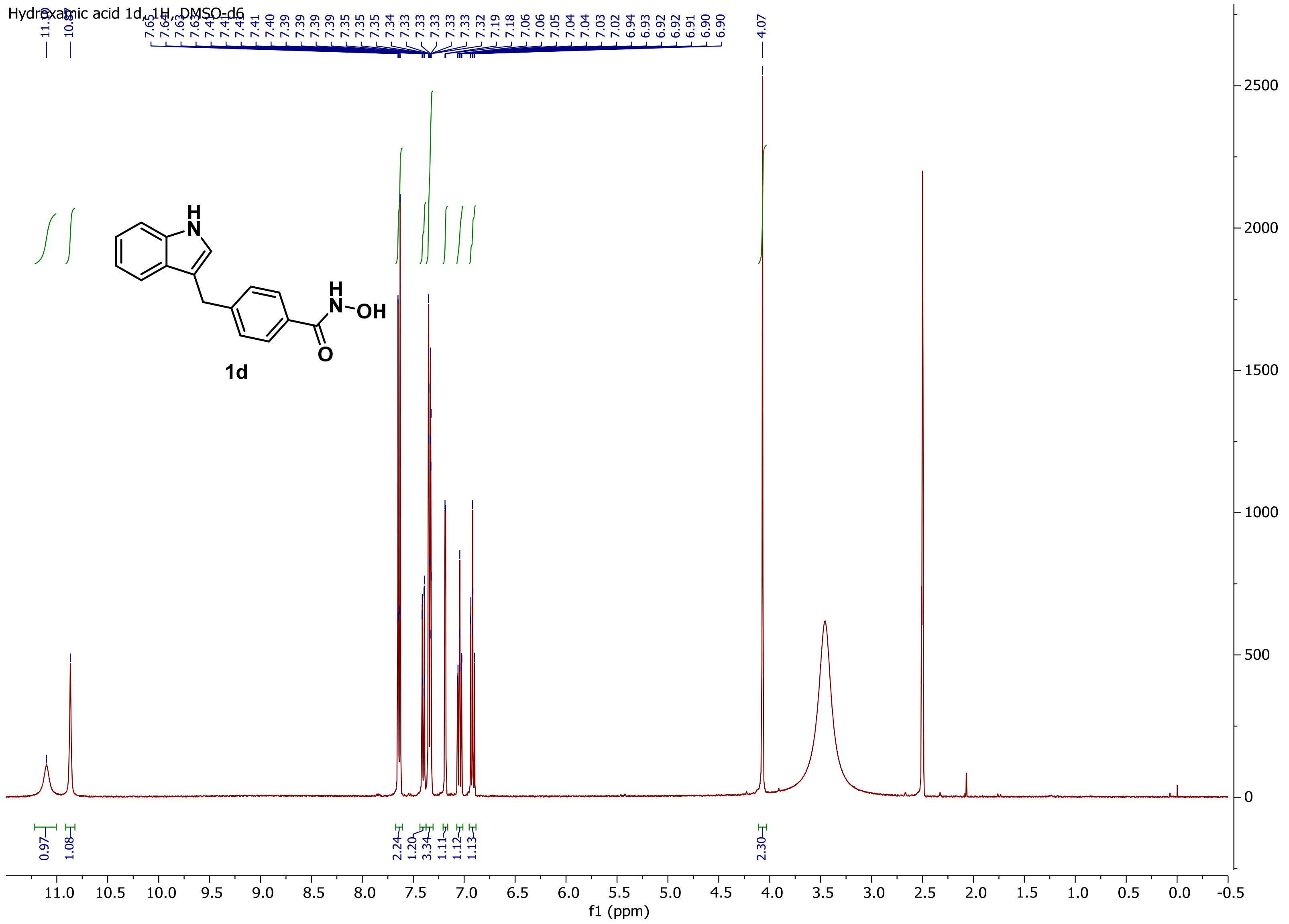
Hydroxamic acid 1d, 1H, DMSO-d6
1d
f1 (ppm)

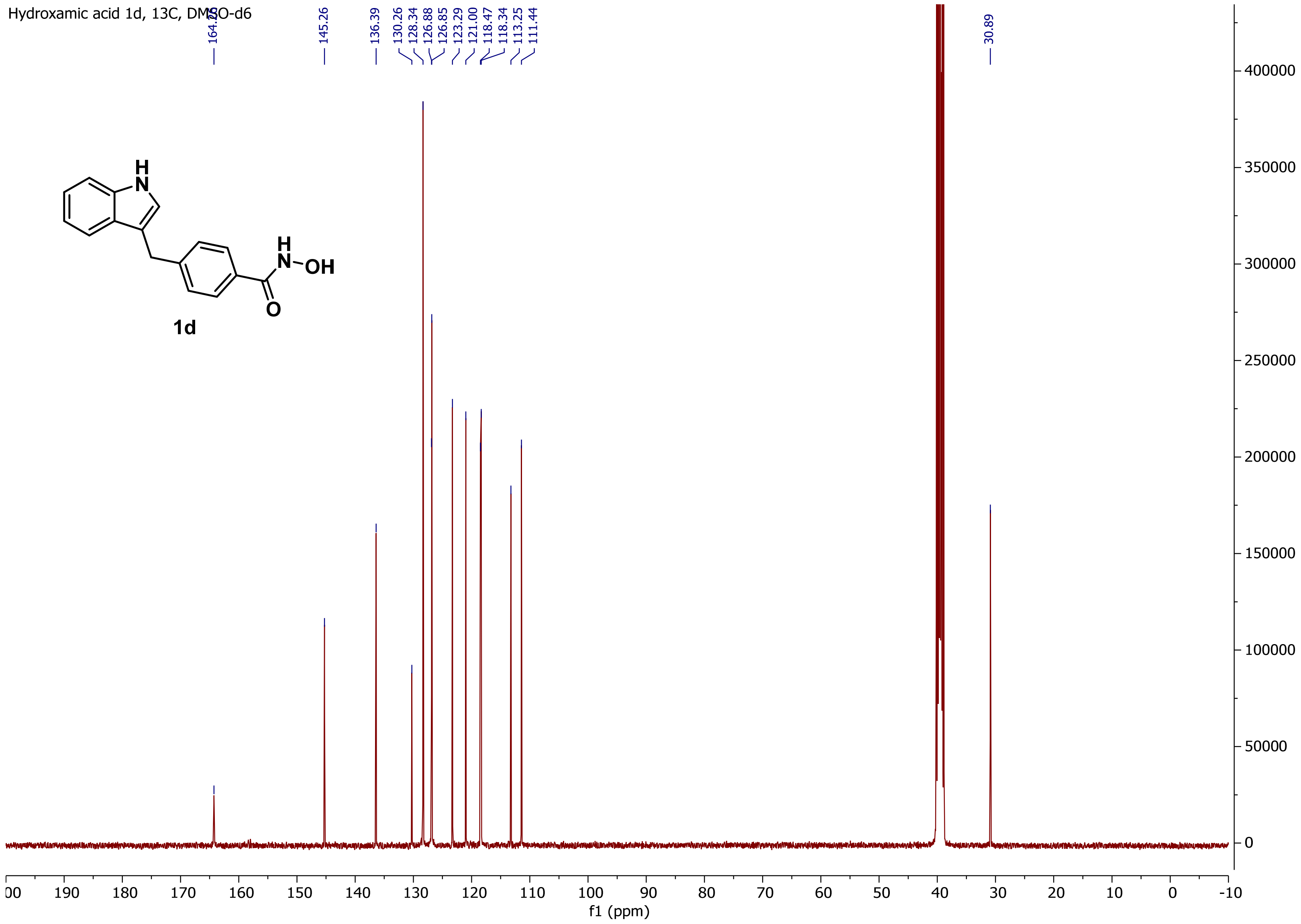
Hydroxamic acid 1d, 13C, DMSO-d6
164.20
145.26
136.39
130.26
128.34
126.88
126.85
123.29
121.00
118.47
118.34
113.25
111.44
30.89
H
N
H
N
OH
O
1d
f1 (ppm)

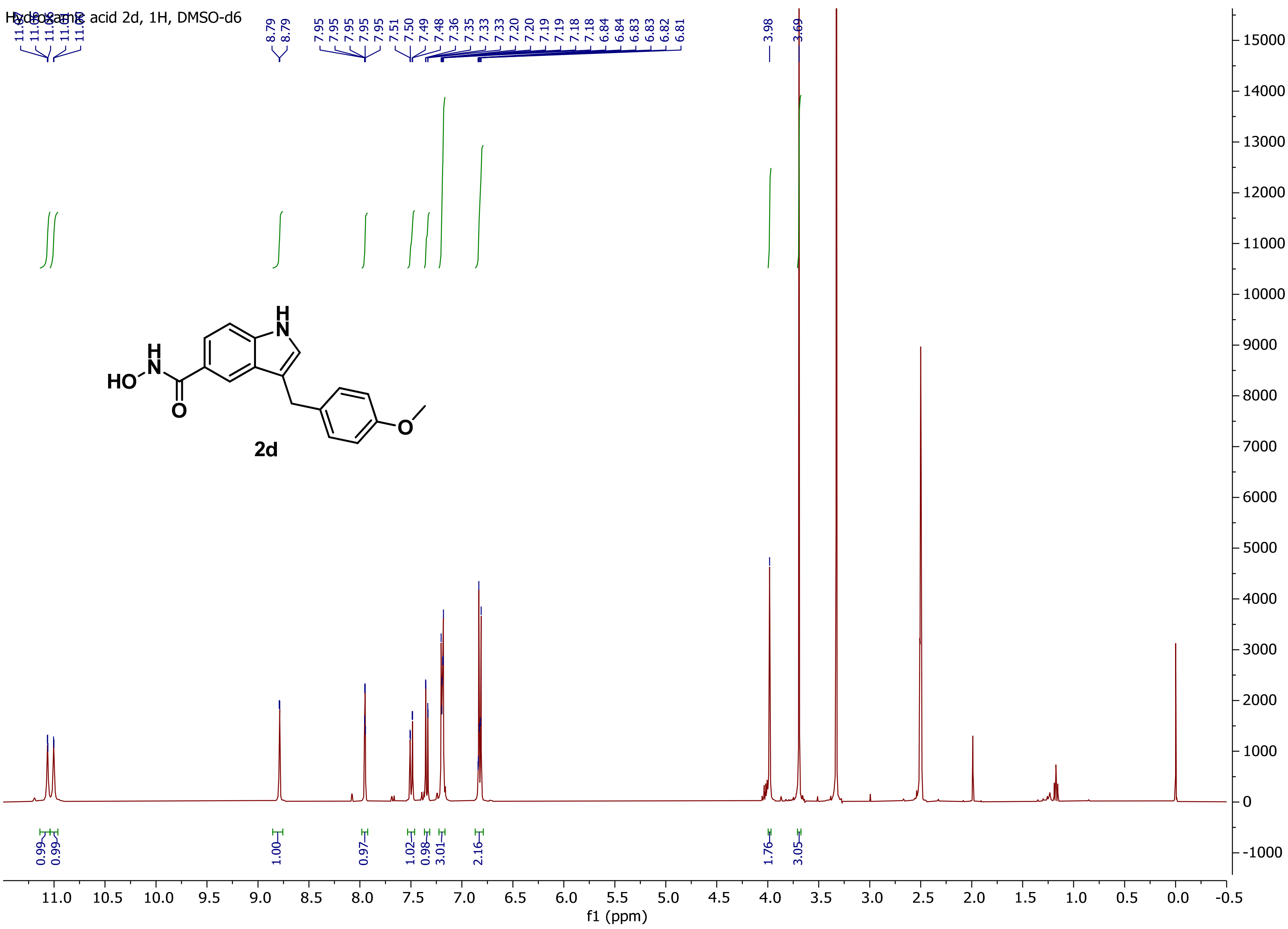
Hydroxamic acid 2d, 1H, DMSO-d6
8.79
8.79
7.95
7.95
7.95
7.95
7.95
7.51
7.50
7.49
7.48
7.36
7.35
7.33
7.33
7.20
7.20
7.19
7.19
7.18
7.18
6.84
6.84
6.83
6.83
6.82
6.81
3.98
3.69
HO
H
N
O
H
N
O
2d
0.99
0.99
1.00
0.97
1.02
0.98
3.01
2.16
1.76
3.05
f1 (ppm)

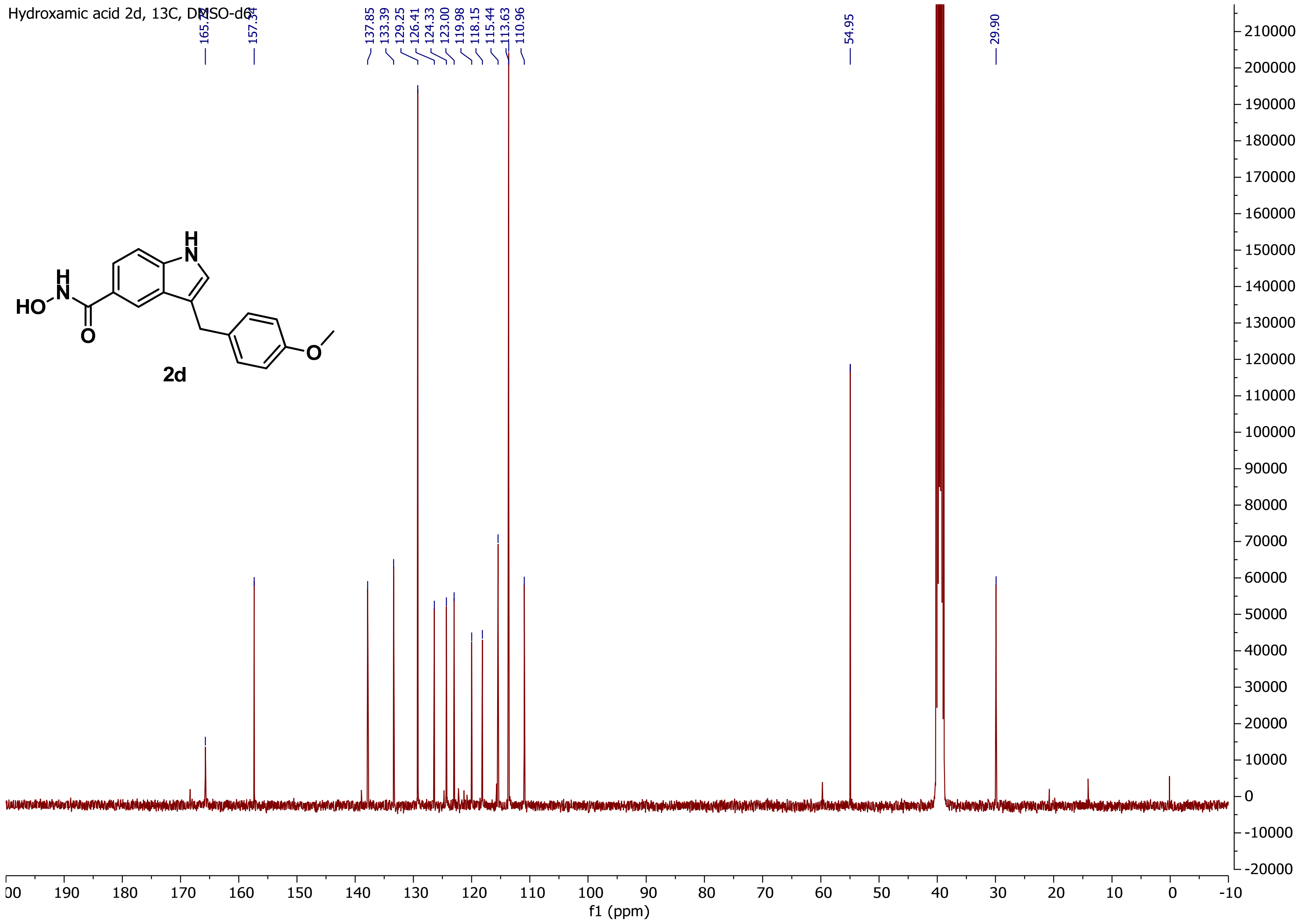
Hydroxamic acid 2d, 13C, DMSO-d6
157.34
137.85
133.39
129.25
126.41
124.33
123.00
119.98
118.15
115.44
113.63
110.96
54.95
29.90
HO
N
H
O
2d
f1 (ppm)

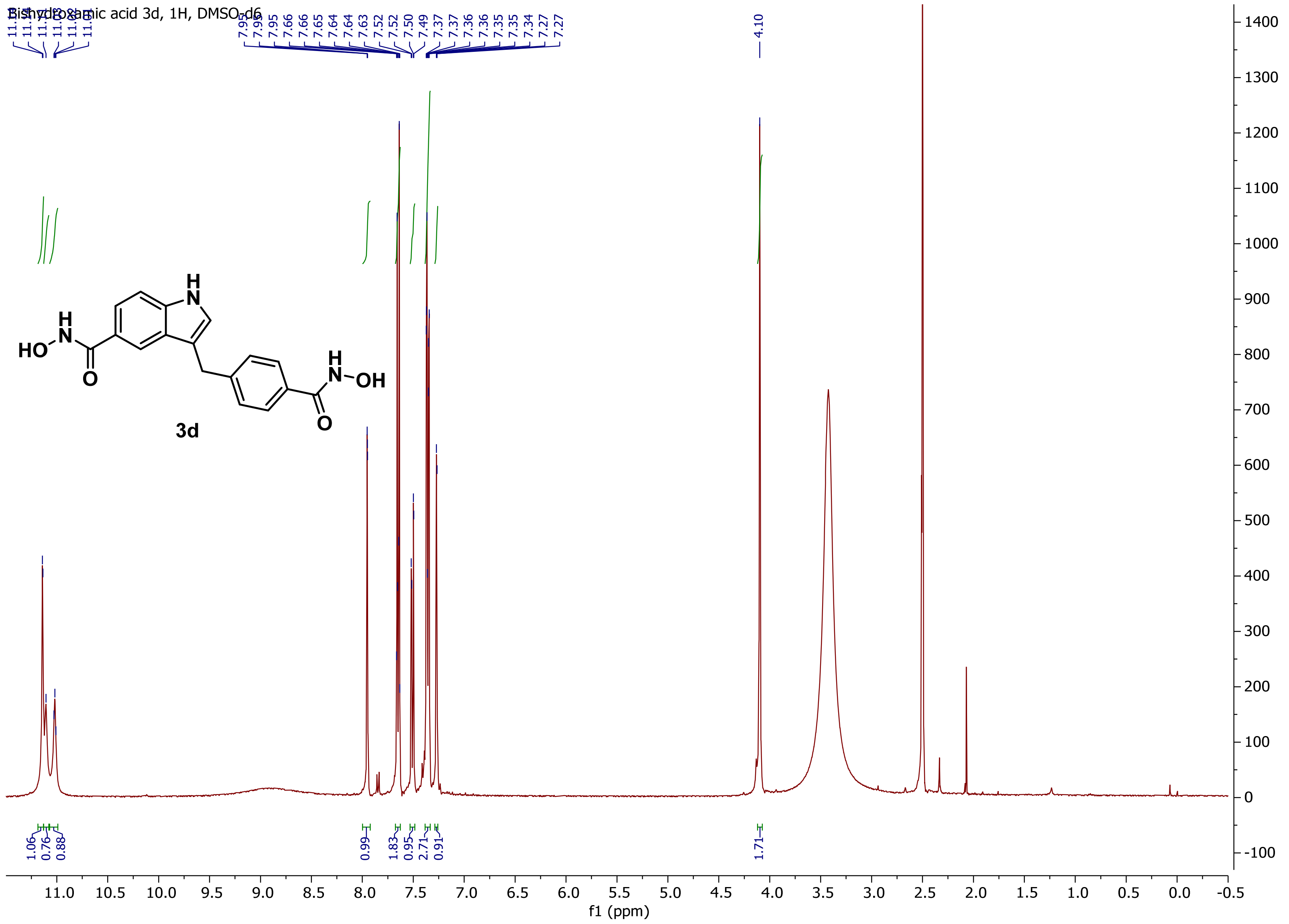
Bishydroxamic acid 3d, 1H, DMSO-d6
f1 (ppm)
3d

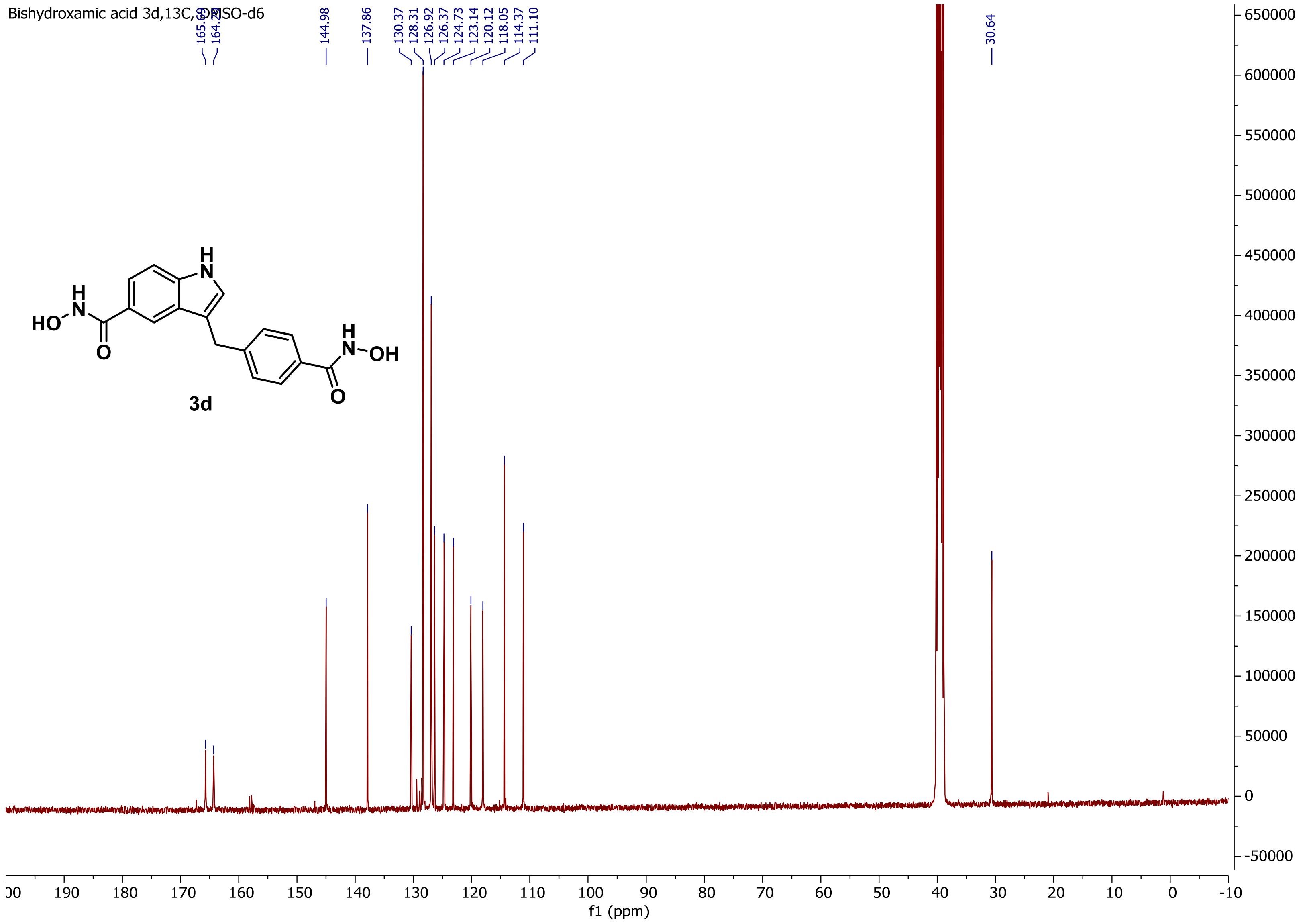
Bishydroxamic acid 3d,13C,DMSO-d6
165.69
164.29
144.98
137.86
130.37
128.31
126.92
126.37
124.73
123.14
120.12
118.05
114.37
111.10
30.64
3d
f1 (ppm)

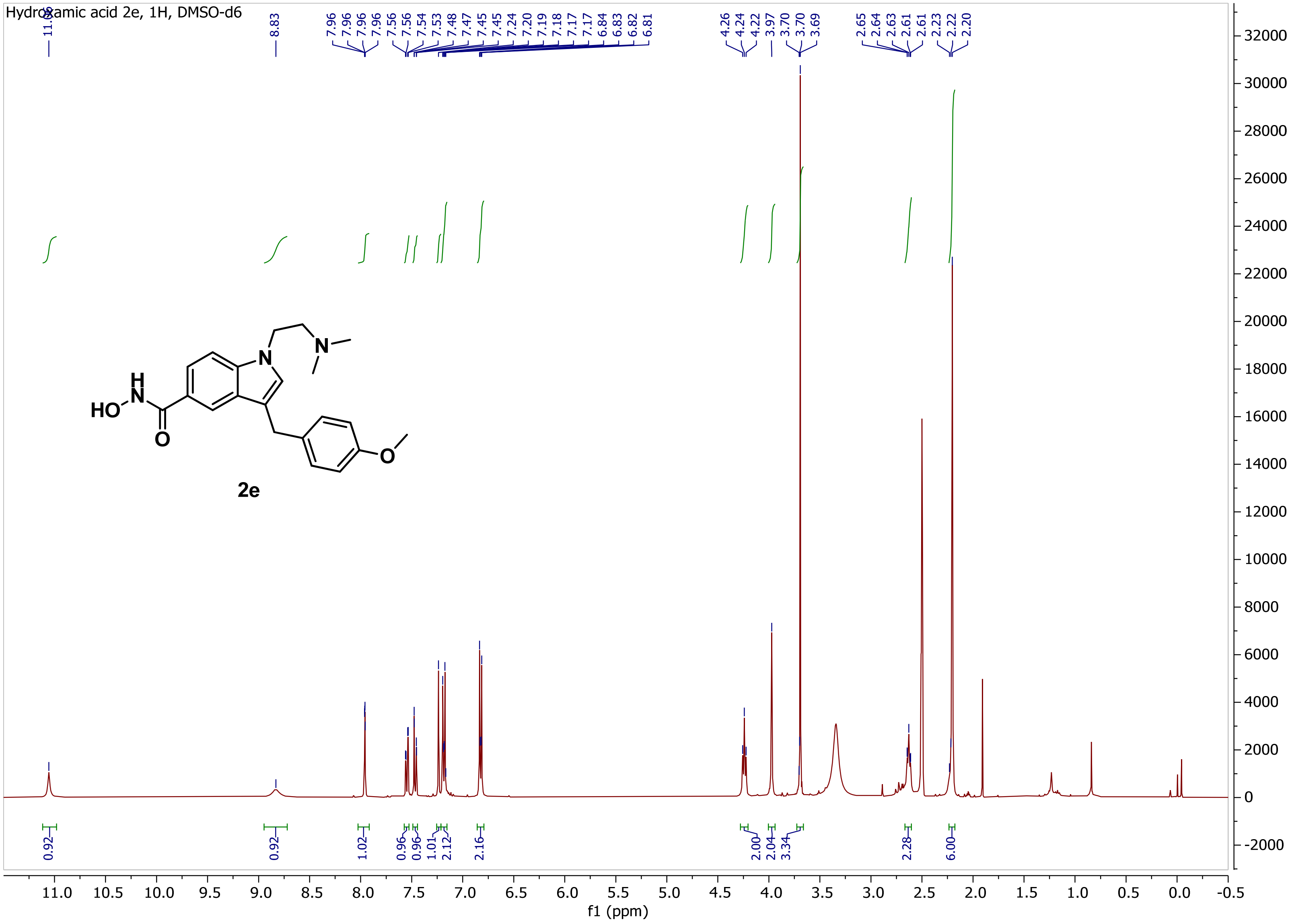

Hydroxamic acid 2e, 1H, DMSO-d6
11.08
8.83
7.96 7.96 7.96 7.96 7.56 7.56 7.54 7.53 7.48 7.47 7.45 7.45 7.24 7.20 7.19 7.18 7.17 7.17 6.84 6.83 6.82 6.81
4.26 4.24 4.22 3.97 3.70 3.70 3.69
2.65 2.64 2.63 2.61 2.61 2.23 2.22 2.20
HO
H
N
N
N
O
O
2e
0.92 0.92 1.02 0.96 0.96 1.01 2.12 2.16 2.00 2.04 3.34 2.28 6.00
f1 (ppm)

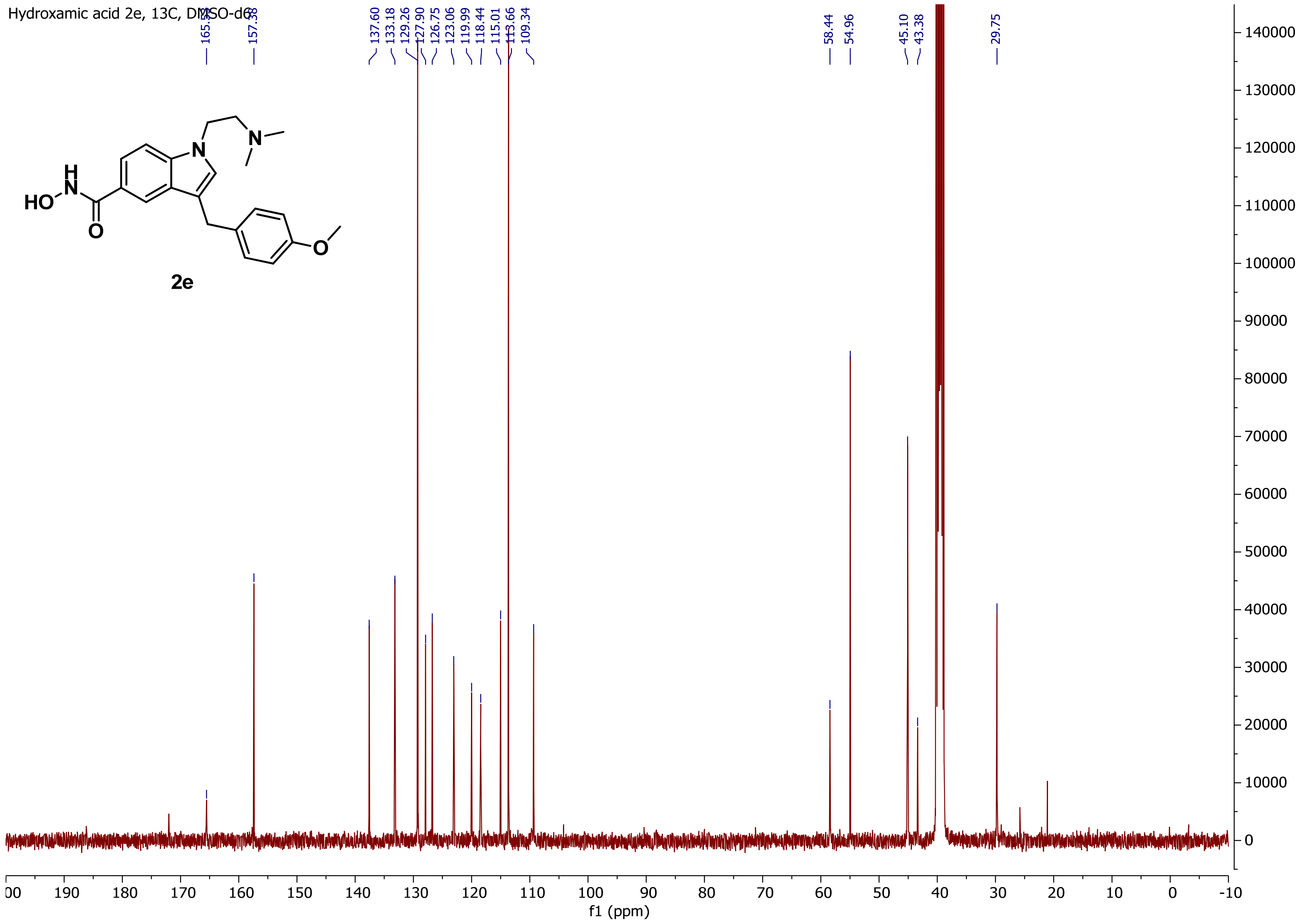

Hydroxamic acid 2e, 13C, DMSO-d6
157.38
137.60
133.18
129.26
127.90
126.75
123.06
119.99
118.44
115.01
113.66
109.34
58.44
54.96
45.10
43.38
29.75
HO
H
N
O
N
N
O
2e
140000
130000
120000
110000
100000
90000
80000
70000
60000
50000
40000
30000
20000
10000
0
190
180
170
160
150
140
130
120
110
100
90
80
70
60
50
40
30
20
10
0
-10
f1 (ppm)

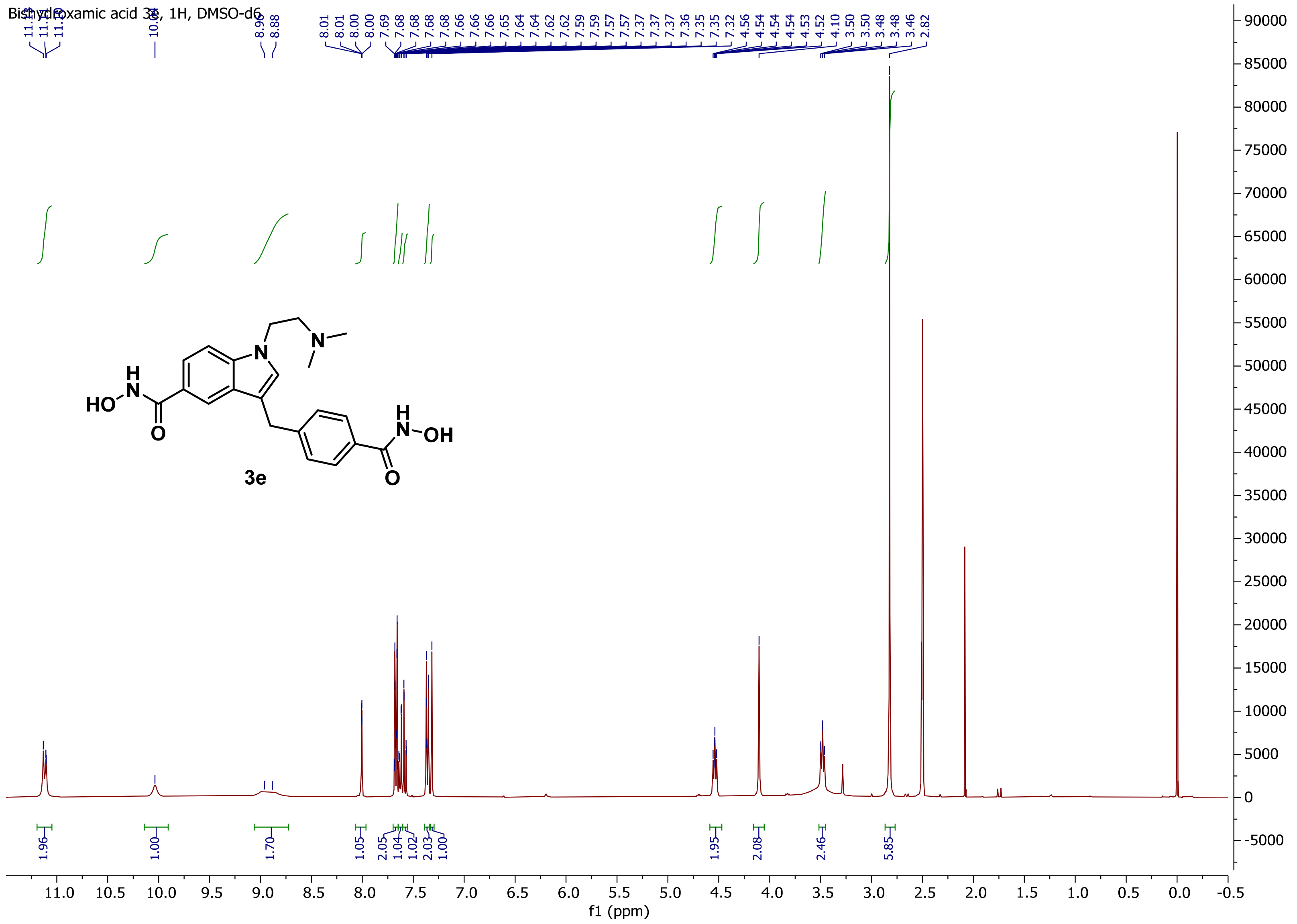
Bishydroxamic acid 3e, 1H, DMSO-d6
3e
f1 (ppm)

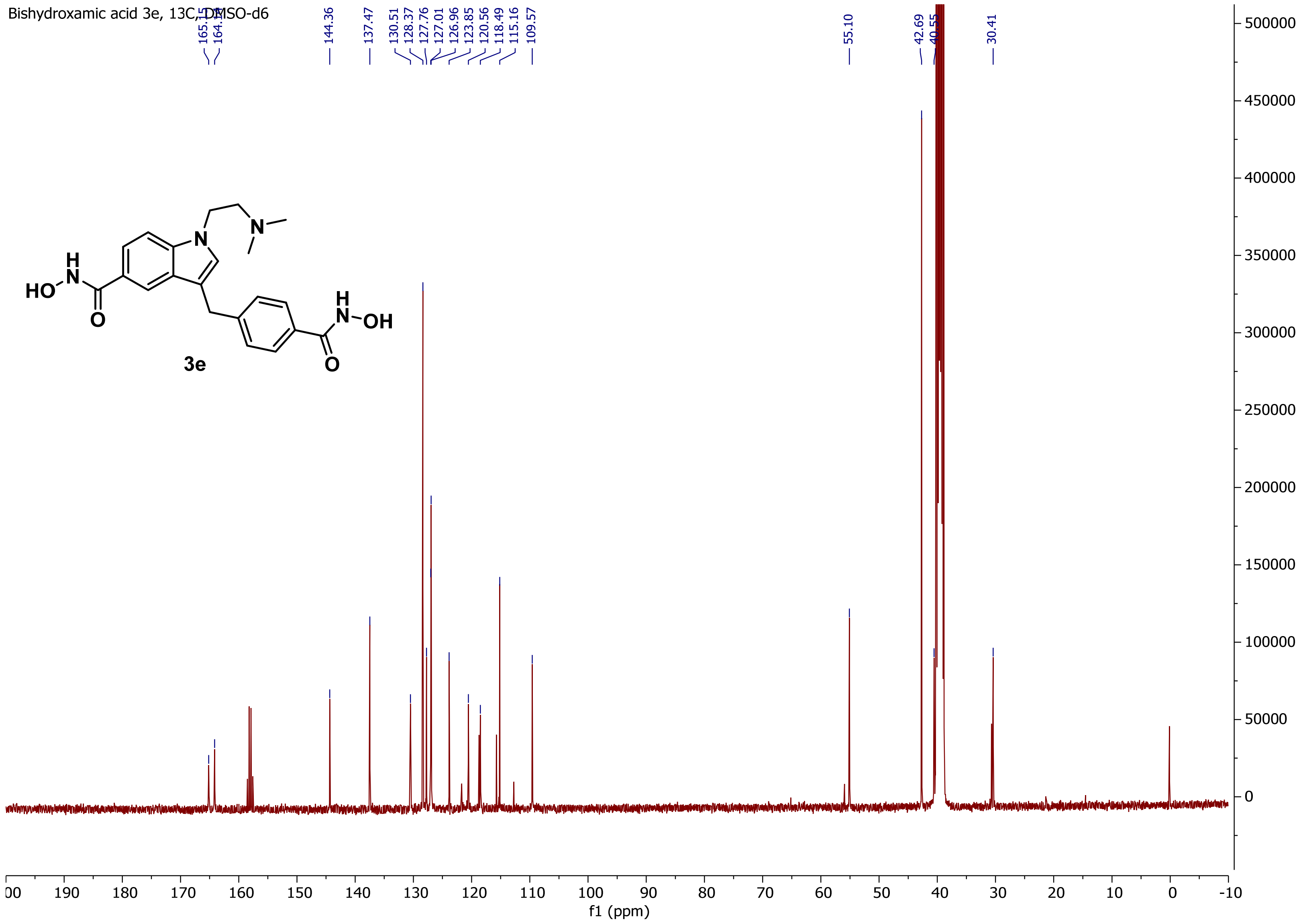
Bishydroxamic acid 3e, 13C, DMSO-d6
165.15
164.1
144.36
137.47
130.51
128.37
127.76
127.01
126.96
123.85
120.56
118.49
115.16
109.57
55.10
42.69
40.55
30.41
HO
N
H
O
N
N
H
N
OH
O
3e
f1 (ppm)

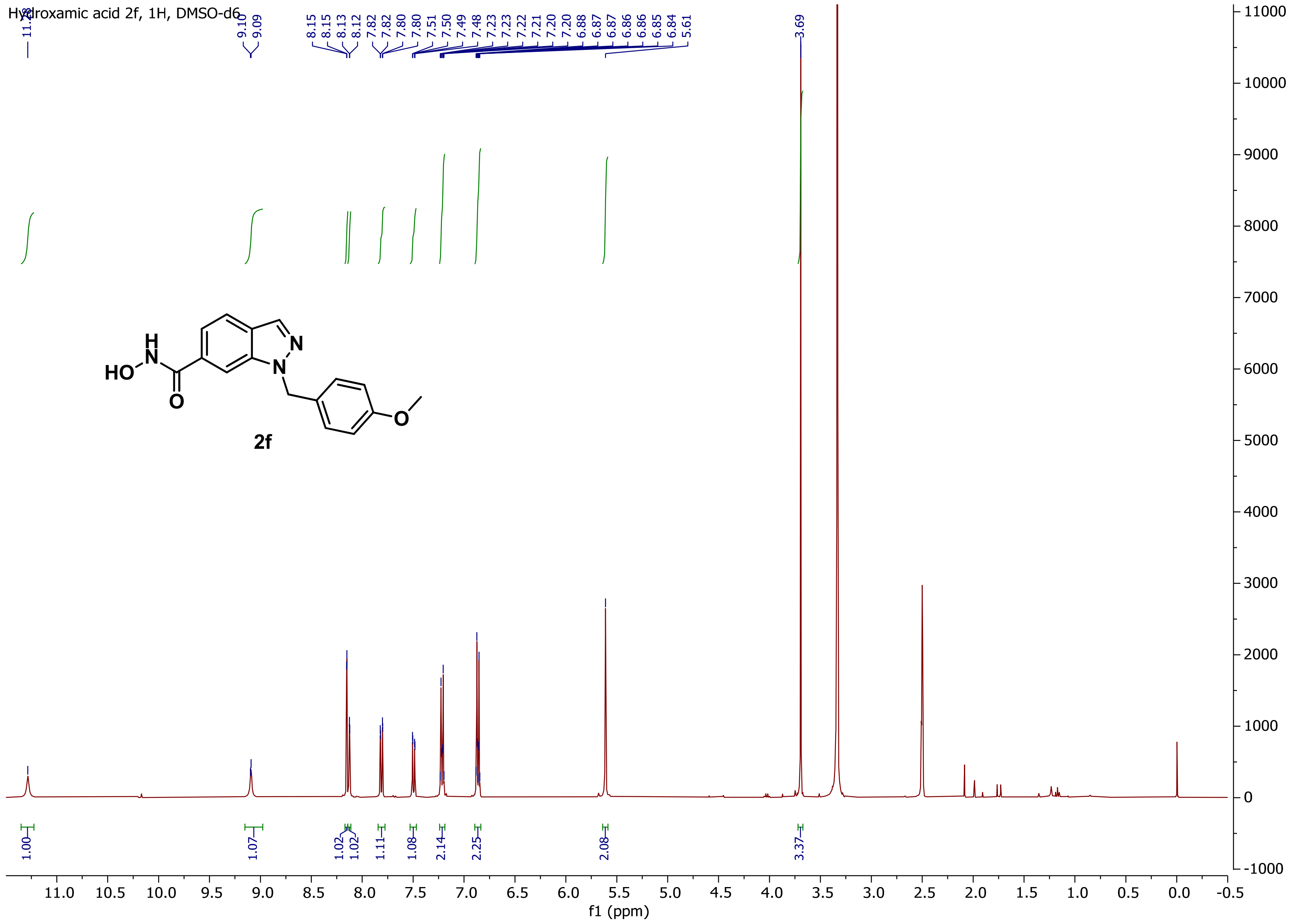
Hydroxamic acid 2f, 1H, DMSO-d6
HO
H
N
O
N
N
O
2f
f1 (ppm)

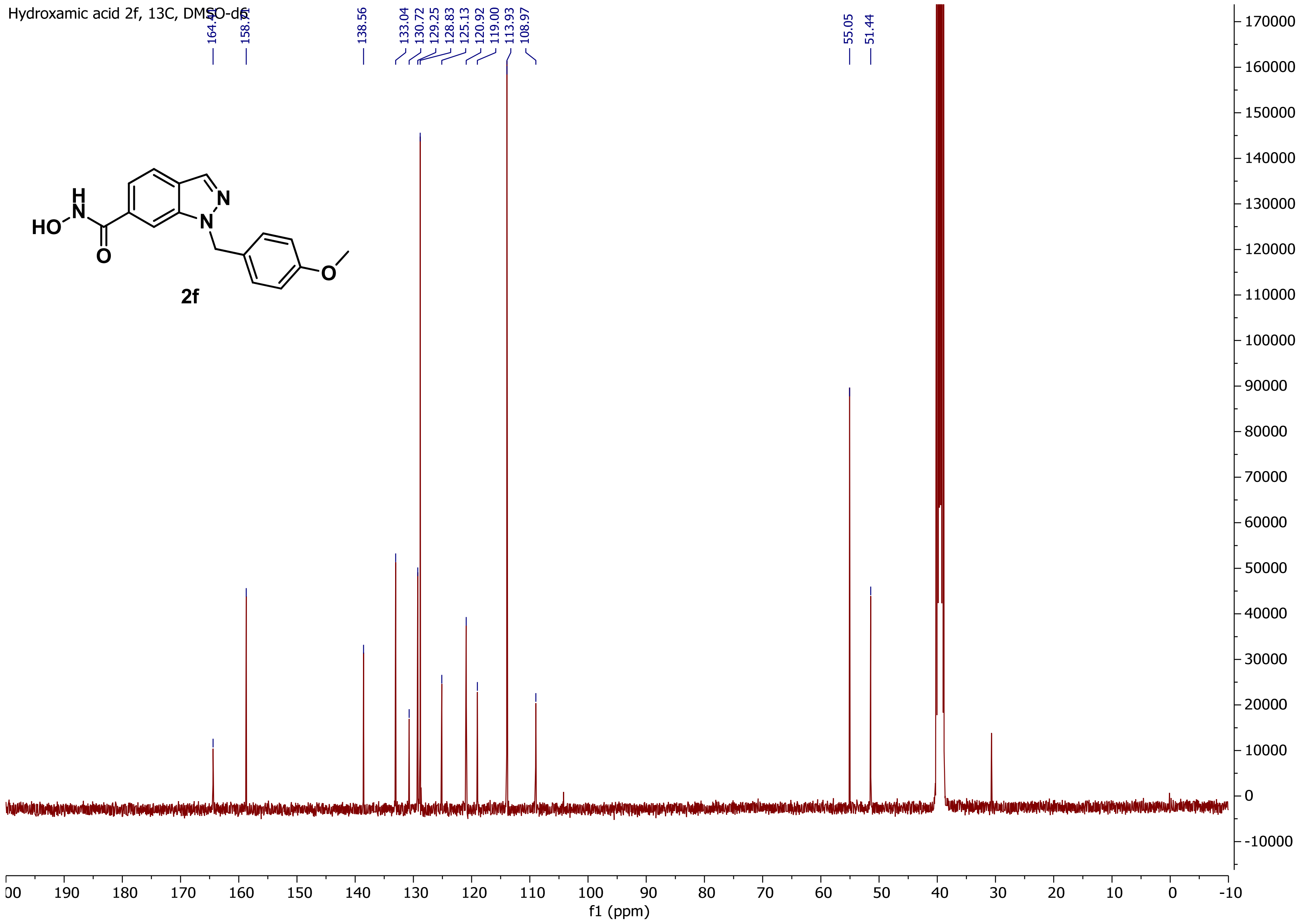
Hydroxamic acid 2f, 13C, DMSO-d6
164.40
158.7
138.56
133.04
130.72
129.25
128.83
125.13
120.92
119.00
113.93
108.97
55.05
51.44
HO
H
N
O
N
N
O
2f
170000
160000
150000
140000
130000
120000
110000
100000
90000
80000
70000
60000
50000
40000
30000
20000
10000
0
-10000
00
190
180
170
160
150
140
130
120
110
100
90
80
70
60
50
40
30
20
10
0
-10
f1 (ppm)

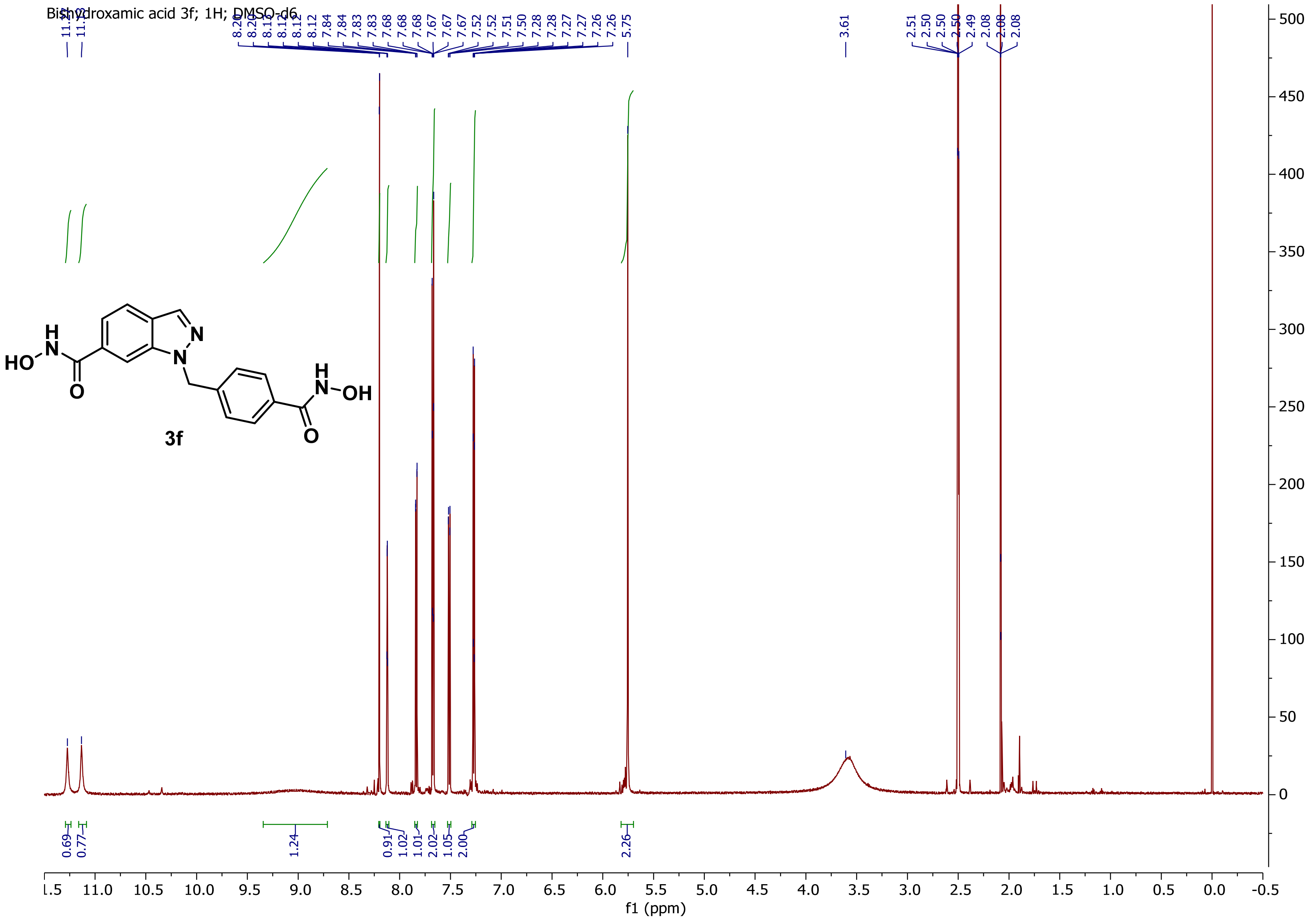
Bishydroxamic acid 3f; 1H; DMSO-d6
HO
N
H
O
N
N
H
N
OH
O
3f
f1 (ppm)

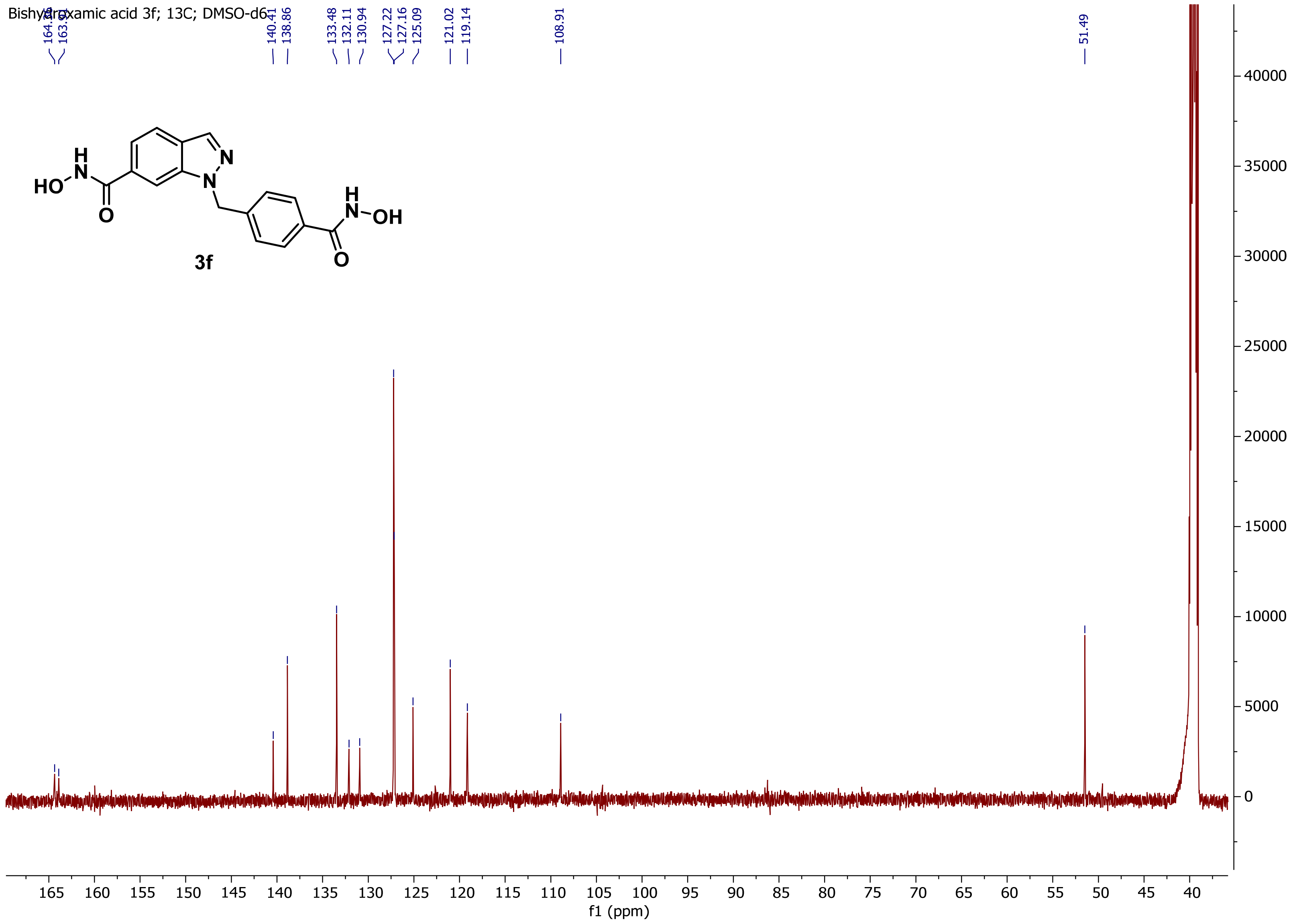
Bishydroxamic acid 3f; 13C; DMSO-d6
164.[illegible]
163.[illegible]
140.41
138.86
133.48
132.11
130.94
127.22
127.16
125.09
121.02
119.14
108.91
51.49
HO
N
H
O
N
N
H
N
OH
O
3f
40000
35000
30000
25000
20000
15000
10000
5000
0
165 160 155 150 145 140 135 130 125 120 115 110 105 100 95 90 85 80 75 70 65 60 55 50 45 40
f1 (ppm)

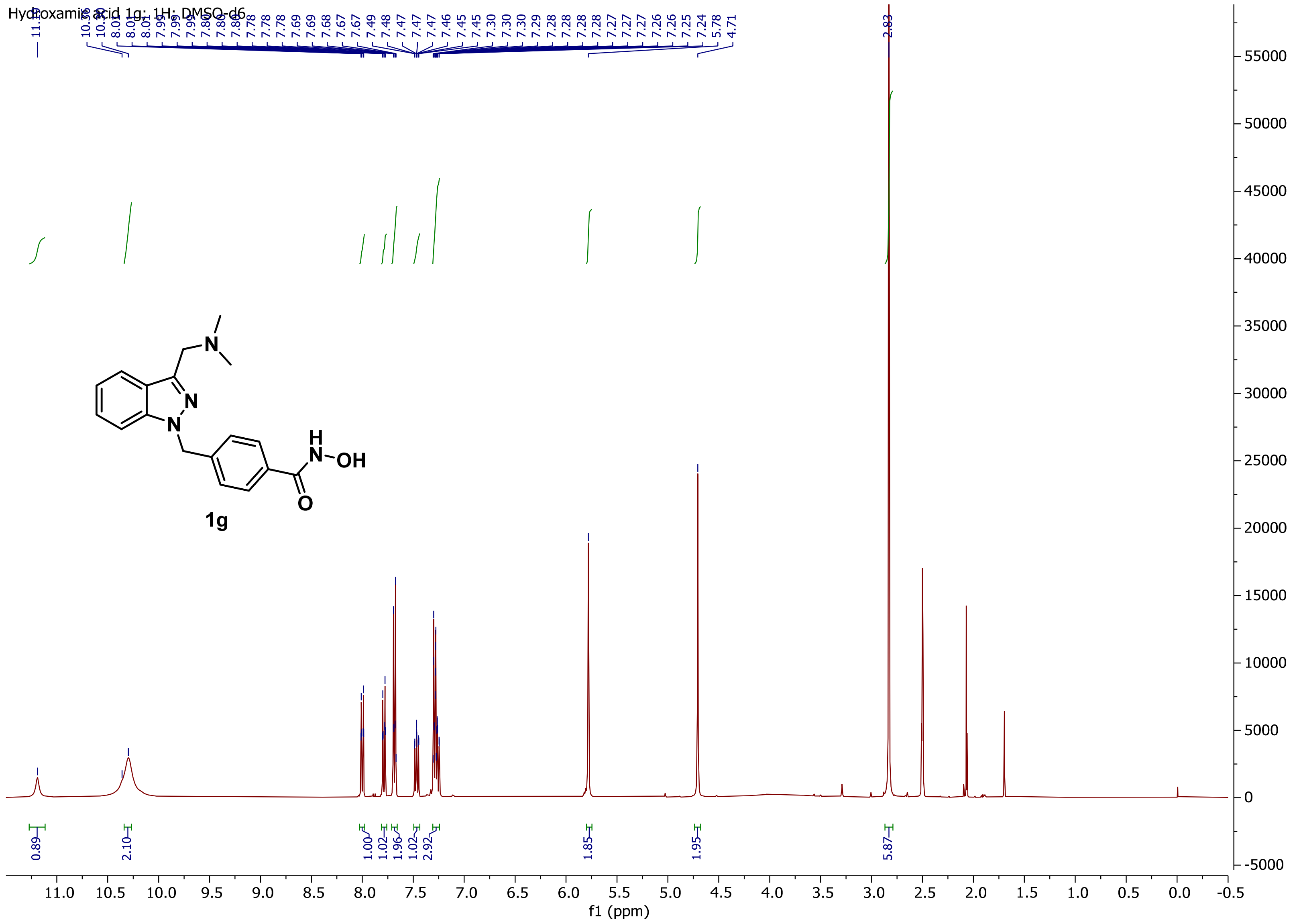
Hydroxamic acid 1g; 1H; DMSO-d6
1g
f1 (ppm)

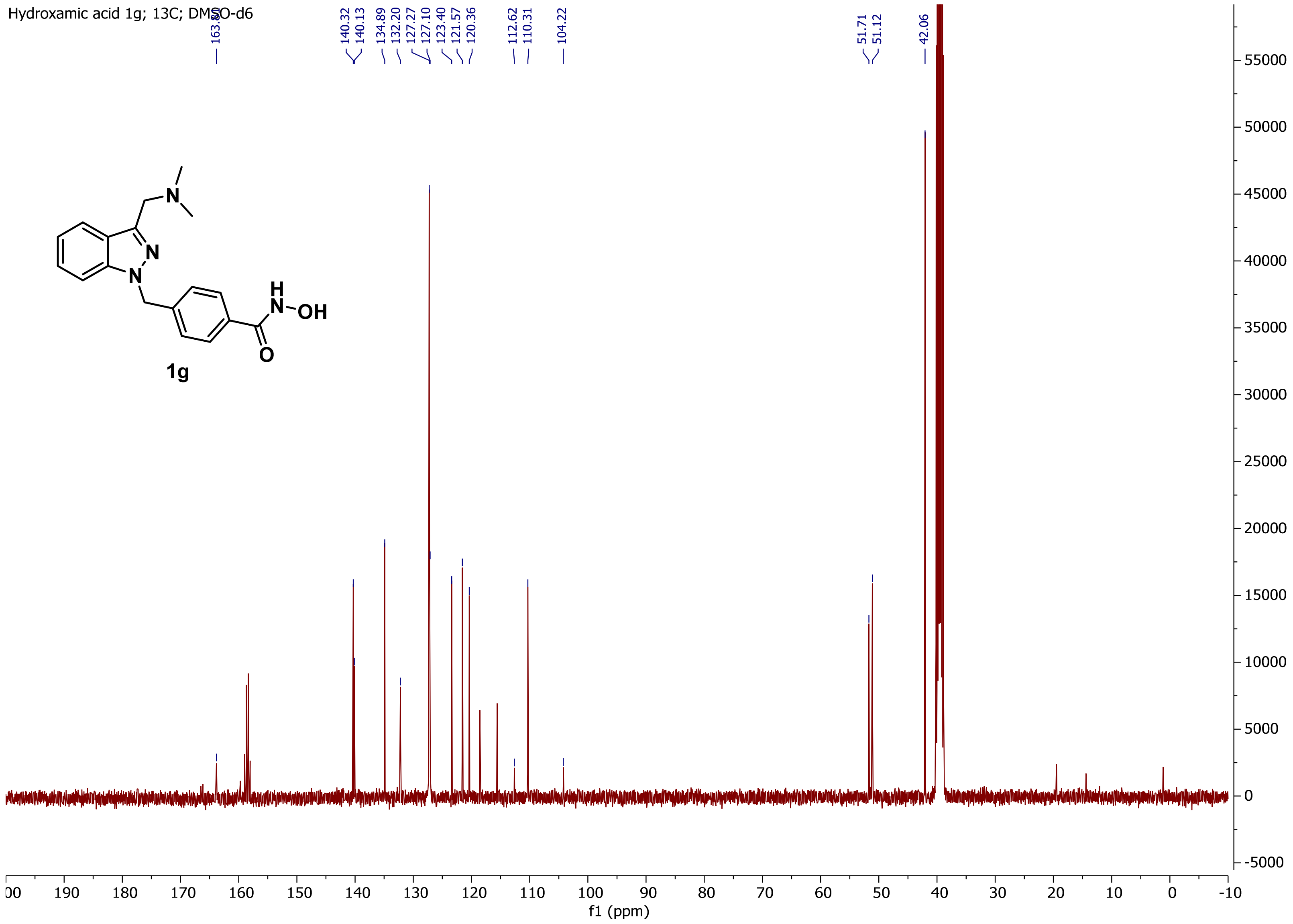
Hydroxamic acid 1g; 13C; DMSO-d6
163.80
140.32
140.13
134.89
132.20
127.27
127.10
123.40
121.57
120.36
112.62
110.31
104.22
51.71
51.12
42.06
N
N
N
H
N
OH
O
1g
55000
50000
45000
40000
35000
30000
25000
20000
15000
10000
5000
0
-5000
00
190
180
170
160
150
140
130
120
110
100
90
80
70
60
50
40
30
20
10
0
-10
f1 (ppm)

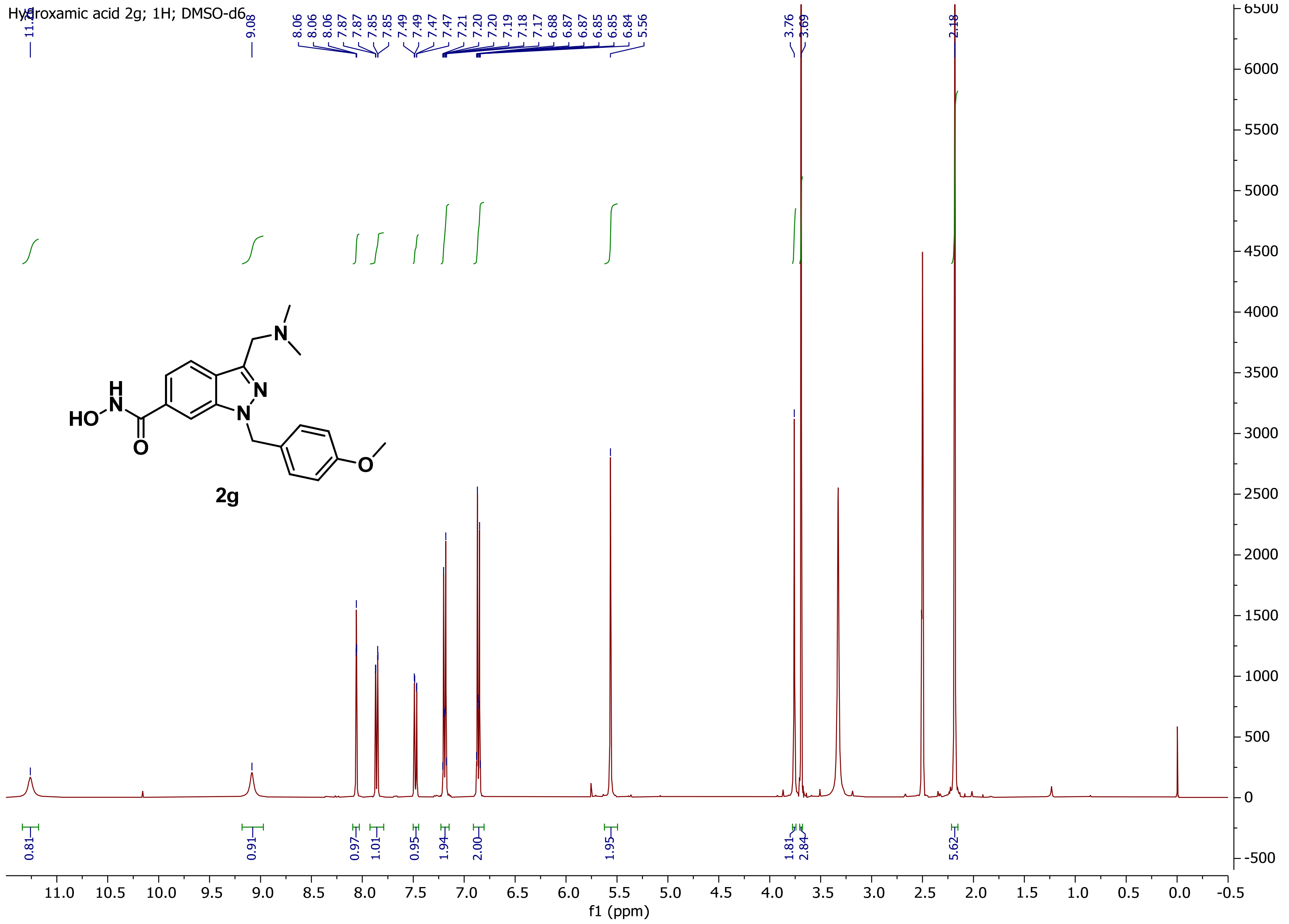
Hydroxamic acid 2g; 1H; DMSO-d6
11.26
9.08
8.06
8.06
8.06
7.87
7.85
7.85
7.49
7.49
7.47
7.47
7.21
7.20
7.19
7.18
7.17
6.88
6.87
6.87
6.85
6.85
6.84
5.56
3.76
3.69
2.18
HO
H
N
O
N
N
N
O
2g
0.81
0.91
0.97
1.01
0.95
1.94
2.00
1.95
1.81
2.84
5.62
f1 (ppm)

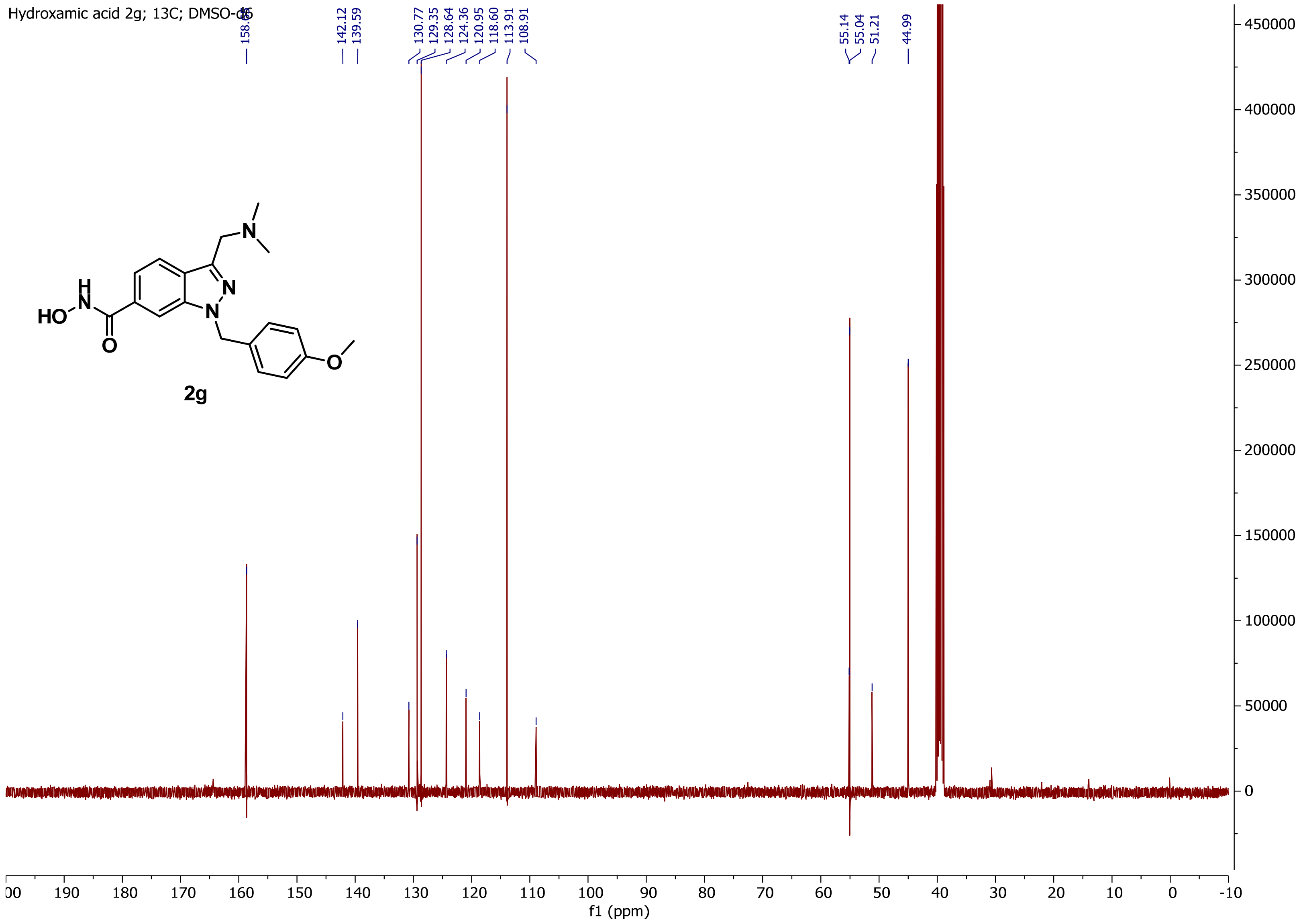
Hydroxamic acid 2g; 13C; DMSO-d6
158.66
142.12
139.59
130.77
129.35
128.64
124.36
120.95
118.60
113.91
108.91
55.14
55.04
51.21
44.99
HO
N
H
O
N
N
N
O
2g
f1 (ppm)

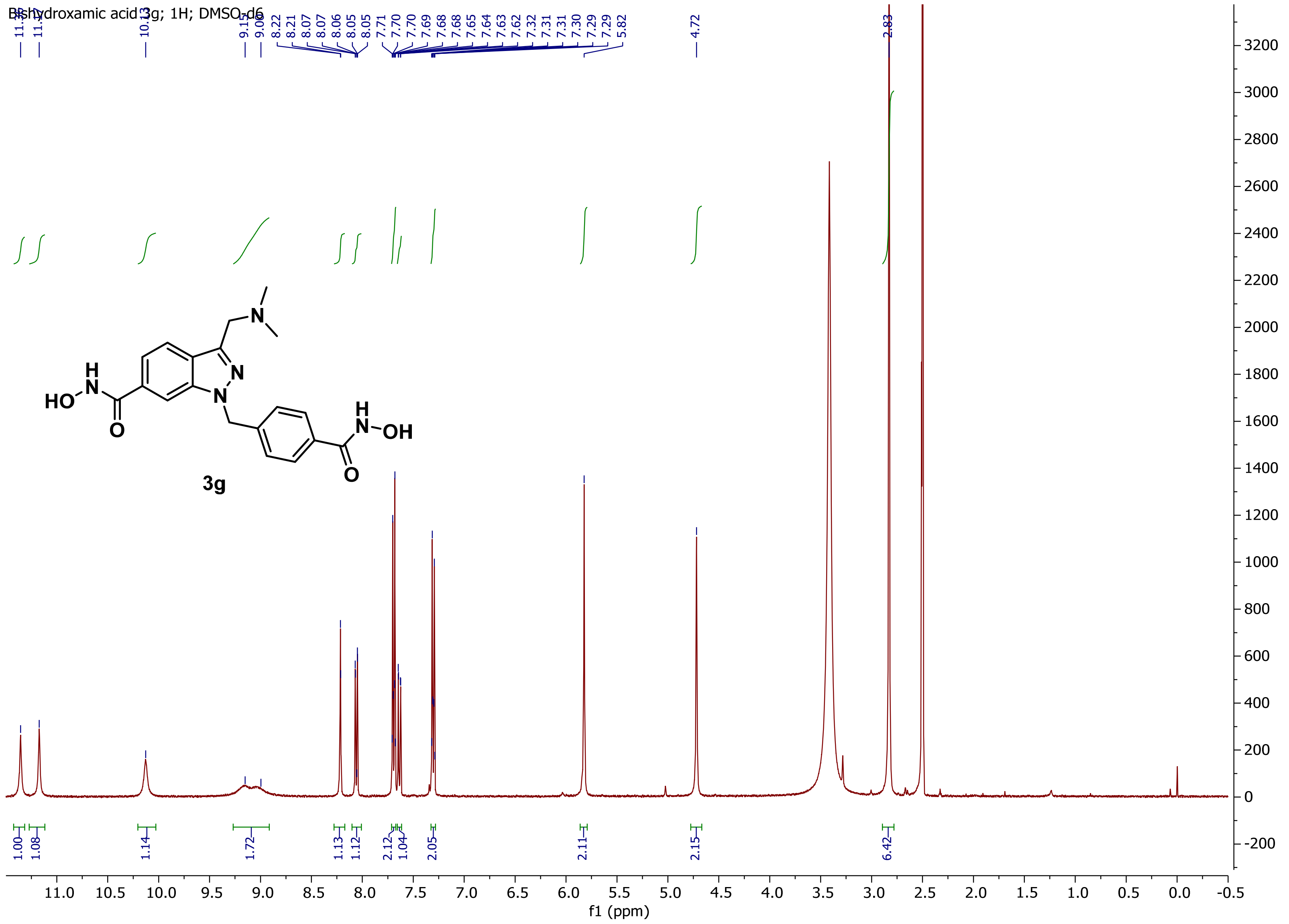
Bishydroxamic acid 3g; 1H; DMSO-d6
3g
f1 (ppm)

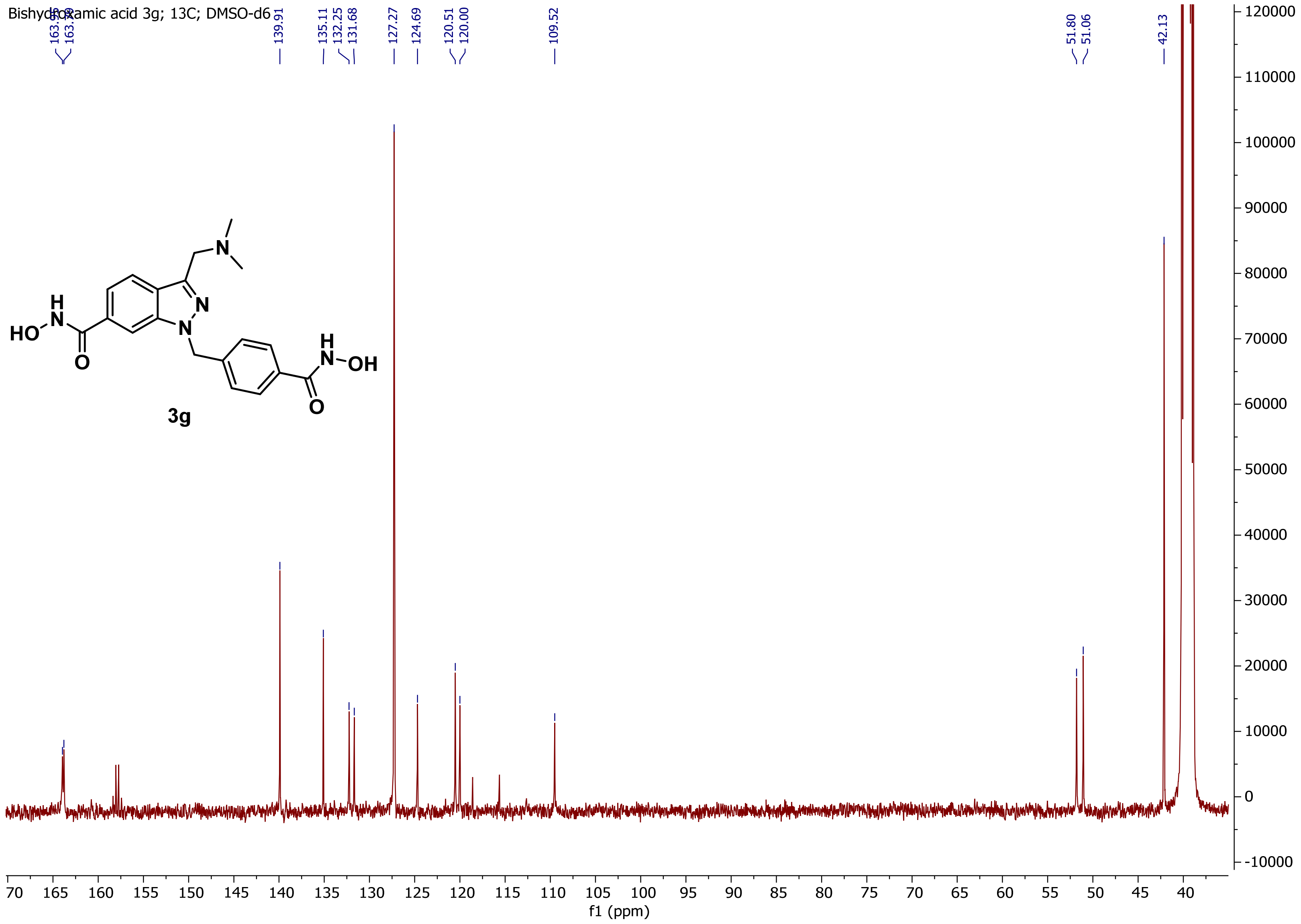

Bishydroxamic acid 3g; 13C; DMSO-d6
163.99
139.91
135.11
132.25
131.68
127.27
124.69
120.51
120.00
109.52
51.80
51.06
42.13
HO
N
H
O
N
N
N
N
H
OH
O
3g
f1 (ppm)
